# The circadian clock mutant *lhy cca1 elf3* paces starch mobilization to dawn despite severely disrupted circadian clock function

**DOI:** 10.1093/plphys/kiac226

**Published:** 2022-05-14

**Authors:** Thiago Alexandre Moraes, Virginie Mengin, Bruno Peixoto, Beatrice Encke, Nicole Krohn, Melanie Höhne, Ursula Krause, Mark Stitt

**Affiliations:** Max Planck Institute of Molecular Plant Physiology, Potsdam-Golm 14476, Germany; School of Life Sciences, University of Essex, Colchester CO4 3SQ, UK; Instituto Gulbenkian de Ciência, Oeiras 2780-156,Portugal; GREEN-IT Bioresources for Sustainability, ITQB NOVA, Oeiras 2780-157,Portugal; Systematic Botany and Biodiversity, Humboldt University of Berlin, Berlin D-10115, Germany; Abteilung für Parodontologie und Synoptische Zahnmedizin, Charité Universitätsmedizin, Berlin 14197, Germany; Max Planck Institute of Molecular Plant Physiology, Potsdam-Golm 14476, Germany; Max Planck Institute of Molecular Plant Physiology, Potsdam-Golm 14476, Germany; Max Planck Institute of Molecular Plant Physiology, Potsdam-Golm 14476, Germany

## Abstract

Many plants, including Arabidopsis (*Arabidopsis thaliana*), accumulate starch in the daytime and remobilize it to support maintenance and growth at night. Starch accumulation is increased when carbon is in short supply, for example, in short photoperiods. Mobilization is paced to exhaust starch around dawn, as anticipated by the circadian clock. This diel pattern of turnover is largely robust against loss of day, dawn, dusk, or evening clock components. Here, we investigated diel starch turnover in the triple circadian clock mutant *lhy cca1 elf3*, which lacks the *LATE ELONGATED HYPOCOTYL* and the *CIRCADIAN CLOCK-ASSOCIATED1* (*CCA1*) dawn components and the *EARLY FLOWERING3* (*ELF3*) evening components of the circadian clock. The diel oscillations of transcripts for the remaining clock components and related genes like *REVEILLE* and *PHYTOCHROME-INTERACING FACTOR* family members exhibited attenuated amplitudes and altered peak time, weakened dawn dominance, and decreased robustness against changes in the external light–dark cycle. The triple mutant was unable to increase starch accumulation in short photoperiods. However, it was still able to pace starch mobilization to around dawn in different photoperiods and growth irradiances and to around 24 h after the previous dawn in T17 and T28 cycles. The triple mutant was able to slow down starch mobilization after a sudden low-light day or a sudden early dusk, although in the latter case it did not fully compensate for the lengthened night. Overall, there was a slight trend to less linear mobilization of starch. Thus, starch mobilization can be paced rather robustly to dawn despite a major disruption of the transcriptional clock. It is proposed that temporal information can be delivered from clock components or a semi-autonomous oscillator.

## Teaser

Starch mobilization robustly paces to dawn despite severe disruption of the transcriptional circadian oscillator.

## Introduction

Metabolism and growth in the daytime are driven by photosynthetic carbon (C) fixation but at night depend on reserves accumulated in previous light periods (Smith and Stitt, 2007). In many plants, including Arabidopsis (*Arabidopsis thaliana*), leaf starch is the major transitory C reserve. Diel starch turnover is exquisitely regulated. A larger proportion of the fixed C is accumulated as starch when less C is available over the entire 24-h cycle, for example in short photoperiods or low irradiance (Smith and Stitt, 2007; Mugford et al., 2014; Sulpice et al., 2014; Mengin et al., 2017; Smith and Zeeman, 2020). This is partly due to daytime growth being restricted in short photoperiods, leading to higher levels of sugars in leaves (Gibon et al., 2004; Stitt et al., 2010; Sulpice et al., 2014; Mengin et al., 2017; Moraes et al., 2019). At night, mobilization is regulated such that starch is almost but not completely exhausted at dawn. Mobilization is robustly paced to dawn across a wide range of growth conditions, and even after sudden perturbations like a day of low irradiance, an early dusk, a light break in the middle of the night, or a change in night temperature (Graf et al., 2010; Pyl et al., 2012; Scialdone et al., 2013; Sulpice et al., 2014; Pilkington et al., 2015; Feike et al., 2016; Mengin et al., 2017; Moraes et al., 2019; Smith and Zeeman, 2020). This pattern of starch turnover optimizes growth. On the one hand, it ensures that fixed C is used for growth within a single 24-h cycle, and spreads use of heavy-investment machinery like ribosomes over the entire cycle. On the other hand, it avoids deleterious intervals of starvation at the end of the night (Stitt and Zeeman, 2012; Pal et al., 2013; Sulpice et al., 2014; Ishihara et al., 2015; Mengin et al., 2017; Sharkey, 2017).

The circadian clock is involved in the regulation of diel starch turnover (Graf et al., 2010; Scialdone et al., 2013; Pokhilko et al., 2014; Seki et al., 2017; Flis et al., 2019; Webb et al., 2019; Smith and Zeeman, 2020). The strongest evidence is for starch mobilization. In wild-type plants mobilization is paced to exhaust starch about 24 h after the previous dawn, even when plants are grown in 17-h or 28-h light/dark cycles (Graf et al., 2010). A 24-h rhythmicity is typical for processes that are under circadian (*circa diem*) regulation. Many clock mutants show a modified pattern of starch mobilization, with starch being exhausted before dawn in *lhy cca1*, and incompletely exhausted at dawn in *PSEUDO-RESPONSE REGULATOR* (*prr7 prr9*), *elf3*, and *GIGANTEA* (*gi*; Graf et al., 2010; Scialdone et al., 2013; Flis et al., 2019). This often matches the time at which transcript abundance for dawn components like *LHY* and *CCA1* is at peak values in a light–dark cycle (*elf3*, *gi*, also *prr7 prr9* if not only the time when transcript abundance rises but also when it starts to decline is considered; Flis et al., 2019). In some cases the time when starch is exhausted also matches changes in clock period as determined in free-running conditions (*lhy cca1*, *prr7 prr9*, *gi*; Alabadi et al., 2001; Mizoguchi et al., 2002; Farré et al., 2005; Nakamichi et al., 2005; Salomé and McClung, 2005; Martin-Tryon et al., 2006). However, these relationships are not universal. For example, the short period *toc1-2* mutant (*TIMING OF CAB EXPRESSION 1*; Somers et al., 1998) does not show marked premature starch exhaustion (StEx; Graf et al., 2010; Flis et al., 2019), and the long period *ZEITLUPE* mutant (Somers et al., 2004) mobilizes starch in a similar manner to wild-type plants (Graf et al., 2010). This might reflect differing contributions of clock components to the output that regulates starch mobilization (Graf et al., 2010).

The contribution of the circadian clock to the regulation of starch accumulation is less well established. Compared to wild-type plants, the *prr7 prr9* and *elf3* mutants show slower starch accumulation, despite having higher daytime sugar levels (Flis et al., 2019). However, these and most other clock mutants are still able to increase allocation of C to starch in short photoperiods compared to long photoperiods, although not always as strongly as in wild-type plants (Mugford et al., 2014).

The Arabidopsis clock is an interactive network of “dawn”, “day” and “dusk,” and “evening” components (Nakamichi, 2011; Pokhilko et al., 2012; Fogelmark and Troein, 2014; Millar, 2016). The 24-h cycle starts with peak expression of dawn genes (*LHY*, *CCA1*), followed by day (*PRR9*, *PRR7*), dusk (*PRR5*, *TOC1*, and *GI*), and evening (*ELF3*, *ELF4*, *LUX ARRHYTHMO* [*LUX*]; collectively forming the Evening Complex [EC]) genes, which repress outputs like the *PHYTCHROME-INTERACTING FACTORS* (*PIF*s) 4 and 5 and the day genes. Later in the cycle, dusk and evening genes decay or self-repress, and *LHY* and *CCA1* expression rises to peak around the next dawn. In addition, three members of the *REVEILLE* (*RVE*) family, *RVE4*, *RVE*6, and *RVE*8 positively regulate clock components, especially dusk and evening genes such as *TOC1* and *LUX* (Farinas and Mas, 2011; Rawat et al., 2011; Hsu et al., 2013; Shalit-Kaneh et al., 2018). Many clock components are also regulated directly or indirectly by phytochrome (Yeom et al., 2014; Shor et al., 2017, 2018; Liu et al., 2020). An important feature of the Arabidopsis clock is that the timing of peak expression depends mainly on time elapsed after dawn (Seaton et al., 2015; Song et al., 2015) and is only weakly affected by when dusk occurs (Fowler, 1999; Matsushika et al., 2000; Edwards et al., 2010; Flis et al., 2016). This is termed “dawn dominance.”

Regulation of diel starch turnover presumably requires integration of information about time and metabolic status. Different models have been advanced to explain how this might occur. The metabolic feedback model postulates that starch turnover is regulated to maintain sugar homeostasis; signals related to sugar status modify clock gene expression, clock phase, and output signals, which in turn affect starch accumulation and mobilization (Feugier and Satake, 2013; Seki et al., 2017; Webb et al., 2019). The postulated sugar input and clock outputs are unknown except that one input may involve regulation of *PRR7* expression by *BASIC LEUCINE ZIPPER 63* (*bZIP63*; Haydon et al., 2013; Frank et al., 2018). It was recently shown that *bzip63* mutants exhaust their starch prematurely, adding to the evidence that this input plays a role in regulating diel starch turnover (Viana et al., 2021). The arithmetic division model proposes that information about the amount of starch (*S*) and time to dawn (*T*) is integrated to set the rate of starch mobilization (*R*) such that starch is exhausted at dawn (i.e. *R = S/T*; Scialdone et al., 2013; Scialdone and Howard, 2015). The molecular identity of S and T and the mechanism by which the circadian clock generates T are unknown (Seaton et al., 2014, 2015). The arithmetic division model does not explicitly address starch accumulation.

In both types of model, the circadian oscillator provides a temporal input. This input might derive from a specific clock component, or be an integrated clock output or even involve a semi-autonomous oscillator. Two observations make it unlikely that the temporal input derives from a single clock component. The first is that starch mobilization is timed to dawn over wide range of photoperiods (from 4 to 18 h; Gibon et al., 2009; Sulpice et al., 2014; Flis et al., 2016). This is difficult to reconcile with an output from a specific clock gene. These show relatively sharp peaks of expression that trigger an event at a critical time in the 24-h cycle, as occurs in the coincidence pathway of photoperiod sensing (Turck et al., 2008; Golembeski et al., 2014) but are less well suited to facilitate a progressive response through most of the 24-h cycle. A second observation is that the wild-type pattern of starch turnover is retained in most previously studied clock mutants. Although some mutants mobilize their starch too quickly or too slowly, this often matches the time of when dawn-phased transcripts peak (e.g. *LHY*, *CCA1*, *GRANULE BOUND STARCH SYNTHASE 1* [*GBSS1*]) in that mutant (see above). Crucially, mutants lacking dawn, day, dusk, or evening clock components are still able to slow down mobilization after a sudden early dusk (Flis et al., 2019).

It is possible that the temporal input is generated by an interaction between two or more clock components whose expression peaks are separated in time, for example dawn and evening components (Flis et al., 2019). It has also been proposed that inclusion of light-gating might stabilize the temporal input against mutations in the clock (Seaton et al., 2014). “Light-gating” refers to the diel regulation of biological responses in such manner that that the response to a given stimulus is stronger (or possible only) during the light period. Studies of the *prr7 prr9* double mutant point to the potential complexity of the temporal output. This mutant exhibits a long period in free running light (Farré et al., 2005; Nakamichi et al., 2005; Salomé and McClung, 2005). It does not exhaust its starch until ∼28 h after the previous dawn (Flis et al., 2019), consistent with the idea (Graf et al., 2010; Scialdone et al., 2013) that clock period may influence the rate of starch mobilization. However, when *prr7 prr9* was grown in a light–dark cycle, expression of clock genes is only delayed for the first part of the 24-h cycle, and accelerates after darkening with the result that “dawn” transcripts peak about 24 h after the previous dawn, that is, like wild-type plants (Flis et al., 2015, 2019). The slow starch mobilization might reflect a complex interaction between the remaining clock components in the *prr7 prr9* background. It is also consistent with the temporal input being generated early in the 24-h cycle, possibly via a clock output that triggers a semi-autonomous signal that decays during the remainder of the 24-h cycle.

The rather robust response of diel starch turnover to lesions in individual clock components prompted us to investigate starch turnover in a higher-order mutant with an even more severe disturbance of circadian function. For this purpose, a mutant should meet two criteria. First, as growth in a light/dark cycle is a prerequisite to study diel starch turnover, the mutant should exhibit severely disturbed circadian clock function in light–dark cycles. Second, the mutant should not exhibit strong stress responses, and should have a reasonable rate of growth that would be expected to exhaust its starch by dawn. High levels of starch would introduce possible complications due to starch mobilization being restricted by the capacity of the breakdown pathway (Seaton et al., 2014) or feedback inhibition due to incomplete use of the products of starch mobilization (Martins et al., 2013; dos Anjos et al., 2018). We therefore excluded the *prr9 prr7 prr5* mutant, which has strongly activated stress responses, aberrant growth and high starch (Nakamichi et al., 2009; Ruts et al., 2012). The triple *lhy cca1 elf3* mutant lacks functional dawn and evening clock components, and has been reported to exhibit largely unvarying expression of *PRR7*, *GI*, and *TOC1* throughout a 24-h light–dark cycle and a strongly modified pattern of *PRR9* expression (Dixon et al., 2011). We asked whether this mutant can maintain a wild-type pattern of diel starch turnover when it is grown in different light intensities and photoperiods, in different T-cycles, and in the face of sudden perturbations of the light regime.

## Results

### Diel starch turnover in a 12-h light/12-h dark photoperiod

**Figure 1:**
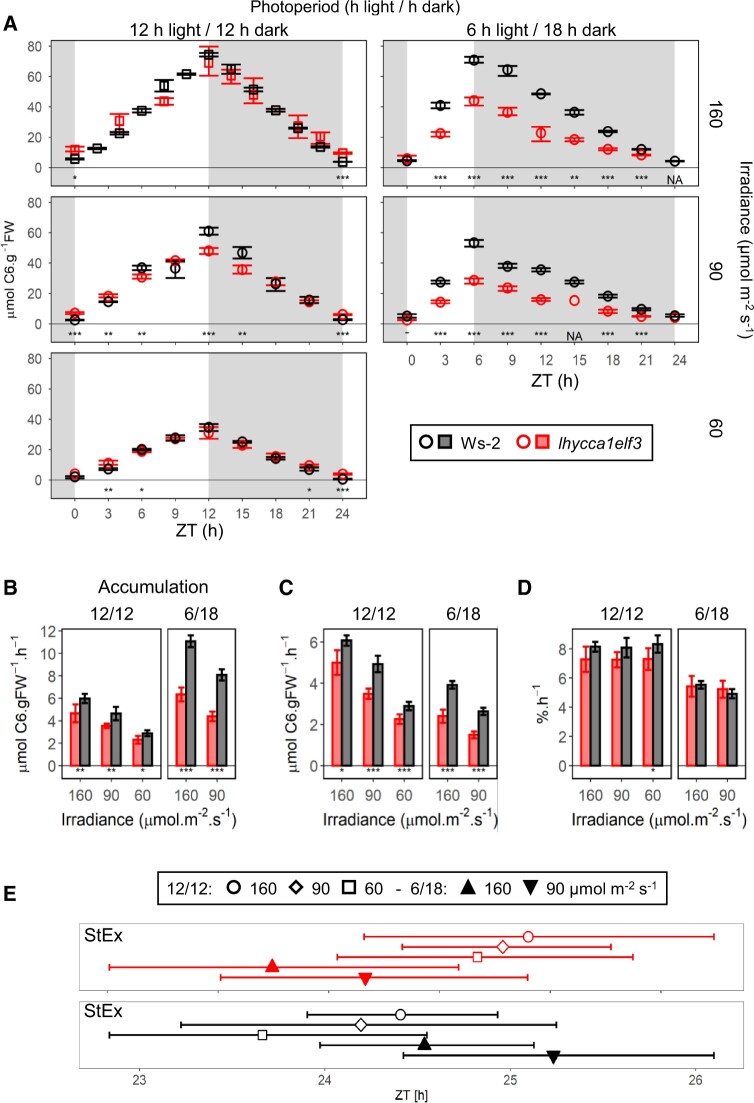
Diel starch turnover in plants growing in different photoperiods and irradiance. A, Diel changes in starch content. Plants were grown in a 12-h light/12-h dark (left-hand) or 6-h light/18-h dark (right-hand) photoperiod and irradiance of 160 µmol m^−2^ s^−1^ (top), 90 µmol m^−2^ s^−1^ (middle or 60 µmol m^−2^ s^−1^ (bottom). Harvest age and sampling strategy was designed to take account of the differing growth rates and rosette size in the various conditions. Plants were harvested over a full diel cycle at 13 DAS in the 12-h light/12-h dark at 160 µmol m^−2^ s^−1^ irradiance, 14 DAS in the 12-h light/12-h dark 90 and 60 µmol m^−2^ s^−1^ treatments and 16 DAS in all 6-h light/18-h dark treatments (see Supplemental Figure S1A). At each time point, two to five samples were harvested. Background shading indicates light period (white) and night (gray). Wild-type Ws-2 and *lhy cca1 elf3* are indicated by black and red symbols, respectively. The data for Ws-2 in a 12-h photoperiod at 160 µmol m^−2^ s^−1^ was collected in a separate experiment (Flis et al., 2015); otherwise all plants were grown in parallel (depicted as square and circular symbols, respectively). Symbols represent the mean value and error bars indicate the bootstrapped 95% CI. Statistical significance (Analysis of Variance [ANOVA], Sum of Squares type II) is indicated by asterisks (0 “***” 0.001 “**” 0.01 “*” 0.05; subsequent Honestly Significant Difference [HSD] Tukey’s post-hoc test was significant in all cases). NA denotes that a test was not applicable due to lack of replicates. “ZT,” or “Zeitgeber” from the German language, indicates the time elapsed after the last dawn, in hours. B, Estimated absolute rates of starch accumulation. C, Estimated absolute rates of starch mobilization. D, Estimated relative rates of starch mobilization. Ws-2 and *lhy cca1 elf3* are indicated by black and red, as in (A). Rates were defined as the slope of linear models, and error bars indicate the 95% CI of the standard error of the slope. Accumulation rates were calculated including all time points between dawn and dusk, and mobilization rates were calculated using time points between dusk and dawn. At each time point, two to five samples were harvested. Relative rates were calculated using starch levels as a proportion of the average starch levels at dusk in each condition. Statistical significance between wild-type Ws-2 and *lhy cca1 elf3* (Analysis of Covarian [ANCOVA], Sum of Squares type III) is indicated by asterisks (0 “***” 0.001 “**” 0.01 “*” 0.05). Numeric values are provided in Supplemental Data Set 1. E, Estimated time at which starch is exhausted. Symbols represent the projected time of StEx as defined by single extrapolation of the rate of starch mobilization (StEx). This was performed using time points between dusk and dawn. At each time point, two to five samples were harvested. Color indicates different genotypes, as in (A). Error bars indicate the standard error of the StEx estimation. Solid upward and downward triangles denote 90 and 160 µmol m^−2^ s^−1^ irradiance, respectively, in the 6 h/18 h and open squares, diamonds and circles denote 60, 90, and 160 µmol m^−2^ s^−1^ irradiance, respectively, in 12-h light/12-h dark photoperiod. Numeric values are provided in Supplemental Data Set 1. The time when starch would be exhausted as projected by nested linear fits is shown in Supplemental Figure S4B.

The triple *lhy cca1 elf3* mutant and wild-type Wassilewskija-2 (Ws-2) were grown in a 12-h photoperiod at 160 µmol m^−2^ s^−1^ (see Supplemental Figure 1A for experimental design); in this condition, wild-type plants are slightly source-limited (Sulpice et al., 2014). Compared to wild-type plants, the triple mutant showed only a small (10%–20%) decrease in biomass (Supplemental Figure S2A, see later for discussion of the other conditions). This resembles the biomass decrease in the parental *lhy cca1* and *elf3* mutants (Flis et al., 2019). Chlorophyll content was not significantly changed in the triple mutant (Supplemental Figure S2B). Strikingly, the pattern of diel starch turnover was almost identical in Ws-2 and *lhy cca1 elf3* (Figure 1A; upper left).

### Dynamics of circadian clock transcripts in a 12-h photoperiod

The *lhy cca1 elf3* mutant is deficient in dawn and evening clock components. To confirm that the residual transcriptional clock is severely disrupted in our growth regime, we re-investigated the temporal dynamics of transcript abundance for the genes included in the circadian clock model of Pokhilko et al. (2013). This was done using plant material from the experiment of Figure 1A (160 µmol m^−2^ s^−1^). The response (Supplemental Figure S3; left hand side) broadly recapitulated the results of Dixon et al. (2011). *PRR9* transcript oscillated, but with at least 2 times smaller amplitude and shifted peak time compared to Ws-2, *PRR5*, *TOC1*, *GI*, and *ELF4* transcripts showed a strongly attenuated oscillation with an advanced peak, and *PRR7* and *LUX* transcripts were high and almost constant. *LHY* and *CCA1* transcript abundance was extremely low. *ELF3* transcript, which in the mutant encodes a nonfunctional protein, was fairly constant.

We compared the transcript dynamics in *lhy cca1 elf3* with those of its parents (Supplemental Figure S3, central) growing in similar growth conditions (Flis et al., 2015, 2019; Mengin et al., 2017). Some features in *lhy cca1 elf3* resemble *lhy cca1*, in particular all remaining transcripts with an oscillation peaked early in the light period. This presumably reflects loss of the repressor function of the dawn components. However, except for *PRR9*, the amplitude of the oscillation was much smaller in *lhy cca1 elf3* than *lhy cca1.* Other features in *lhy cca1 elf3* resemble *elf3*, for example, in both mutants *PRR9* transcript started to rise well before the end of the night, and *PRR7, GI, TOC1 ELF4*, and *LUX* transcripts showed a strongly attenuated oscillation resulting in much higher abundance during the night and at dawn than in wild-type Ws-2. Published data for clock transcripts for the *prr7 prr9*, *gi*, and *toc1* mutants in a 12-h photoperiod (Flis et al., 2015) are summarized in Supplemental Figure S3 (right hand). They retained strong oscillations with large *(prr7 prr9*) or small (*gi, toc1*) changes in their timing. Summarizing, there is a far larger disruption of the transcriptional circadian clock in *lhy cca1 elf3* than in mutants previously investigated for diel starch turnover.

The wild-type-like pattern of diel starch turnover of *lhy cca1 elf3* in a 12-h photoperiod might be a default response in a nonchallenging condition, or might reflect an unexpected robustness of the network that delivers the temporal input. We therefore investigated diel starch turnover in Ws-2 and *lhy cca1 elf3* growing at lower irradiance or in a shorter photoperiod, as well as in non-T24 cycles. We also asked if *lhy cca1 elf3* can adjust starch mobilization to a sudden change in irradiance or the timing of dusk (Supplemental Data Set 1).

### Diel starch turnover in different growth irradiances and photoperiods

Ws-2 and *lhy cca1 elf3* were investigated in a 12-h photoperiod at 90 and 60 µmol m^−2^ s^−1^ and in a 6-h photoperiod at 160 and 90 µmol m^−2^ s^−1^ irradiance. For low irradiance treatments, plants were grown initially at a higher irradiance and transferred to lower irradiance at least 3 days before the start of the experiment (Supplemental Figure S1A). Compared to wild-type Ws-2, *lhy cca1 elf3* contained less ChlA, unaltered or marginally higher ChlB and a lower ChlA:ChlB ratio than Ws-2 in these low irradiance or short photoperiod conditions (Supplemental Figure S2B).

As previously reported (see “Introduction”) Ws-2 accumulated starch more rapidly in short photoperiods than in long photoperiods (Figure 1A) and always mobilized starch in a near-linear manner at a rate that almost exhausted starch at dawn (Figure 1A).*lhy cca1 elf3* was compromised in its ability to speed up starch accumulation in short photoperiods, compared to long photoperiods (Figure 1A). The rate of accumulation was estimated as the slope of a linear model fitted to all data points between ZT0 (“ZT,” or “Zeitgeber” from the German language, indicates the time elapsed after the last dawn, in hours) and the end of the light period. Compared to wild-type plants, starch accumulation in the triple mutant was slightly but significantly slower in the three 12-h photoperiod treatments and almost halved in the 6-h photoperiod treatments (Figure 1B). Whereas starch accumulation increased strongly and significantly between a 12-h and a 6-h photoperiod in Ws-2 (by 85% and 74% when grown at 160 and 90 µmol m^−2^ s^−1^, respectively; the 95% confidence interval (CI) do not overlap between photoperiods), the response was much weaker in *lhy cca1 elf3* (36% and 23% at 160 and 90 µmol m^−2^ s^−1^, in both cases the 95% CI overlap).

Growth of *lhy cca1 elf3* in lower irradiance or a shorter photoperiod led to a slowing down of starch mobilization (Figure 1C) with the result that starch was exhausted at around dawn in all growth conditions. Close inspection reveals that dawn starch content in the triple mutant was slightly but significantly higher than in Ws-2 in a 12-h photoperiod and slightly but nonsignificantly lower than Ws-2 in a 6-h photoperiod (Figure 1A).

Comparison of the temporal kinetics of starch mobilization in Ws-2 and *lhy cca1 elf3* in a 6-h photoperiod is complicated by their differing dusk starch content. We, therefore, replotted the data after normalizing on dusk starch content (Supplemental Figure S4A). The relative rate of starch mobilization (percentage dusk starch level per hour) was estimated as the slope of a linear model fitted to all normalized time points in the night (Figure 1D). The relative rate of mobilization in the triple mutant resembled Ws-2 in a 6-h photoperiod and was marginally slower than Ws-2 in a 12-h photoperiod (significant at 60 but not 90 or 160 µmol m^−2^ s^−1^ irradiance).

The slope of the linear fits that were used to estimate mobilization rates in Figure 1, C and D were extrapolated to identify when they would lead to starch being exhausted. The time elapsing (in hours) between the previous dawn and the estimated time of starch exhaustion was termed StEx (Figure 1E). Estimated values of StEx in wild-type plants at 160, 90, and 60 µmol m^−2^ s^−1^ irradiance in a 12-h photoperiod, and 160 and 90 µmol m^−2^ s^−1^ irradiance in the 6-h photoperiod were 24.4, 24.2, 23.7, 24.5, and 25.2 h, respectively. The corresponding values in the triple mutant were 25.8, 25.6, 25.3, 23.5, and 24.3 h. Thus, compared to wild-type plants, in the triple mutant the estimated time of StEx is slightly delayed in a 12-h photoperiod (by ∼1.5 h) and slightly advanced in a 6-h photoperiod (by ∼1 h). Incidentally, the values for Ws-2 in this study resemble earlier studies with Col-0 or Ws-2 growing in a 12-h photoperiod at 160 µmol m^−2^ s^−1^ irradiance (24.3, 24.2, and 24.4 h; Mengin et al., 2017; Flis et al., 2019; Moraes et al., 2019).

Summarizing, *lhy cca1 elf3* is significantly compromised in its ability to increase starch accumulation in a short compared to a long photoperiod. However, it is only slightly impaired in its ability to pace mobilization to dawn over a range of growth irradiances and photoperiods. This contrasts with its parents, *lhy cca1* and *elf3*. Their diel starch turnover deviates from a 24-h rhythmicity (see “Introduction”) with starch being exhausted before dawn in *lhy cca1* and being incompletely mobilized at dawn in *elf3* (Graf et al., 2010; Flis et al., 2019).

### Starch turnover in non-T24 cycles

*lhy cca1 elf3* might pace starch mobilization to dawn using an internal 24-h rhythm, or by sensing the duration of the external light–dark cycle. To distinguish between these possibilities, we grew Ws-2 and the triple mutant in an 8.5-h light/8.5-h dark (T17) cycle, or a 14-h light/14-h dark (T28) cycle (for details, see Supplemental Figure S1B).

**Figure 2:**
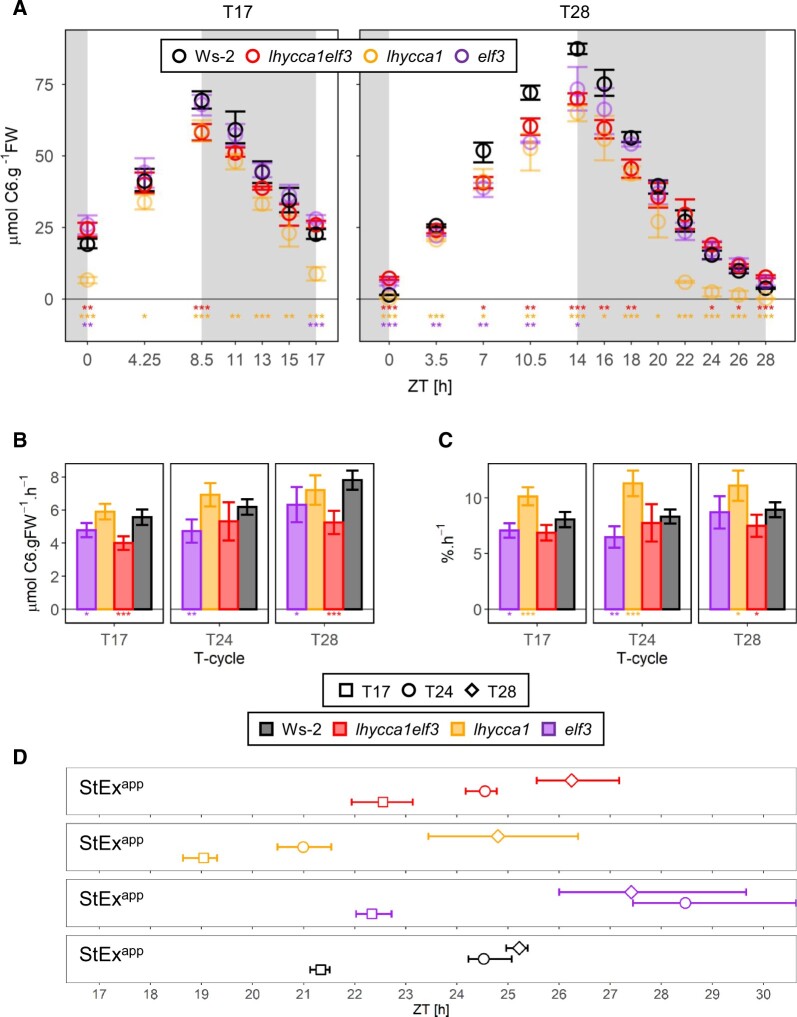
Diel starch turnover in non-T24 cycles. A, Diel changes in starch content. Plants were grown in 8.5-h light/8.5-h dark cycle (T17, left hand) or 14-h light/14-h dark cycle (T28, right hand) at 160 µmol m^−2^.s^−1^. Plants were harvested over a full diel cycle, 13 diel cycles after sowing. At each time point, two to five samples were harvested. Background shading indicates light period and night. Wild-type Ws-2, *lhy cca1 elf3*, *lhy cca1*, and *elf3* are indicated by black, red, orange, and purple symbols, respectively (see insert). Symbols represent the mean and error bars indicate the bootstrapped 95% CI. Statistical significance (ANOVA, sum of squares type II) is indicated by asterisks (0 “***” 0.001 “**” 0.01 “*” 0.05; subsequent HSD Tukey’s post-hoc test was significant in all cases) for each wild-type—mutant comparison and is denoted by the color code for that mutant. “ZT,” or “Zeitgeber” from the German language, indicates the time elapsed after the last dawn, in hours. B, Estimated absolute rates of starch mobilization. C, Estimated relative rates of starch mobilization. Plants grown in T17 and T28 conditions were compared to plants grown in 24-h diel cycle conditions (T24). Ws-2, *lhy cca1 elf3*, *lhy cca1*, and *elf3* are indicated by different colored symbols, as in (A). Rates were defined as the slope of linear models, and error bars indicate the 95% CI of the standard error of the slope. These were performed excluding times beyond 8.5 h of darkness (highlighted in dotted box in Supplemental Figure S5A). This allowed comparison of T-cycles over a similar duration after dusk. Relative rates were calculated using starch levels as a proportion of the average starch levels at dusk in each condition. At each time point, two to five samples were harvested. Statistical significance between wild-type Ws-2 and *lhy cca1 elf3* (ANCOVA, Sum of Squares type III) is indicated by asterisks (0 “***” 0.001 “**” 0.01 “*” 0.05). Numeric values are provided in Supplemental Data Set 1. D, Estimated time at which starch is exhausted in T17, T24, and T28 cycles. Symbols represent the projected time of StEx as defined by the geometric mean of multiple projections (StEx^app^). These were performed using linear models on time spans of varying lengths that started at dusk and extended for increasing lengths of time into the night (at least three time points) excluding times beyond 8.5 h of darkness (as in (B and C), highlighted in dotted box in Supplemental Figure S5A). This allowed comparison of T-cycles over a similar duration after dusk. Error bars indicate the 95% CI of the multiple estimations. At each time point, 2–5 samples were harvested. Solid and open symbols indicate results from 6-h/18-h to 12-h/12-h light–dark cycle conditions, respectively. Color indicates different genotypes, as in (A). Numeric values are provided in Supplemental Data Set 1.

As previously observed (see “Introduction”), wild-type plants set a rate of mobilization that does not completely exhaust starch at dawn in a T17 cycle, and that exhausts starch before dawn in a T28 cycle (Figure 2A). Whilst the response of Ws-2 in the T28 cycle in Figure 2A qualitatively resembles that of wild-type plants in Graf et al. (2010), there is a more marked slowing of mobilization from about ZT22 than in the earlier study. This might reflect differences in plant age, small differences in growth conditions or the use of a different accession (Ws-2 in this study, Col-0 and C-24 in Graf et al. (2010). It might also be noted that if starch mobilization is paced to about 24 h after dusk, there might be a hiatus in a T28 cycle as the plant approaches 24 h after the previous dusk, because the internal cycle would be signaling that the plant is entering a new 24 h cycle, possibly leading to a slowing of the degradation of any starch remaining at this time. The *lhy cca1 elf3* triple mutant largely resembled wild-type Ws-2; it retained considerable amounts of starch at dawn in a T17 cycle and set an initial rate that would exhaust its starch before dawn in a T28 cycle (to aid visual comparison in different T-cycles, in Supplemental Figure S5A starch content is replotted after normalization on dusk starch content). The absolute rate of starch mobilization in *lhy cca1 elf3* was lower than that in Ws-2, both in the T17 and T28 cycle (Figure 2B). However, as already seen in a T24 cycle, this was mainly due to the lower dusk starch content in *lhy cca1 elf3.* When rates were calculated after normalization on dusk starch content, compared to Ws-2, the normalized rate in *lhy cca1 elf3* was not significantly different in a T17 cycle and only slightly slower in the T28 cycle (Figure 2C).

For comparison, we investigated starch turnover in different T-cycles in *lhy cca1* and *elf3*. As previously reported, starch was exhausted before dawn in *lhy cca1* and incompletely mobilized at dawn in *elf3* in a T24 cycle (Supplemental Figure S5A, central). At a qualitative level, these deviations from 24-h rhythmicity were retained in non-T24 cycles. In a T17 cycle, *lhy cca1* largely exhausted its starch and *elf3* showed a larger excess of starch at dawn than Ws-2 (Figure 2A, left hand). In a T28 cycle, *lhy cca1* mobilized its starch faster and *elf3* mobilized its starch slightly more slowly than wild-type Ws-2 (Figure 2A, right hand). Estimation of the absolute and relative rates of starch mobilization (Figure 2, B and C) confirmed a trend to faster starch mobilization in *lhy cca1* (especially when estimated on a relative basis) and slower starch mobilization in *elf3*.

We analyzed these time series to estimate when starch would be exhausted in T17, T24, and T28 cycles (Figure 2D). The estimates for T24 used data from Figure 1A (upper left) for *lhy cca1 elf3* and wild-type Ws-2 and published data (Flis et al., 2019) for *lhy cca1* and *elf3*. Two factors in the data set from the T-cycle experiments complicated this analysis: first, the short period mutant *lhy cca1* exhausts its starch prematurely in T24 and T28 cycles and, second, starch mobilization slows down in Ws-2, *elf3*, and *lhy cca1 elf3* from about ZT22 onwards in a T28 cycle (see above). To allow comparison of the kinetics of mobilization across all genotypes and T-cycles, we restricted our analysis to a time range that corresponded to the duration of the night in a T17 cycle (i.e. dusk and the first 8.5 h of darkness). This time range, which corresponded to the first 71% and 61% of the night a T24 and T28 cycles, respectively, provided a good estimate of the initial rate of mobilization set after dusk. Multiple nested linear fits on starch content in this time range were extrapolated to zero starch content to estimate the apparent StEx (StEx*^app^;* see “Materials and method” below for more details). Figure 2D shows the geometric mean and 95% CI limits for all estimates of StEx^*app*^ (the time points used in the linear fits are indicated by the blue dashed boxes in Supplemental Figure S5A).

In wild-type plants, projected values of StEx^*app*^ in a T17, T24, and T28 cycle were 21.3, 24.5, and 25.2 h, respectively, all with tight CI limits (Figure 2D). Thus, in wild-type plants a circa 24-h rhythmicity of starch turnover is large although not completely retained when the external light–dark cycle deviates from 24 h. In *lhy cca1 elf3*, projected values of StEx^*app*^ in a T17, T24, and T28 cycle were 22.6, 24.6, and 26.2 h, respectively, which does not differ greatly from Ws-2. In the *lhy cca1* double and *elf3* single mutants, rhythmicity of starch mobilization was less robust against non-T24 cycles. Projected values for StEx^*app*^ in T17, T24, and T28 of 19.0, 21.0, and 24.8 h for *lhy cca1* and 22.3, 28.5, and 27.4 h for *elf3*, respectively. For *lhy cca1* the StEx^*app*^ values in T17 and T24 values are quite close to its short internal period, whilst the T28 value is considerably longer. For *elf3*, the T24 and T28 values qualitatively match its apparent long internal period whilst the T17 value is considerably shorter. It is noteworthy that StEx^*app*^ for *lhy cca1* in a T28 cycle and StEx^*app*^ for *elf3* in a T17 cycle resemble the corresponding values for wild-type Ws-2 (24.8 versus 25.2, and 22.3 versus 21.3, respectively).

The stringency of the 24-h rhythmicity of starch turnover can also be gaged by comparing the magnitude of the increase in StEx^*app*^ between the T17 cycle and the T28 cycle with the 11-h shift in the duration of the external light–dark cycle. StEx^*app*^ increases by 3.8 h in wild-type Ws-2 and 3.6 h in *lhy cca1 elf3*, compared to 5.8 h in *lhy cca1* and 5.1 h in *elf3*.

We checked that the StEx^*app*^ values projected by multiple nested estimations resembled StEx values obtained by regression across all time points in the night (i.e. estimation of the measured total rates). There was good agreement for Ws-2 under different irradiance in a 12-h light/12-h dark cycle and a 6-h light/18-h dark (compare Supplemental Figure S4B and Figure 1E). There was also good agreement between StEx^*app*^ and StEX for all three irradiancies in a 12-h light/12-h dark cycle for *lhy cca1 elf3* (compare Supplemental Figure S4B and Figure 1E), but projected values for StEx^*app*^ were about 2 h earlier compared to estimates of StEx when *lhy cca1 elf3* was in an 8-h light/16-h dark cycle (see below for more discussion).

Together, these analyses show that *lhy cca1 elf3* maintains a near-24-h rhythmicity for starch turnover in non-T24 cycles. It maintains 24-h rhythmicity as robustly as wild-type Ws-2 and more robustly than its parents.

### Response of starch mobilization to a sudden perturbation of the conditions

We next asked if *lhy cca1 elf3* is able to adjust the rate of starch mobilization after a sudden change in conditions. To do this we grew *lhy cca1 elf3* in a 12-h photoperiod at 160 µmol m^−2^ s^−1^ and then subjected it, on the harvest day, to lower irradiance for one light period or to a sudden early dusk (see Supplemental Figure S1, C and D for experimental details).

**Figure 3:**
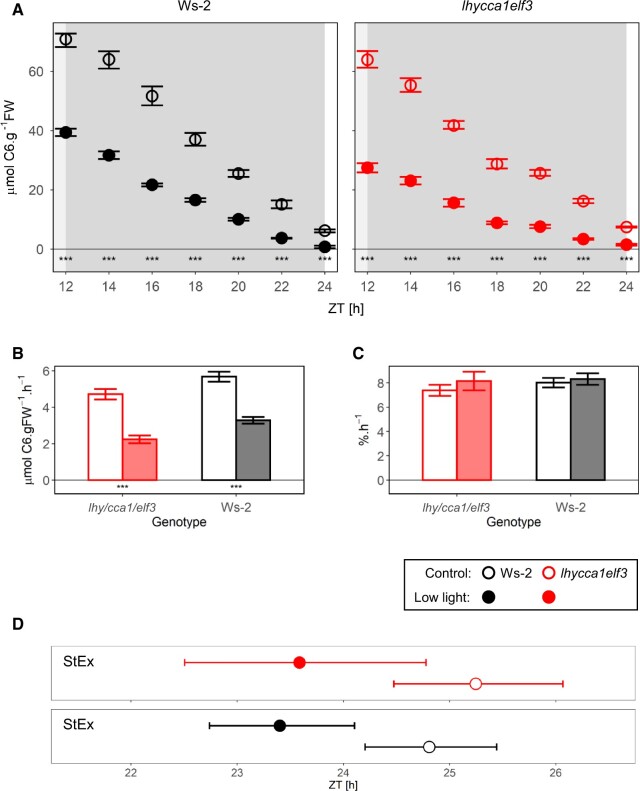
Starch degradation after a single day of low irradiance. Wild-type Ws-2 (left hand, black symbols) and *lhy cca1 elf3* (right hand, red symbols) were grown in 12-h light/12-h dark at 160 µmol m^−2^ s^−1^ for 13 days and then harvested at different times during the night following a day at growth irradiance (open symbols) or a day on which the irradiance was decreased to 90 µmol m^−2^ s^−1^ from dawn on (solid symbols). At each time point, two to five samples were harvested. A, Starch content. Wild-type Ws-2 (left hand, back symbols) and *lhy cca1 elf3* (right hand, red symbols) were grown in 12-h light/12-h dark at 160 µmol m^−2^ s^−1^ for 13 days and then harvested at different times during the night following a day at growth irradiance (open symbols) or a day on which the irradiance was decreased to 90 µmol m^−2^ s^−1^ from dawn on (solid symbols). At each time point, two to five samples were harvested. Background shading indicates light period (white) and night (gray). Wild-type Ws-2 and *lhy cca1 elf3* are indicated by black and red symbols, respectively. Symbols represent the mean values, and the error bars indicate bootstrapped 95% CI. Statistical significance (ANOVA, Sum of Squares type II) is indicated by asterisks (0 “***” 0.001 “**” 0.01 “*” 0.05; subsequent HSD Tukey’s post-hoc test was significant in all cases). “ZT,” or “Zeitgeber” from the German language, indicates the time elapsed after the last dawn, in hours. B, Estimated absolute rates of starch mobilization. C, Estimated relative rates of starch mobilization. Ws-2 and *lhy cca1 elf3* are indicated by black and red symbols, as in (A). Rates were defined as the slope of linear models, and error bars indicate the 95% CI of the standard error of the slope. Mobilization rates were calculated using time points between dusk and dawn. At each time point, two to five samples were harvested. Relative rates were calculated using starch levels as a proportion of the average starch levels at dusk in each condition. Statistical significance between wild-type Ws-2 and *lhy cca1 elf3* (ANCOVA, Sum of Squares type III) is indicated by asterisks (0 “***” 0.001 “**” 0.01 “*” 0.05). Numeric values are provided in Supplemental Data Set 1. D, Estimated time at which starch is exhausted. Symbols represent the projected time of StEx as defined by single extrapolation of the rate of starch mobilization (StEx). This was performed using time points between dusk and dawn. At each time point, two to five samples were harvested. Error bars indicate the standard error of the StEx estimation. Solid and open symbols indicate results from “Low light” and “Control” conditions, respectively. Color indicates different genotypes, as in (A). Numeric values are provided in Supplemental Data Set 1. The time when starch would be exhausted as projected by nested linear fits is shown in Supplemental Figure S6B).

**Figure 4:**
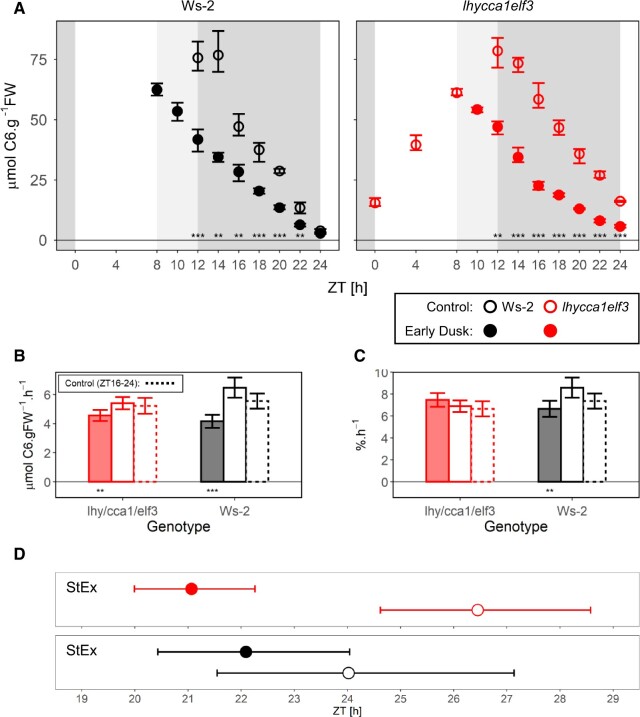
Starch degradation after a sudden early dusk. The *lhy cca1 elf3* triple mutant was grown in 12-h light/12-h dark at 160 µmol m^−2^ s^−1^ for 13 days and then harvested at different times during the night following a day in the growth regime (open symbols) or after a sudden early dusk at ZT8 (solid symbols; “ZT,” or “Zeitgeber” from the German language, indicates the time elapsed after the last dawn, in hours). At each time point, two to seven samples were harvested. Three further experiments are shown in Supplemental Figure S7. The data for Ws-2 in a 12-h photoperiod at 160 µmol m^−2^ s^−1^ was collected in a separate experiment (Flis et al., 2019). A, Starch content. Background shading indicates light period (white), the time during which some plants were in the light and others had been already darkened (pale gray), and the time when all plants were in darkness (gray). Symbols represent the mean values and the error bars indicate bootstrapped 95% CI. Statistical significance (ANOVA, Sum of Squares type II) is indicated by asterisks (0 “***” 0.001 “**” 0.01 “*” 0.05); subsequent HSD Tukey’s post-hoc test was significant in all cases). B, Estimated absolute rates of starch mobilization. C, Estimated relative rates of starch mobilization. Ws-2 and *lhy cca1 elf3* are indicated by black and red symbols, as in (A). Rates were defined as the slope of linear models, and error bars indicate the 95% CI of the standard error of the slope. Mobilization rates were calculated using time points between ZT12 and ZT24 in the control condition and from ZT8 to ZT18 in the early dusk treatment (solid bars). At each time point, 2–7 samples were harvested. Solid bars represent the treatment condition whilst the hollow bars indicate control conditions. Dashed line bars indicate results in control conditions but restricted from ZT16 to ZT24. Relative rates were calculated using starch levels as a proportion of the average starch levels at dusk in each condition. Statistical significance between wild-type Ws-2 and *lhy cca1 elf3* (ANCOVA, Sum of Squares type III) is indicated by asterisks (0 “***” 0.001 “**” 0.01 “*” 0.05). Numeric values are provided in Supplemental Data Set 1. D, Estimated time at which starch is exhausted. Symbols represent the projected time of StEx as defined by single extrapolation of the rate of starch mobilization (StEx). This was performed excluding times beyond 8.5 h of darkness, corresponding to ZT20 and ZT16 in the “Control” and the “Early Dusk” conditions, respectively. This allowed comparison over a similar duration after dusk. Error bars indicate the standard error of the StEx estimation. At each time point, two to seven samples were harvested. Solid and open symbols indicate results from “Early Dusk” and “Control” conditions, respectively. Color indicates different genotypes, as in (A). Numeric values are provided in Supplemental Data Set 1. The time when starch would be exhausted as projected by nested linear fits is shown in Supplemental Figure S7F). Numeric values are provided in Supplemental Data Set 1.

Compared to the control (160 µmol m^−2^ s^−1^), reducing irradiance to 90 µmol m^−2^ s^−1^ for one day resulted in a 42% decrease in dusk starch content in Ws-2 and a slightly larger 52% decrease in *lhy cca1 elf3* (Figure 3A). After darkening, both genotypes degraded their starch more slowly than in the control treatment (Figure 3B). To aid visual inspection, starch levels were normalized on the dusk starch content in a given genotype and treatment (Supplemental Figure S6A). In both genotypes, the relative rate of starch mobilization (percentage dusk starch level per hour) was very similar in the control and low irradiance treatments (Figure 3C). Close visual inspection of the normalized plots indicates that in both genotypes the initial relative rate of degradation may be slightly faster after a sudden low irradiance day than in stable high irradiance (see below for further analysis). Correspondingly, compared to the control the estimated values of StEx (Figure 3D) and StEx^app^ (Supplemental Figure S6B) were about 1.5–2 h earlier in the low irradiance in both Ws-2 and *lhy cca1 elf3*.*lhy cca1 elf3* was subjected to a 4-h early dusk in four separate experiments (Figure 4; Supplemental Figure S7). In one experiment, older *lhy cca1 elf3* plants were used to allow comparison with similarly sized wild-type plants. Visual inspection indicated that starch mobilization slowed down compared to plants that were left in growth conditions. Estimation of the extent of slowdown is complicated by the nonlinear decline in starch content in the later part of the night after an early dusk. The rate of mobilization in the controls was estimated using either all time points (ZT12–ZT24) or time points where starch content was below that at the time at which the treated plants were suddenly darkened (ZT16–ZT24). The initial rate in the early dusk treatment was calculated excluding time points when starch was lower than at dawn in the control treatment (i.e. excluding time points after about ZT18). In the four experiments, the estimated decrease in mobilization rate was ∼15% and 12% (experiment of Figure 4), 24% and 22%, and 25% and 20% (Experiments 3 and 4 of Supplemental Figure S7, respectively). For plants growing in a 12-h photoperiod, advancing dusk by 4 h will decrease dusk starch content by about 33% (starch accumulation is largely linear with time) and lengthen the night by 33%, meaning that a 50% decrease in the rate of starch mobilization would be required to exhaust starch at the same time as in nonperturbed plants. In published experiments, wild-type plants decreased the rate of starch mobilization by ∼47% (Graf et al., 2010), 35% (Scialdone et al., 2013), and 40%–42% (Flis et al., 2019) when dusk was advanced by 4 h. Several other clock mutants achieved a slowing of 18%–42% when dusk was advanced by 4 h (Flis et al., 2019). *lhy cca1* decreased the rate of starch mobilization by ∼30% when dusk was advanced by 3 h (Scialdone et al., 2013). The response of *lhy cca1 elf3* to an early dusk is weaker than in wild-type plants, but lies in the same range as that of mutants with a less severely disrupted clock. This weakening of the response to an early dusk is also indicated by inspection of the relative rates of mobilization (Figure 4C); whereas this was significantly decreased after an early dusk in wild-type Ws-2 it was not decreased in *lhy cca1 elf3*. Estimation of StEx (Figure 4D) and StEx^app^ (Supplemental Figure S7F) indicated a trend in which starch would be exhausted slightly earlier after a sudden early dusk than in the control, with the estimated advance in *lhy cca1 elf3* often being larger than in Ws-2.

Taken together, these results show that *lhy cca1 elf3* is able to integrate information about unanticipated changes in the amount of starch at dusk and the duration of the night although, in the latter case, less effectively than wild-type plants.

### Linearity of starch mobilization

**Figure 5:**
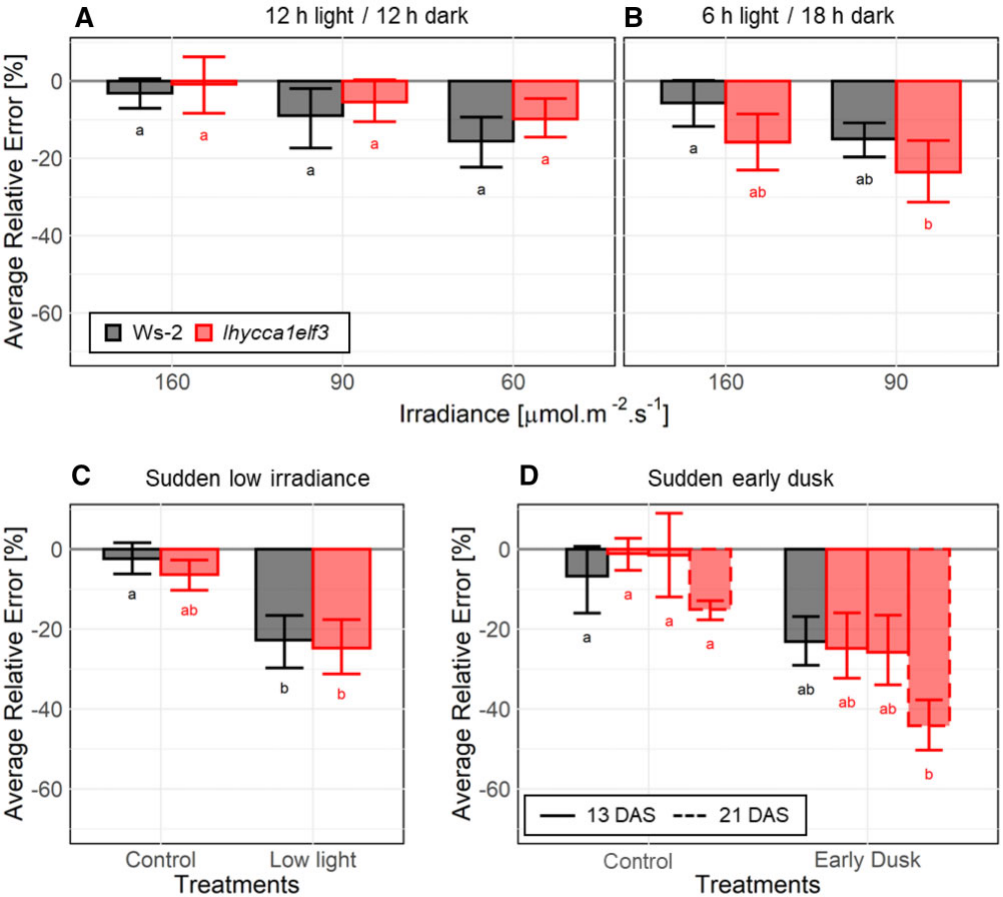
Analysis of the extent to which starch mobilization is nonlinear in stable diurnal cycles, and after a sudden low light day or a sudden early dusk. If starch mobilization is linear, the content at different times in the night will lie on a straight line between dusk starch content and dawn starch content. To score the deviation of starch mobilization from linearity, at each time point the starch content expected if mobilization were linear was subtracted from the measured starch content, the difference (residue) was normalized on the content expected if mobilization was linear, and the normalized residues were averaged across all time points to give an average relative error, or score for nonlinearity. This score is zero if breakdown is linear, and close to zero if noise leads to small random deviation from linearity. A consistent deviation from linearity will lead to a negative score if mobilization is initially too fast, and a positive score if mobilization is initially too slow. A, 12-h light/12-h dark with 160 or 90 µmol m^−2^ s^−1^ irradiance (data from Figure 1). B, 6-h light/18-h dark photoperiod with 160 or 90 µmol m^−2^ s^−1^ irradiance (data from Figure 1). C, In the night after a sudden drop in irradiance from growth conditions (12-h light 12-h dark with 160 µmol m^−2^ s^−1^ (control) to 90 µmol m^−2^ s^−1^ (sudden low light) (data from Figure 3). D, In the night after a sudden 4-h advance in dusk (sudden early dusk) compared to growth conditions (12-h light 12-h dark with 160 µmol m^−2^ s^−1^, control). Ws-2 data are from Flis et al. (2019). Data for *lhy cca1 elf3* are from Figure 4 and Supplemental Figure S7, Experiments #27, #4, and #3 (13 DAS) are shown from left to right; the analysis was performed for Experiment #11 despite the low density of sampling (4 h instead of 2 h intervals; at 21 DAS). The plots show the mean and 95% CI (calculated using a nonparametric bootstrap procedure) of the nonlinearity score. At each time point, two to five samples were harvested in the sudden “low light” experiment and two to seven in the “sudden early dusk” experiment. Significant statistical differences (indicated by letters; at 95% CI level, *P* ≤ 0.5) between groups of results in each part was analyzed using ANOVA and subsequent HSD Tukey’s post-hoc test (significant in all cases).

As already mentioned, visual inspection of Figures 1–4 and Supplemental Figures S4–S7 indicated that starch mobilization was sometimes less linear in *lhy cca1 elf3* than in wild-type Ws-2. There is also some variability in the linearity of degradation in wild-type plants and mutants in various published studies (Graf et al., 2010; Mugford et al., 2014; Flis et al., 2019; Moraes et al., 2019). We used the following approach to score nonlinearity across treatments and genotypes in our study. The underlying rationale was that deviation from linearity in mobilization will lead to consistent displacement of measured starch values above or below a straight line between the initial and final value (i.e. the content at dusk and dawn, respectively), whereas noise will lead to some values lying above and some below the line. We estimated the deviation from linearity at each time point, normalized it on the content that would result if mobilization were ideally linear, and averaged the normalized values across the whole night (Figure 5, nonzero values indicate when there is deviation from linearity, error bars represent the 95% CI; see legend for details of the calculation).

Starch breakdown was strictly linear in a 12-h photoperiod at an irradiance of 160 µmol m^−2^ s^−1^, both in wild-type Ws-2 and in *lhy cca1 elf3* (Figure 5A). It deviated from linearity when plants were grown at lower irradiance and, even more so, when they were grown in a 6-h photoperiod (Figure 5, A and B). The deviation was consistent though nonsignificantly larger for Ws-2 than *lhy cca1 elf3* in a 12-h photoperiod and was larger for *lhy cca1 elf3* than Ws-2 in a 6-h photoperiod. When plants were grown in a 12-h photoperiod and irradiance was suddenly decreased from 160 to 90 µmol m^−2^ s^−1^, the deviation from linearity increased to a similar extent in Ws-2 and *lhy cca1 elf3* (Figure 5C; significant for Ws-2). For both genotypes, the deviation from linearity was larger after a sudden decrease to 90 µmol m^−2^ s^−1^ than when plants were grown at 90 µmol m^−2^ s^−1^ (compare Figure 5, C with Figure 5, A). When plants were grown in a 12-h photoperiod at 160 µmol m^−2^ s^−1^ and subjected to a sudden dusk at ZT8, there was a deviation from linearity in Ws-2 and a slightly larger deviation in *lhy cca1 elf3* (Figure 5D). The deviation was larger than when plants were grown in a 6-h photoperiod (compare Figure 5, D with Figure 5, B).

A similar approach was taken for non-T24 cycles. In a T17 cycle, mobilization was highly linear. In a T24 cycle mobilization was linear in wild-type Ws-2, *lhy cca1 elf3* and *elf3*, but deviated strongly from linearity in *lhy cca1*, due to premature exhaustion of starch in the latter. In a T28 cycle mobilization deviated significantly from linearity in wild-type Ws-2, *lhy cca1 elf3* and *elf3* and even more strongly in *lhy cca1* (Supplemental Figure S5B).

Summarizing, starch mobilization was strictly linear when plants were grown in a 12-h photoperiod with moderately high light, deviated marginally from linearity when they were grown in low irradiance or a shorter photoperiod, and showed larger deviations when irradiance was suddenly decreased or dusk is advanced, or when plants were grown in a long T-cycle. Compared to wild-type Ws-2, *lhy cca1 elf3* retained linearity as well as wild-type plants in a 12-h photoperiod and non-T24 cycles but showed a slightly larger deviation from linearity in short photoperiods.

### Levels of sugars

To learn if the relationship between starch turnover and sugar metabolism is modified in *lhy cca1 elf3*, we analyzed glucose, fructose, and sucrose in the experiments of Figures 1–4. Sugars rise in the light period and decrease at night in both genotypes (Supplemental Figure S8, data provided in Supplemental Data file S1). In the light, sugars tended to be higher in *lhy cca1 elf3* than Ws-2, with the changes being often significant for glucose and fructose (Supplemental Figure S8, A and B), and sometimes significant for sucrose (Supplemental Figure S8C). This broadly recapitulates the response of sugars in the *elf3* mutant (Flis et al., 2019). At night, glucose and fructose were significantly lower at some time points in the triple mutant than wild-type plants, especially in a 6-h photoperiod, a T28 cycle or after a low irradiance day (Supplemental Figure S8, A and B). In the 6-h photoperiod, the lower sugar levels at night may reflect the lower dusk starch content and lower absolute rate of starch mobilization in *lhy cca1 elf3*, compared to wild-type plants. When *lhy cca1 elf3* was subjected to an early dusk at ZT8, glucose, fructose, and sucrose levels fell immediately after darkening, from ZT12 onwards resembled those in control plants that were darkened at ZT12, and fell to lower levels than the control in the last part of the night after starch was partly exhausted and the rate of mobilization declined.

### C starvation marker transcripts

**Figure 6:**
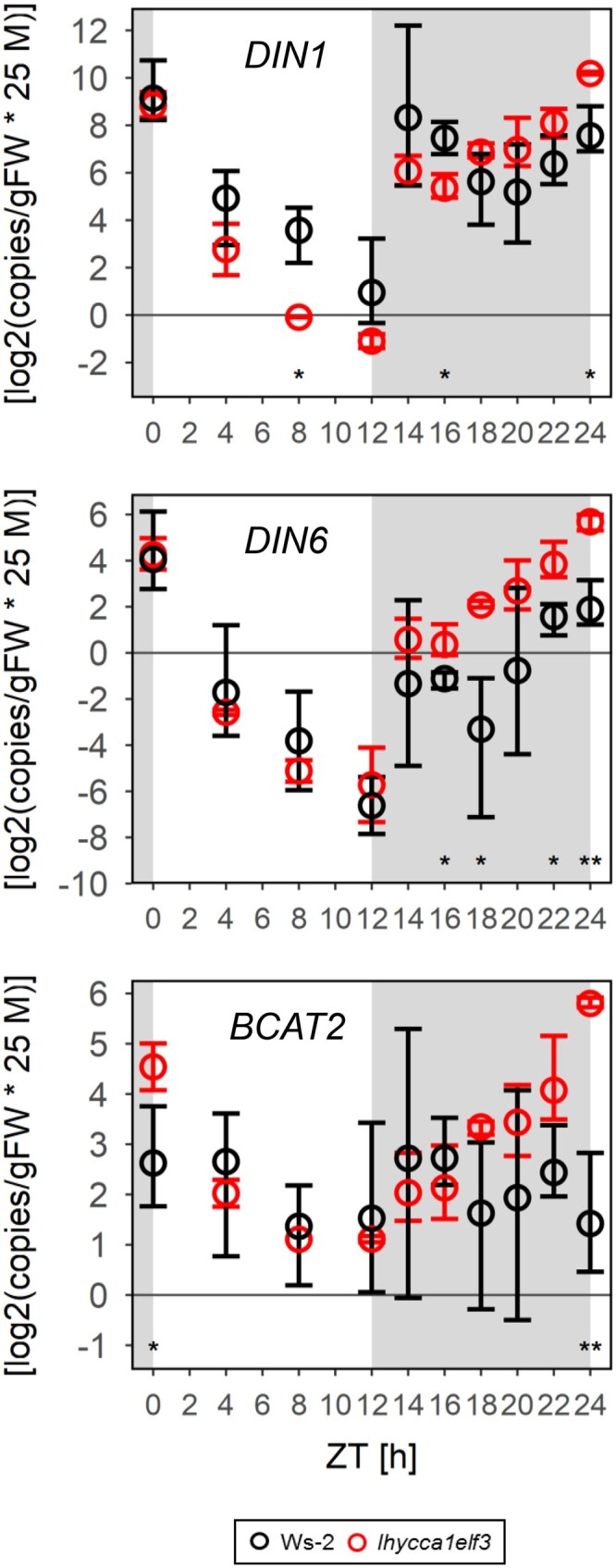
Response of C-starvation reporter transcripts in 12-h photoperiod. The C starvation reporter transcripts *DIN1*, *DIN6*, and *BCAT2* were measured in the 12-h photoperiod at 160 µmol m^−2^ s^−1^ irradiance experiment of Supplemental Figure S1A (the experiment used to measure starch and sugars in this condition, see Figure 1; Supplemental Figure S8). At each time point, two to five samples were harvested. Ws-2 data from Flis et al. (2019). Transcript abundance was measured by quantitative reverse transcription polymerase chain reaction (RT-qPCR), adding artificial RNA standard before cDNA amplification to allow absolute quantification. Abundance is given as log_2_(copies × 2.5 × 10^7^ g^−1^ FW). Background shading indicates the light period (white) or night (gray). Wild-type Ws-2 and *lhy cca1 elf3* are shown as black and red symbols, respectively (see insert). The symbols give the mean value, and error bars indicate the bootstrapped 95% CI. Statistical significance (ANOVA, Sum of Squares type II) is indicated by asterisks (0 “***” 0.001 “**” 0.01 “*” 0.05, subsequent HSD Tukey’s post-hoc test (significant in all cases). “ZT,” or “Zeitgeber” from the German language, indicates the time elapsed after the last dawn, in hours.

We also investigated the responses of three C-starvation reporter genes in a standard 12-h photoperiod (Figure 6). In the light, *DARK INDUCIBLE1 (DIN1)* but not *DIN6* transcript was lower in *lhy cca1 elf3* than wild-type Ws-2. *DIN1* and *DIN6* transcript rose in the first 2 h of the night, reaching similar values in both genotypes. This may reflect transient C depletion, because there is a short delay until starch degradation starts after sudden darkening (Pal et al., 2013; Annunziata et al., 2017). *DIN1* and *DIN6* transcripts subsequently declined and rose again at the end of the night in Ws-2, whereas they remained high in *lhy cca1 elf3*. *BRANCHED-CHAIN AMINO ACID TRANSAMINASE2* (*BCAT2*) transcript levels were low throughout the 24-h cycle in wild-type Ws-2, but rose in the second part of the night in *lhy cca1 elf3*. These results indicate that C-starvation signaling is more strongly activated at night in *lhy cca1 elf3* than wild-type Ws-2, in agreement with the slightly lower sugar levels at night in *lhy cca1 elf3* than Ws-2.

Taken together, these analyses show that *lhy cca1 elf3* has similar or higher levels of sugars than Ws-2 in the light, indicating that its low rate of starch accumulation in short photoperiods is not an indirect effect due to allocation of more C to growth. They also show *lhy cca1 elf3* paces mobilization to dawn even though its night-time sugar levels are often lower and C starvation reporter transcripts are higher than in wild-type Ws-2. Overall, diel changes of sugar levels are larger in *lhy cca1 elf3* than wild-type Ws-2 indicating a slightly weakened ability of the triple mutant to maintain sugar homeostasis.

### Dynamics of clock transcripts in short, neutral, and long photoperiods

The analyses in Dixon et al. (2011) and Supplemental Figure S3 showed that the circadian clock is severely disturbed when *lhy cca1 elf3* is grown in a recurring 12-h photoperiod under relatively high irradiance. We investigated the residual clock in other conditions when less C is available or when the plant is subjected to sudden perturbations (data provided in Supplemental Data file S2). We were interested to learn if the residual clock is severely disturbed across all treatments, or if there are any features that might explain the unexpected ability of the triple mutant to maintain a broadly wild-type-like pattern of starch mobilization across so many treatments.

**Figure 7:**
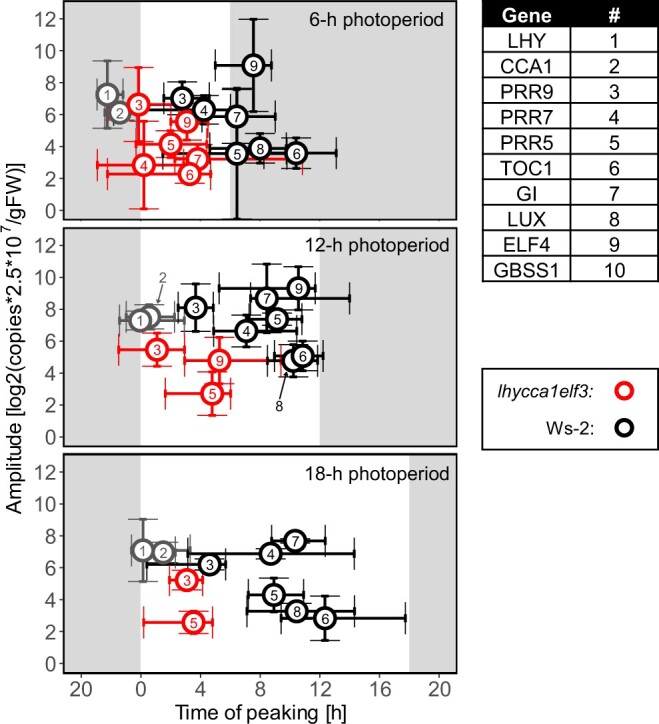
Summary of the peak time and amplitude of the diel oscillations of clock and *GBSS1* transcript abundance in different photoperiods. The display shows the mean peak time (*x*-axis) and the amplitude of oscillation (*y*-axis) for the *LHY*, *CCA1*, *PRR9*, *PRR7*, *PRR5*, *GI*, *TOC1*, *ELF4*, *LUX*, and *GBSS1* transcripts. Peak time was estimated as described in “Materials and methods.” The origin of the time axis is aligned to dawn and runs from ZT18 to ZT16 in order to capture all dawn-phased in the same part of the display (“ZT,” or “Zeitgeber” from the German language, indicates the time elapsed after the last dawn, in hours). Horizontal error bars indicate the bootstrapped 95% CI of the estimated peak time. The amplitude of oscillation (given as log_2_(copies × 2.5 × 10^7^ g^−1^ FW)), see methods for explanation of the unit) was estimated as the difference between maximum and minimum mean transcript level in a 24-h period. Vertical error bars indicate the 95% CI of the amplitude of oscillation. Wild-type Ws-2 and *lhy cca1* are indicated by black and red open symbols, respectively. Gene names are indicated by numbers. Background shading indicates the light period (white) and night (gray). The numerical values of these analyses are provided in Supplemental Data Set 3.

**Figure 8:**
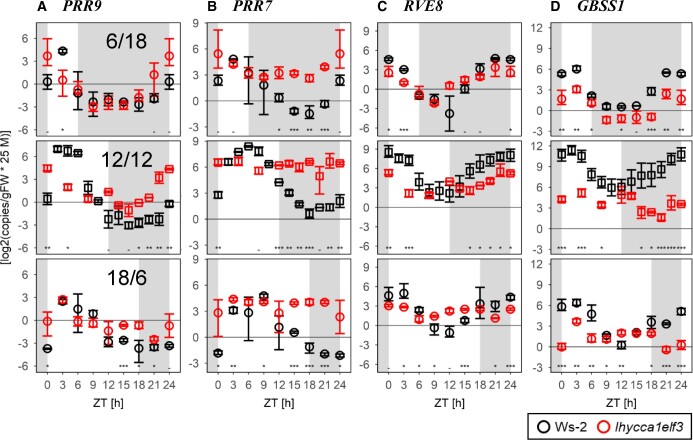
Response of selected transcripts in different photoperiods. A, *PRR9*. B, *PRR7*. C, *RVE8*. D, *GBBS1*. The three subparts show responses in a 6-h light/18-h dark, 12-h light/12-h dark and 18-h light/6-h dark cycle. Transcript measurements were carried out in the same material that was used for the starch analyses in a 12-h photoperiod in Figure 1A and in separate experiments for the 6-h light/18-h dark and 18-h light/6-h dark cycles. At each time point, two to five samples were harvested. Symbols represent the mean value and error bars indicate the bootstrapped 95% CI. Wild-type Ws-2 and *lhy cca1* are indicated by black and red symbols, respectively (see insert). Statistical significance (ANOVA, Sum of Squares type II) is indicated by asterisks (0 “***” 0.001 “**” 0.01 “*” 0.05); subsequent HSD Tukey’s post-hoc test is indicated by dashes (i.e. when not significantly different). “ZT,” or “Zeitgeber” from the German language, indicates the time elapsed after the last dawn, in hours. Plots for further transcripts are provided in Supplemental Figure S9. Statistical analyses of the extent of dawn alignment and dusk alignment are provided in Figure 9 and Supplemental Figure S10. Abundance is given as log_2_(copies × 2.5 × 10^7^/g FW).

We first investigated the response to photoperiod duration, using plant material from the 12-h photoperiod experiment of Figure 1, plus further independent experiments in 6-h and 18-h photoperiods (Supplemental Figure S1E). Figure 7 summarizes peak times and amplitude of the oscillations for all core clock genes that could be scored. It was difficult to score peaks for all genes in *lhy cca1 elf3*, especially in long-day conditions, hence less genes are shown for the long photoperiod treatments (see Supplemental Data file S3 and below). Responses of individual genes are provided in Figure 8 and Supplemental Figure S9A (left hand side, for discussion of the dusk-aligned plots, see below).

As seen in many previous studies, in wild-type plants most clock transcripts show large oscillations, peak sequentially during the first half of the diel cycle and, in accordance with the dawn dominance of the Arabidopsis clock, show only a small delay in peak time between a 6-h and 18-h photoperiod (Figure 7; see Figure 8, A and B and Supplemental Figure S9A for the detailed time courses). The response was very different in *lhy cca1 elf3* (Figure 7). First, the amplitude of the oscillations was smaller, with the attenuation becoming increasingly marked as photoperiod was lengthened. This pattern can be seen for *PRR9*, which showed the largest residual oscillation with a reduction to ∼50% in short days, and only a weak oscillation in an 18-h photoperiod (Figure 8A). Oscillations were even smaller for *PRR5*, *TOC1*, *GI*, *LUX*, and *ELF4* (Supplemental Figure S9A). The oscillation of *PRR7* transcript was essentially abolished in all photoperiods (Figure 8B). Second, all scorable transcripts peaked in the first 2–4 h of the 24-h cycle. For example, *PRR9* peaked at or just after dawn in all three photoperiods (Figure 8A), and *PRR5* and *ELF4* peaked at about ZT3 in a 6-h and 12-h photoperiod (Supplemental Figure S9A).

### Diel changes of transcripts of *RVE* and *PIF* family members, and of *GBSS1*

We next investigated the diel responses of *RVE* and *PIF* transcripts (Figure 8C; and Supplemental Figure S9, B and C). *RVE4*, *RVE6*, and *RVE8* act as positive regulators of dusk clock genes (Hsu et al., 2013; see also “Introduction”) and some PIFs mediate light and metabolic control of the clock (Shor et al., 2017; Liu et al., 2020). Furthermore, other *RVE* and *PIF* family members are important clock outputs (Kuno et al., 2003; Nozue et al., 2007; Rawat et al., 2009; Nusinow et al., 2011). Thus, the responses of *RVE* and *PIF* transcripts might provide complementary information about clock functionality in *lhy cca1 elf3*.

Diel oscillations of *RVE* transcripts were damped in *lhy cca1 elf3* compared to Ws-2 (Figure 8C; Supplemental Figure S9B; Supplemental Data Set 4). *RVE4* and *RVE8* showed attenuated peaks around dawn, whose peak time and the response to photoperiod nevertheless resembled those in Ws-2. *RVE8* transcript in Ws-2 and *lhy cca1 elf3* peaked before dawn in a 6-h photoperiod (ZT22.3 and ZT21.3, respectively) and later in an 18-h photoperiod (ZT1.0 and ZT1.6, respectively). *RVE4* transcript in Ws-2 and *lhy cca1 elf3* peaked at ZT20.8 and ZT21.8 in a 6-h photoperiod, respectively, and only slightly later at ZT22.0 and ZT22.4, respectively, in an 18-h photoperiod.

Oscillations of most *PIF* transcripts were strongly damped in *lhy cca1 elf3*, due mainly to abundance remaining high throughout the cycle (Supplemental Figure S9C). This largely reflects the response in the *elf3* mutant (Flis et al., 2019). The EC (formed by ELF3, ELF4, and LUX proteins; Nozue et al., 2007; Nusinow et al., 2011) is known to act to repress *PIF4* and *PIF5* in the second part of the 24-h cycle. Our results indicate EC may also repress other *PIF*s including *PIF2* and *PIF3*.

We also analyzed transcript abundance for the dawn marker *GBSS1* in *lhy cca1 elf3* (Supplemental Figure S9C). It displayed a weak peak at about ZT0–ZT3 but with a strongly attenuated oscillation, especially in 12-h or 18-h photoperiods. Overall, *RVE*, *PIF*, and *GBSS1* transcripts showed strongly attenuated oscillations and altered peak times in *lhy cca1 elf3* compared to Ws-2. The extent of the change depends on the gene and was smallest for *RVE4* and *RVE8*.

### Extent of dawn alignment of clock transcripts in *lhy cca1 elf3* in different photoperiods

The robust pacing of starch mobilization to dawn in different photoperiods might be linked in some way to the dawn dominance of the wild-type clock. As *lhy cca1 elf3* also paces starch mobilization to about 24 h after the previous dawn, we asked whether its residual clock operates in a dawn-dominant manner.

**Figure 9:**
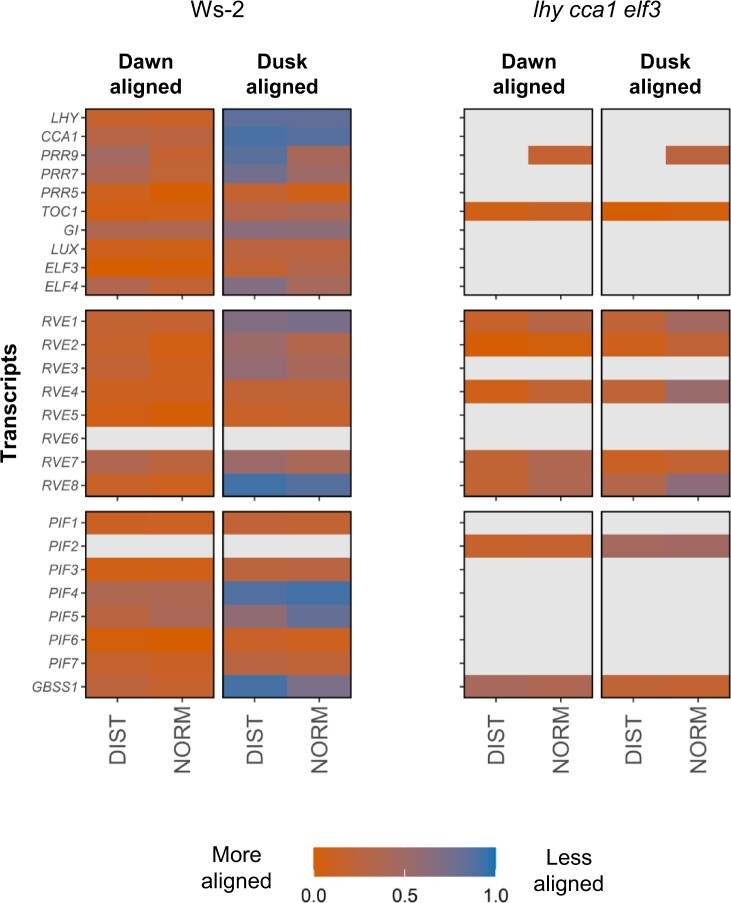
Mutual information analysis of the extent of alignment of transcript responses to dawn or dusk in 6/18-h, 12/12-h, and 18/6-h light/dark cycles. The analyses for Ws-2 are shown in the left hand and for *lhy cca1 elf3* in the right-hand block of plots. For each genotype, transcript and photoperiod the transcript time series data were organized as “dawn aligned” (i.e. profiles starting at light on) and as “dusk aligned” (i.e. profiles starting at light off; plots of the dawn- and dusk-aligned data series are provided in Supplemental Figure S9). The DIST score is defined as the Euclidean Distance between all treatments. The NORM score is defined as the Euclidean Distance divided by its propagated error (see Supplemental Data Set 4 for details). Heatmap colors indicate the average value of each score for each transcript; as explained in Supplemental Data Set 4. DIST and NORM scores were independently transformed to values between 0 and 1, with 0 indicating the best and 1 the worst alignment observed. ANOVA (Sum of Squares type II) and subsequent Tukey’s HSD was used to detect which transcripts were not significantly different between “dawn” and “dusk”; these are indicated as gray (at 95% CI level, *P* ≤ 0.5).

Quantitative analysis of diel oscillations is usually performed by comparing peak time. This was not possible for *lhy cca1 elf3* where weak oscillations often prevented reliable definition of peak time. Instead, we performed mutual information analysis (Figure 9) to test whether the expression of a gene during the 24-h cycle is more similar across different photoperiods when the 24-h cycle starts at dawn or dusk. For the dusk-aligned representation, we re-plotted the time series starting at the transition to darkness (i.e. dusk); which occurred at different ZT in each photoperiod (see Supplemental Figure S9 for plots of the dawn- and dusk-aligned 6-, 12-, and 18-h photoperiod time series). Our calculations used two different data preparation procedures (DIST and NORM, for details see Figure legends and “Materials and methods”). As expected, for Ws-2 all of the tested transcripts showed much greater similarity in the dawn-aligned time series than the dusk-aligned time series (Figure 9). This relationship was broken in *lhy cca1 elf3*. Oscillations of *TOC1* (in the NORM analysis only), *PRR9*, five *RVE*, and two *PIF* family members, and *GBSS1* transcript could be scored in *lhy cca1 elf3.* None of them differentiated between dawn and dusk alignment. We also performed two-way ANOVA to test whether the 6- and 18-h photoperiod time series were significantly different (i.e. desynchronized) when they were aligned to either dawn or dusk (Supplemental Figure S10; Supplemental Data Set 5). The responses of all transcripts were dawn-aligned in Ws-2, and this relation was weakened or broken in *lhy cca1 elf3*.

### The *lhy cca1 elf3* clock is advanced after a sudden early dusk

We next explored the response of clock and clock-related transcripts after an early dusk. In Ws-2, advancing dusk by 4 h led to only a small (∼2 h) advance in the response of most transcripts in the following night. In *lhy cca1 elf3* it resulted in a 4 h advance of the *PRR9* transcript and marked advances for many other transcripts (Supplemental Figure S11, see Supplemental Text S1 for details). Mutual information analysis confirmed that after a sudden early dusk in Ws-2 all transcripts remained aligned to the previous dawn, whereas in *lhy cca1 elf3* all genes that could be scored lost this alignment (Supplemental Figure S11D). This analysis indicates that the ability of the triple mutant to slow down starch mobilization after a sudden early dusk may depend on a temporal signal established earlier in the diel cycle.

### The *lhy cca1 elf3* clock is less robust than the wild-type clock in non-T24 cycles

Analyses in non-T24 cycles, especially T17 cycles, indicated that starch mobilization in *lhy cca1 elf3* is still paced by an internal 24-h rhythmicity (Figure 2). We compared transcript time series in T17, T24, and T28 cycles to learn whether residual features of the clock might contribute to this unexpected robustness. Clock progression was largely independent of T-cycle duration in Ws-2, but less so in *lhy cca1 elf3* (Supplemental Figure S12, see Supplemental Text S1 for details). For example, *PRR9* transcript abundance at external dawn in a T17 cycle differed from that ZT17 in a T24 cycle, and *PRR9* and *ELF4* transcript abundance at external dawn in a T28 cycle differed from that at ZT4 in a T24 cycle. Thus, *lhy cca1 elf3* maintains a fairly robust 24-h rhythmicity of starch turnover in non-T24 cycles even though many clock and clock-related transcripts deviate from 24-h rhythmicity.

### Transcripts in *lhy cca1 elf3* show acute responses to light and darkness

The finding that the residual clock in *lhy cca1 elf3* had weakened dawn alignment prompted us to ask if the remaining oscillations were acute responses to light or darkness. Inspection of Supplemental Figures S9–S12 revealed a small but consistent decrease of *PRR9*, *PRR5*, *ELF4*, *RVE3*, *RVE7*, *RVE8*, and *PIF2* transcript abundance after darkening *lhy cca1 elf3*. These might represent acute responses to darkness, as the decreases were absent or weaker in Ws-2. We also compared the responses of transcripts between ZT0 and ZT6 with those between ZT24-ZT30, that is, after a sudden 6 h extension of the night (Supplemental Figure S13; see Supplemental Text S1 for details). Sudden extension of the night had little effect on transcript abundance in Ws-2, as expected for oscillations that are driven by an endogenous clock. Transfer of *lhy cca1 elf3* to continued darkness led to large changes in the responses of *PRR9* and *ELF4* transcripts indicating that the residual diel oscillations are at least partly driven by acute signals related to light.

## Discussion

### The circadian clock in *lhy cca1 elf3* is severely disrupted with residual responses driven by acute responses to light, especially at dusk

The circadian clock is severely disturbed in the *lhy cca1 elf3* triple mutant, with loss of dawn component and *ELF3* function and attenuated and time-shifted oscillations in transcript abundance for almost all the remaining circadian clock genes, *RVE* and *PIF* family members and the dawn marker *GBSS1*. The *PRR9* transcript showed a more pronounced rhythm than in the original study of *lhy cca1 elf3* by Dixon et al. (2011). This may reflect differences in plant age, or growth conditions as the oscillation was strongly attenuated as photoperiod was lengthened. The disturbance of the clock in *lhy cca1 elf3* (Figures 7 and 8, Supplemental Figures S9–S13) is far more profound than in circadian mutants in which diel starch turnover was previously investigated (Graf et al., 2010; Mugford et al., 2014; Seki et al., 2017; Flis et al., 2019). The changes are partly due to loss of the dawn components and the associated loss of their repressor function on day and dusk components, and their functionality during clock entrainment at dawn. This is exacerbated by loss of EC function; during the second part of the 24-h cycle, many of changed responses in *lhy cca1 elf3* resemble those in the *elf3* mutant, including strong attenuation of the oscillations of transcripts of known EC targets like *PIF4* and *PIF5* (Nozue et al., 2007; Nusinow et al., 2011). In addition, comparison across photoperiods and analysis of the response to a sudden early dusk and in non-T24 cycles revealed that the residual clock in *lhy cca1 elf3* is less robustly dawn dominant than the wild-type clock (Figure 9; Supplemental Figure S10). This may be partly because some of the diel responses in *lhy cca1 elf3* are acute responses to light (Supplemental Figure S13).

Interestingly, *RVE1*, *RVE4*, and *RVE8* transcripts showed a less disturbed response in *lhy cca1 elf3* (Supplemental Figures S9B, S11B, and S12B) In wild-type plants, their expression peaks near to dawn and they act as positive regulators of clock genes, especially dusk components (Hsu et al., 2013; Adams et al., 2015). Their expression also peaked near to dawn in *lhy cca1 elf3*, although with a smaller amplitude than wild-type plants. Peak expression was delayed slightly in long compared to short photoperiod in *lhy cca1 elf3*, similar to the response in wild-type Ws-2. When *lhy cca1 elf3* was darkened 4 h before the normal dusk, the initial rise of *RVE1, RVE4*, and *RVE8* transcript abundance was advanced, but dawn levels resembled those in non-shifted plants. Expression of many *RVE* family members is regulated by C or light signals (Moraes et al., 2019). This might contribute to their relatively robust behavior in *lhy cca1 elf3*.

### Photoperiod-regulation of starch accumulation is severely compromised in *lhy cca1 elf3*

Wild-type plants accumulate starch more quickly in short photoperiods than in long photoperiods (Smith and Stitt, 2007; Mugford et al., 2014; Mengin et al., 2017). It was previously unclear whether the clock plays a major role in this response (see “Introduction”). Compared to wild-type plants, *lhy cca1 elf3* showed slightly slower starch accumulation in a 12-h photoperiod, much slower starch accumulation in a 6-h photoperiod and, crucially, was significantly compromised in its ability to speed up starch accumulation in the shorter photoperiod (Figure 1).

In wild-type plants, the increase in allocation to starch in short photoperiods is at least partly due to slower utilization of C for growth in the daytime, leading to elevated levels of sugars in the light (Sulpice et al., 2014; Mengin et al., 2017). Hydrolysis of sucrose to reducing sugars and their recycling to hexose phosphate is known to slow down net sucrose synthesis and increase allocation to starch, with posttranslational and allosteric activation of ADP glucose pyrophosphorylase (AGPase) playing a key role in this chain of events (Preiss, 1988; Huber, 1989; Kingston-Smith et al., 1999; Macrae and Lunn, 2006; Stitt et al., 2010). Substitution of the Arabidopsis AGPase by a deregulated bacterial AGPase effectively prevents the speeding up of starch accumulation in shorter photoperiods (Mugford et al., 2014). Interestingly, in short photoperiods, *lhy cca1 elf3* had even higher levels of reducing sugars in the light than wild-type Ws-2. This observation indicates that the weak response of starch accumulation to short photoperiods in *lhy cca1 elf3* is due to a restriction in the pathway of starch synthesis.

The impaired starch accumulation phenotype of *lhy cca1 elf3* was less obvious in its parents. Compared to wild-type plants, *lhy cca1* did not show any impairment of starch accumulation in a 12-h photoperiod (Flis et al., 2019) and speeded up starch accumulation in short photoperiods (Mugford et al., 2014). *elf3* had slightly slower starch accumulation and higher levels of reducing sugars in the daytime (Flis et al., 2019) pointing to a restriction in the pathway of starch synthesis similar to that seen in *lhy cca1 elf3* (see above). Nevertheless, *elf3* was able to speed up starch accumulation in short photoperiods (Mugford et al., 2014) although the acceleration might be weaker than in wild-type plants.

Taken together, these results highlight a role for the clock in increasing starch accumulation in short photoperiods. One possible explanation is that an inherently lower capacity for starch synthesis in *lhy cca1 elf3* prevents an increase in starch accumulation in short photoperiods. This aspect is evident in the *elf3* parent, although less markedly than in *lhy cca1 elf3.* Another would be that a severe disruption of clock function interferes with photoperiod-dependent signals that regulate growth and starch accumulation in the light period, synthesis, for example, by interfering with transmission of information from one 24-h cycle to the next.

### Quantitative analysis of the precision of the regulation of starch mobilization in wild-type plants

Previous studies with wild-type plants concluded that starch is mobilized in a liner manner at a rate that exhausts starch around 24 h after dawn, irrespective of growth conditions, short-term conditions and T-cycle duration (see “Introduction”). These conclusions were based on a largely qualitative analysis. Whilst assessing if mobilization is modified in *lhy cca1 elf3*, we carried out a more quantitative analysis of the dynamics of starch mobilization in wild-type Ws-2 (Figure 5).

These analyzes revealed that starch mobilization is paced rather precisely to 24 h after the previous dawn in a T24 cycle (23.5–25.8 h after the previous dawn in various experiments, resembling values in Flis et al., 2019; Moraes et al., 2019). While there was a small decrease (to 21.3 h after the previous dawn) in a T17 cycle, this was much smaller than the 7 h decrease in duration of the external light–dark cycle between a T24 and a T17 cycle. Our analyses also revealed that whilst mobilization is almost strictly linear in wild-type plants growing in a 12-h photoperiod in moderate irradiance, minor deviations in linearity appear in low irradiance or in short photoperiods and larger deviations appear when plants are subjected to sudden perturbations or are grown in a T28 cycle. Small deviations from linearity were also observed when plants were suddenly shifted to a different night temperature (Pyl et al., 2012).

Overall, the analysis of linearity underscores that whilst wild-type plants pace starch mobilization to around dawn across a wide range of conditions and perturbations, there are small deviations. Future assessment of the various models of starch mobilization (see “Introduction”) might gain by explicit consideration of this nonlinearity, which presumably reflects the precision of the underlying regulatory network. It might also be relevant to consider whether the network that regulates the rate of mobilization acts not only to slow down mobilization early in the night when starch content is high, but also to maintain rates of mobilization later in the night when starch content is low.

### *lhy cca1 elf3* is able to time starch mobilization to dawn across many conditions, although with slightly less precision than wild-type plants

Rather unexpectedly, *lhy cca1 elf3* retained the ability to pace starch mobilization to close to dawn. It achieved this across a wide range of photoperiod and irradiance growth regimes (Figure 1), after a sudden low irradiance day (Figure 3; Supplemental Figure S6) and, to a certain extent, after a sudden early dusk (Figure 4; Supplemental Figure S7). Quantitative analysis revealed that the timing may be slightly less precise than in wild-type plants. For example, compared to wild-type plants, *lhy cca1 elf3* retained slightly more starch at dawn in 12-h photoperiods, and slightly less starch at dawn in 6-h photoperiods. After an early dusk, the initial rate of starch mobilization was not decreased so strongly in *lhy cca1 elf3* as in wild-type plants. There was also a trend of less linear starch mobilization in *lhy cca1 elf3*, especially in short days and, possibly, after a sudden early dusk (Figure 5). Overall, the responses to changes in irradiance, including a single day of low irradiance, were more robust than responses to photoperiod or a sudden advance in dusk, which is in effect a sudden change in photoperiod.

Taken together, these results indicate that the regulatory network that paces starch mobilization to dawn is still operational in *lhy cca1 elf3*, although slightly weakened compared to the wild-type network. This slight weakening probably affects a component that delivers information about the time to dawn, although an impact on information about starch content cannot be excluded. The trend to increased nonlinearity was partly a consequence of slower mobilization when starch content is low. This might reflect a weakening in *lhy cca1 elf3* of the signal that drives rapid degradation in the last part of the night, when starch content is low.

It is also noteworthy that there is trend to lower sugars in the night in *lhy cca1 elf3* (Supplemental Figure S8) and that C starvation marker transcripts were more strongly induced at night in *lhy cca1 elf3* in a 12-h photoperiod at 160-µmol m^−2^ s^−^^1^ irradiance (Figure 6). This observation indicates that weaker circadian function results in weakened sugar homeostasis.

As already discussed, *lhy cca1 elf3* lacks *LHY* and *CCA1*, has nonfunctional ELF3 protein and abrogated EC function, and exhibits severely modified expression of the remaining clock genes, as well as many clock-related and clock output genes. The surprisingly robust pattern of starch mobilization in *lhy cca1 elf3* makes it very unlikely that any single component delivers the temporal input to the network that regulates starch mobilization. It is also unlikely that the temporal input derives from an antagonistic interaction between the dawn components and clock components whose expression peaks later in the 24-h cycle, as suggested by Flis et al. (2019). The opposed response of starch mobilization in *lhy cca1* and *elf3* and the reversion to a more wild-type pattern in *lhy cca1 elf3* is consistent with a genetic model in which the dawn components and evening components act antagonistically to slow down and speed up starch mobilization. However, the large-scale disruption of the residual clock in the triple mutant makes it questionable whether any such interaction occurs within the clock. Indeed, it is puzzling how such a disrupted clock could deliver any integrated signal to pace starch mobilization to dawn.

### *lhy cca1 elf3* paces starch with an approximately 24-h rhythmicity

Wild-type plants are able to pace starch mobilization to close to 24-h after the previous dawn when they are grown in a T17 cycle and, when initial rates are considered, a T28 cycle (Figure 2). This demonstrates that mobilization is regulated by an endogenous oscillator with a periodicity of ∼24 h (see “Introduction” and above). Remarkably, when *lhy cca1 elf3* was grown in T17 and T28 cycles it timed starch mobilization to close to 24 h after the previous dawn with almost the same precision as wild-type plants. This endogenous 24-h rhythmicity partly explains why *lhy cca1 elf3* is able to pace starch degradation to dawn across a wide range of conditions. However, the question remains how this rhythmicity is generated in a mutant with such a severely disturbed transcriptional clock.

Strikingly, starch mobilization was more resilient against the external T-cycle in the triple mutant than in its parents. *lhy cca1* paced starch mobilization according to its short internal rhythm in T17 and T24 cycles (ZT19 and ZT21, respectively) but not in a T28 cycle (ZT24.6). This might indicate that the clock imposes its rhythmicity on starch mobilization when the duration of the external light–dark cycle is close to the endogenous rhythm but not when the external cycle differs strongly from the internal period. An analogous argument can be made for the *elf3* mutant. Clock period cannot be scored because *elf3* is arrhythmic in free-running conditions, but in light–dark cycles *elf3* does show delayed peak times of dawn transcripts compared to wild-type plants (Flis et al., 2019). *elf3* paced starch mobilization to ZT27.4 and ZT28.5 in T28 and T24 cycles, but ZT22.3 in a T17 cycle (see below for further discussion).

### Possible explanations for how starch mobilization is paced to around 24 h after the previous dawn in a mutant with severely abrogated function of the transcriptional circadian clock

Several explanations can be offered for how *lhy cca1 elf3* paces starch mobilization to ∼24 h after the previous dawn. One is that regulation of translation, protein stability, or posttranslational modification maintains dawn-entrained oscillations at the level of core clock proteins abundance or functionality, and that these regulate starch mobilization. This explanation seems unlikely, as it would require that the protein or proteins are able to generate an output that regulates starch mobilization, but cannot correctly regulate canonical targets within the clock oscillator itself. It is in principle possible that gating of an output from a nonoscillating clock component might impact rhythmicity to starch turnover. However, this would require that the postulated gating mechanism is robust against the severe disturbance of the clock in *lhy cca1 elf3*.

A second possibility is that the temporal input that times starch mobilization is generated by clock or clock-related components whose oscillations are relatively robust against major disruption of the dawn, day, dusk and evening clock components. Although *PRR9* transcript did show oscillations in the triple mutant, these were strongly modified from those in wild-type Ws-2 and showed shifting alignment to dawn across different photoperiods, and poor 24-h rhythmicity in in non-T24 cycles. *ELF4* transcript retains an oscillation but with strongly altered phase, poor 24-h rhythmicity in non-T24 cycles and is anyway unlikely to be functional in the absence of ELF3 protein. One possible set of candidates might be some members of the *RVE* family. *RVE4*, *RVE6*, and *RVE8* are positive effectors of clock genes especially dusk and evening genes such as *TOC1* and *LUX* (Farinas and Mas, 2011; Rawat et al., 2011; Hsu et al., 2013; Shalit-Kaneh et al., 2018). As already discussed, *RVE4* and *RVE8* transcripts exhibited oscillations that, although attenuated, remained largely dawn-phased. Other *RVE* family members also retained oscillations including *RVE1* and *RVE7. RVE1* was reported as a clock output that affects diurnal auxin rhythms (Rawat et al., 2009). *RVE7* may form a semi-autonomous oscillator that is regulated by the dawn components *LHY* and *CCA1* and modulates rhythms in downstream clock outputs like *LHCB* (Kuno et al., 2003). Expression of many of these *RVE* family member may be regulated by C or light signals (Moraes et al., 2019), which might contribute to their relatively robust behavior in *lhy cca1 elf3*. Consistent with a role of RVEs in C-signaling, Gray et al. (2017) recently reported that *RVE4*, *RVE6*, and *RVE8* inhibit sucrose-promoted growth in seedlings and that *RVE3* and *RVE5* strengthen this inhibition. Another possibility is that bZIP63 contributes to the timing of starch turnover in *lhy cca1 elf3* (Viana et al., 2021). This would provide a SUCROSE NON-FERMENTING-RELATED KINASE1 (SnRK1) mediated input about C status, but would require that this input can function despite a severely disrupted clock.

A third possibility is that starch mobilization is timed by a semi-autonomous oscillator with a rhythmicity of ∼24 h, which is supervised by the clock but asserts its own rhythmicity when clock function is strongly abrogated. This idea is prompted by two observations. The first is that *lhy cca1 elf3* robustly times starch mobilization to 24 h after the previous dawn in non-T24 cycles. In this mutant, the clock may be so disrupted that it can no longer supervise the postulated semi-autonomous oscillator. The second relates to the timing of starch mobilization in non-T24 cycles in *lhy cca1* and *elf3* mutant. As already discussed, in both mutants starch is exhausted at the time when their clock anticipates dawn (i.e. the internal period of *lhy cca1*, when dawn transcripts peak for *elf3*) in T24 cycles (see also Graf et al., 2010; Scialdone et al., 2013; Flis et al., 2019) and also in non-T24 cycles whose dawn is fairly close to the time when they anticipate dawn, but not when there is a large mismatch. This would be explained if in these conditions the circadian clock no longer imposes its own rhythmicity on the network that paces starch mobilization to dawn. This explanation requires the reasonable assumption that the rhythmicity of the postulated semi-autonomous oscillator is likely to be ∼24-h, matching that of the circadian clock and the external light–dark cycle. Semi-autonomous or autonomous oscillators have been suggested in microbial, mammalian, and plant systems, including both nontranscriptional (van Ooijen and Millar, 2012; Reddy and Rey, 2014) and transcriptional (Kuno et al., 2003; Staiger et al., 2003) loops. Our proposed oscillator might capture information about photoperiod duration in preceding light–dark cycles, which in the field will be a good predictor of the duration of coming cycles and allow an approximate adjustment of starch turnover to coming events. There is mounting interest in the possibility that the duration of the night is sensed, and that this can occur at least partly via mechanisms that are independent of the circadian clock (Flis et al., 2016; Oakenfull and Davis, 2017; Seluzicki et al., 2017). It was also recently proposed that metabolic daylength measurement involving the F-box protein PHLOEM PROTEIN 2-A13 contributes to winter photoperiodism (Gendron et al., 2021; Liu et al., 2021). Interaction with or supervision of a semi-autonomous oscillator by the clock might provide increased robustness, for example, during rapid adjustment to sudden perturbations like a gloomy twilight. The latter might be the field scenario that is mimicked by the artificial experimental treatments in which light intensity is suddenly decreased or dusk is suddenly advanced. The larger deviation from linear starch mobilization when *lhy cca1 elf3* is subjected to a sudden change compared to when it is grown in stable conditions is consistent with the idea that the transcriptional clock aids adjustment to sudden changes.

Our analyses in the *lhy cca1 elf3* mutant do not conclusively distinguish between alternative models for the diel regulation of starch turnover. The sugar homeostasis model of Feugier and Satake (2013) and Seki et al. (2017) is in essence an oscillator composed of two interlocking functions, and can be formulated such that the oscillator operates largely independently of major changes in clock dynamics (Izumi, 2019). The molecular nature of these interlocking functions and their links to metabolic signals and the circadian clock are largely undefined. Our results exclude almost all known components of the Arabidopsis transcriptional clock from playing an essential role in such links. In particular, *lhy cca1 elf3* lacks functional *CCA1* and expresses *PRR7* at a high and near-constitutive level. In the arithmetic division model (Scialdone et al., 2013) the postulated entities that provide information about starch content (S) and time to dawn (T) are semi-autonomous functions. It can be envisaged that S is fully independent of the transcriptional clock, and that T is set by a mechanism that is at least partly independent of the transcriptional clock. Furthermore, our analyses indicate that starch accumulation and mobilization are separately regulated, as in the arithmetic division model.

In conclusion, the stimulation of starch accumulation in short compared to long photoperiods is attenuated in the higher-order circadian mutant *lhy cca1 elf3*, providing evidence that the circadian clock is involved in the diel regulation of starch accumulation. Unexpectedly, the regulatory network that paces starch mobilization to 24 h after the preceding dawn still operates in *lhy cca1 elf3*, and is only slightly weakened compared to wild-type plants, revealing that starch mobilization can be paced either by complementary components to those currently included in most clock models or by a semi-autonomous oscillator. It is, however, important to note that although our results show the clock functions that are disturbed in the triple mutant are nonessential, this does not mean they have no role in the regulation of starch mobilization in wild-type plants. An alternative scenario is that these clock functions do play a role in wild-type plants, but can be largely replaced by other signaling functions. This interpretation is supported by the fact that mutations in individual clock components often impact on starch mobilization (see “Introduction”). It is noteworthy that *lhy cca1 elf3* exhibits slightly larger diel changes in sugar levels and in the abundance of C-regulated transcripts. This raises the question whether signals deriving from metabolism compensate for a severe attenuation of clock function and maintain the near-wild-type pattern of starch mobilization that is found in the triple mutant. Irrespective of details, it can be envisaged that diel starch turnover is regulated by a flexible multi-layered network with partly redundant components. This complexity may be required to allow plants to, on the one hand, robustly pace starch degradation to dawn across a wide range of growth conditions, and, on the other hand, rapidly adjust the rate of starch degradation in response to day-to-day fluctuations in conditions.

## Materials and methods

### Plant material

The Arabidopsis (*A.thaliana*) wild-type accession Ws-2, the *lhy cca1 elf3* triple mutant (*lhy-21 cca1-11 elf3-4*; Dixon et al., 2011) and its parental lines the *lhy cca1* double mutant (*lhy-21 cca1-11*; Hall et al., 2003) and the *elf3* single mutant (*elf3-4*; Hicks et al., 2001) were kindly supplied by Prof. Dr. Andrew Millar’s Lab (University of Edinburgh, UK). The *elf3-4* (Ws-2 background) mutation results in a premature stop codon after the fortieth amino acid, as described by Hicks et al. (2001). The *lhy-21 cca1-11* double mutant (Ws-2 background) is the result of a crossing between two T-DNA insertion lines, *lhy-11* and *cca1-11* described by Hall et al. (2003). The *lhy-21 cca1-11 elf3-4* triple mutant was obtained by crossing the *lhy-21 cca1-11* double mutant and the *elf3-4* single mutant (as described by Dixon et al., 2011).

### Plant growth

Seeds were sown (spread in excess) on wet soil (Stender AG Schermbeck, Germany) mixed with vermiculite using plastic pots (10 cm of diameter) in 30 × 50 × 6 cm plastic trays. To avoid different developmental stages due to differing germination speed between genotypes, pots were initially kept for 1–3 days in a cold room (4°C) before transfer to growth chambers (Percival E-36 L, CLF Plant Climatics GmbH, Wertingen, Germany). Pots were covered with transparent plastic lids during the 5 initial days after sowing (DAS). Standard parameters for growth are provided in Supplemental Figure S1. After 1 week plants were thinned to avoid shade as needed. Pots were randomized every 3 days, or less. To avoid pests, plants were weekly treated with nematodes (*Caenorhabditis elegans*). Irradiance was decreased by dimming of tubular fluorescent lamps. Data for stable 24 h cycles was compiled from three sets of experiments: (1) *lhy cca1 elf3* in 12/12-h light–dark cycle at 160 µmol m^−2^ s^−1^ (Ws-2 data comes from Flis et al., 2015); (2) *lhy cca1 elf3* and Ws-2 in 12/12-h light–dark cycles at 90 and 60 µmol m^−2^ s^−1^; and (3) *lhy cca1 elf3* and Ws-2 in 6/18-h light–dark cycles at 160 µmol and 90 m^−2^ s^−1^. Comparisons of results in stable non-24 h were done using independent experiments conducted in 17- and 28-h diel cycles in combination with results from the above set of experiments (1) in 24 h diel cycles. Experiments in fluctuating conditions were always conducted in parallel with a control group (i.e. sudden early dusk and sudden low light). The protocol on the harvest day is described in Supplemental Figure S1. At each sampling point, three or more biological replicates (missing values are indicated as “NA” in Supplemental Data Sets 1 and 2) were harvested from different parts of the chamber into liquid nitrogen.

### Biomass composition

Fresh weight per plant was estimated for both *lhy cca1 elf3* mutant and Ws-2 wild-type plants using ZT0 samples of the 17, 24, and 28 h diurnal cycle experiments indicated in Supplemental Figure S1, A and B. It was defined as the quotient between each sample’s weight (in grams; measured immediately prior freezing in nitrogen) and the number of individuals in each pool (Supplemental Data Set 1).

### Transcripts

RNA was extracted and transcripts analyzed by RT-qPCR (three technical replicates). Absolute quantification of copy number per gram of fresh weight was performed using artificial RNA internal standards as in Flis et al. (2015). Results were divided by a factor of 25 million (Flis et al., 2015) and transformed to base 2 logarithm when necessary.

### Metabolites

Starch and metabolites were assayed (two technical replicates per biological replicate) as previously (Gibon et al., 2002; Cross et al., 2006; Nunes-Nesi et al., 2007).

### Data processing and visualization

All data analyses and their visualization were performed using the open source R language and its publicly available packages (Fox and Weisberg, 2002; Wickham, 2007, 2009, 2011; Gregory and Warnes, 2015; Harrell Jr, 2015; De Mendiburu, 2016; R Core Team, 2017; Wickham and Seidel, 2018; Pedersen, 2019).

### Statistical analysis of time series

Significance of changes in metabolite and transcript levels was tested using analysis of variance (ANOVA; Herr, 1986; Langsrud, 2003), comparing each treatment with the control group at each time point, and on all time points in a defined time interval. Results were subsequently analyzed using Tukey’s HSD post-hoc test (Tukey, 1949).

### Time of peak and amplitude analysis

Time of peak was calculated based on the average peak timing of bootstrapped time series using inflexion points in both polynomial fit and spline smooth (50 repetitions for each method). Fits were optimized for minimum second-order Akaike’s Information Criterion. Peak amplitude of circadian oscillation was defined as the difference between maximum and minimum mean expression level for a given gene in a given condition. Gaussian error propagation (Birge, 1939; Ku, 1966) was used to estimate the error of the amplitude of circadian oscillation calculated; which was then used to estimate the 95% CI.

### Calculation of the rates of starch accumulation and mobilization and the expected time of StEx

Rates of starch accumulation and mobilization were estimated as the slope of the fit linear models describing starch levels as a function of time, applied to data (biological replicates were not averaged) for the light and dark periods, respectively. Rates of starch mobilization in the sudden early dusk experiments were calculated using smaller subsets of the dark periods. The 95% CI was estimated from a normal Student’s *t* distribution using the standard error of the slope.

In experiments with different growth photoperiod and irradiance in T24 cycles, the expected time of StEx was estimated from the root of the linear fitting (i.e. the ZT value when starch amount equals zero; f(ZT) = 0) using a nonderivative approach (Anderson, 1974). In non-T24 comparisons, to improve the statistical quality of this assessment the StEx^*app*^ was estimated by extrapolating the initial rate of starch mobilization, using multiple fittings to a progressively larger number of consecutive time points (never smaller than three) using starch content at dusk and in the first 8.5 h of the night. Final results were averaged (i.e. geometric mean; *n* = 3 for all genotypes in all T-cycles) and reported in combination with its 95% CI (normal Student’s *t* distribution; calculated using the standard error of the average).

### Calculation of an ad hoc score for nonlinearity of starch mobilization

A straight line was interpolated between dusk and dawn starch reference values to provide, at any given time, a predicted starch content if starch mobilization were to be linear. At each harvest time point, the predicted starch content was subtracted from the measured starch content, and the difference (residue) normalized on the predicted starch content. These normed residues were then averaged across all time points. The 95% CI of the nonlinearity score was calculated using a nonparametric bootstrap procedure (Harrell Jr, 2015). The score takes a value of zero if starch mobilization is linear, negative if starch is initially mobilized more quickly and positive is starch is initially mobilized more slowly than would be expected from linear mobilization.

### Accession numbers

Accession number/identifier of genes cited in this article can be found in Supplemental Text S2.

## Supplemental data

The following materials are available in the online version of this article.

**Supplemental Figure S1.** Experimental design.

**Supplemental Figure S2.** Biomass and chlorophyll.

**Supplemental Figure S3.** Diel changes of clock transcript abundance in a stable 12–h light/12–h dark cycle at 160 µmol m^−2^ s^−1^ irradiance.

**Supplemental Figure S4.** Diel starch turnover in wild-type Ws-2 and the triple *lhy cca1 elf3* mutant, replotted with starch content normalized on the dusk starch content.

**Supplemental Figure S5.** Analysis of the dynamics of diel starch turnover in wild-type Ws-2, *lhy cca1 elf3* and its parents *lhy cca1* and *elf3* in different T-cycles.

**Supplemental Figure S6.** Response of starch degradation after a single day of low irradiance, with starch normalized on dusk starch content.

**Supplemental Figure S7.** Response of starch mobilization after a sudden early dusk, three further experiments that replicate the experiment of Figure 4.

**Supplemental Figure S8.** Sugar content.

**Supplemental Figure S9.** Diel response of transcript abundance in different photoperiods.

**Supplemental Figure S10.** Two-way ANOVA test of whether the oscillations of transcript abundance in different photoperiods and after a sudden early dusk are dawn-or dusk-aligned in Ws-2 and *lhy cca1 elf3.*

**Supplemental Figure S11.** Response of transcript abundance to a sudden early dusk.

**Supplemental Figure S12.** Response of transcript abundance in different T cycles.

**Supplemental Figure S13.** Response of transcript abundance in an extended night.

**Supplemental Text S1.** Dynamics of transcripts after a sudden early dusk and in non T24 cycles, and acute response of clock transcripts to light and dark.

**Supplemental Text S2.** TAIR, UniPROT, and EMBL accession number/identification.

**Supplemental Data Set 1.** Metabolite content and biomass accumulation.

**Supplemental Data Set 2.** Transcript abundance.

**Supplemental Data Set 3.** Estimated peak time and amplitude of the diel oscillations of clock and *RVE4*, *RVE8* and *GBSS1* transcript abundance in different photoperiods.

**Supplemental Data Set 4.** Mutual Information analysis of the photoperiod and early dawn time series.

**Supplemental Data Set 5.** Two-way ANOVA of the photoperiod and sudden early dusk time series.

## Supplementary Material

kiac226_Supplementary_Data

## Notes

T.A.M. contributed to experimental design, performed all experiments, statistically analyzed all data, and contributed to paper writing. V.M. assisted in sampling and in determination of transcripts. B.P. assisted in sampling. U.K. assisted in determination of transcripts. B.E., N.K., and M.H. assisted in determination of metabolites. M.S. conceived the project, assisted data analysis, and wrote the paper (senior author).

The author responsible for distribution of materials integral to the findings presented in this article in accordance with the policy described in the Instructions for Authors (https://academic.oup.com/plphys/pages/general-instructions) is Thiago Alexandre Moraes (ta507@cam.ac.uk).
