# Supplementary material for: The circadian clock mutant lhy cca1 elf3 paces starch mobilization to dawn despite severely disrupted circadian clock function
Source: Plant Physiol. 2022 May 14;189(4):2332–56. doi: 10.1093/plphys/kiac226 (PMC9348821; doi:10.1093/plphys/kiac226)
Supplement: kiac226_Supplementary_Data [file kiac226_supplementary_data.zip › kiac226_Supplementary_Data/DeSYNC_Suppl. Data.pdf]

Supplemental Figure S1 – Experimental design

Schematic depiction of the growth conditions, the conditions immediately prior to and during harvesting, harvesting times and sample numbers. The time spent in a given condition is indicated in each scheme. The light period is shown as yellow-orange colors (increasingly deep orange hue signifies lower irradiance) and the night as black. Depending on the treatment, plants were 14-16 days old on the day of harvesting (for one exception see panel E).

(A) Experiments where plants were grown in a T24 cycle with stable photoperiod and irradiance. In the experiments with low irradiance, plants were initially grown at a higher irradiance to allow biomass accumulation, and were transferred to the final irradiance three to five days before the day on which samples were harvested. All experiments were analyzed for starch (Fig. 1) and metabolites (Suppl. Fig. S8). The experiment with a 12-h photoperiod and 160 µmol m-2 s-1 irradiance was analyzed for transcript abundance (Suppl. Fig. S3, also included in Fig. 8 and Suppl. Fig. S9). Transcripts were not analyzed in the 6-h photoperiod experiment due to the small size of the samples, but were instead analyzed in the separate experiment of panel E. Metabolite (Fig. 2B and Suppl. Fig. S5) and transcript (Suppl. Fig. S3) data for *lhy cca1* and *elf3* parental mutants are from Flis et al., 2015, 2019.

(B) Experiments where plants were grown in different T-cycle duration. The experiment was analyzed for starch (Fig. 2), metabolites (Suppl. Fig. S8) and transcripts (Suppl. Fig. S12).

(C) Sudden low irradiance day. Plants were grown in a 12-h photoperiod at 160 µmol m^{-2} s^{-1} irradiance for 13 days and then left at growth irradiance or transferred to 90 µmol m^{-2} s^{-1} irradiance at ZT0 on the day of the experiment (“ZT”, or “Zeitgeber” from the German language, indicates the time elapsed after the last dawn, in hours), and harvested from ZT12 onwards (i.e. at dusk and through the night). The experiment was analyzed for starch (Fig. 3), metabolites (Suppl. Fig. S8) and transcripts (Fig. 8 and Suppl. Fig. S12).

(D) Sudden early dusk. Plants were grown in a 12-h photoperiod at 160 µmol m^{-2} s^{-1} irradiance and then either left in growth conditions or darkened at ZT8 (i.e. 4 h before the expected time of dusk). The experiment was repeated 4 times, with slightly differences sampling times (indicated by numbers in the harvest day). The plants in experiments #3 and #4 were in their 22nd and 14th day after sowing, respectively, and were also sampled in extended darkness from ZT24 to ZT30. Control data (12-h photoperiod at 160 µmol m^{-2} s^{-1} irradiance) for experiment #4 comes from the experiment in Suppl. Fig. S1A (shown originally in Fig.1). The plants in experiments #11 and #27 were in their 14th day after sowing. All four experiments were analyzed for starch (Fig. 4, Suppl. Fig. S7), and the experiment of Fig. 4 was also analyzed for metabolites (Suppl. Fig. S8) and transcripts (Suppl. Fig. S11).

(E) Repeat of the 6-h photoperiod, and 18-h photoperiod experiments. The experiments were analyzed for transcripts (Suppl. Fig. S8 and S9).

Supplemental Figure S1 – Experimental design (continued)

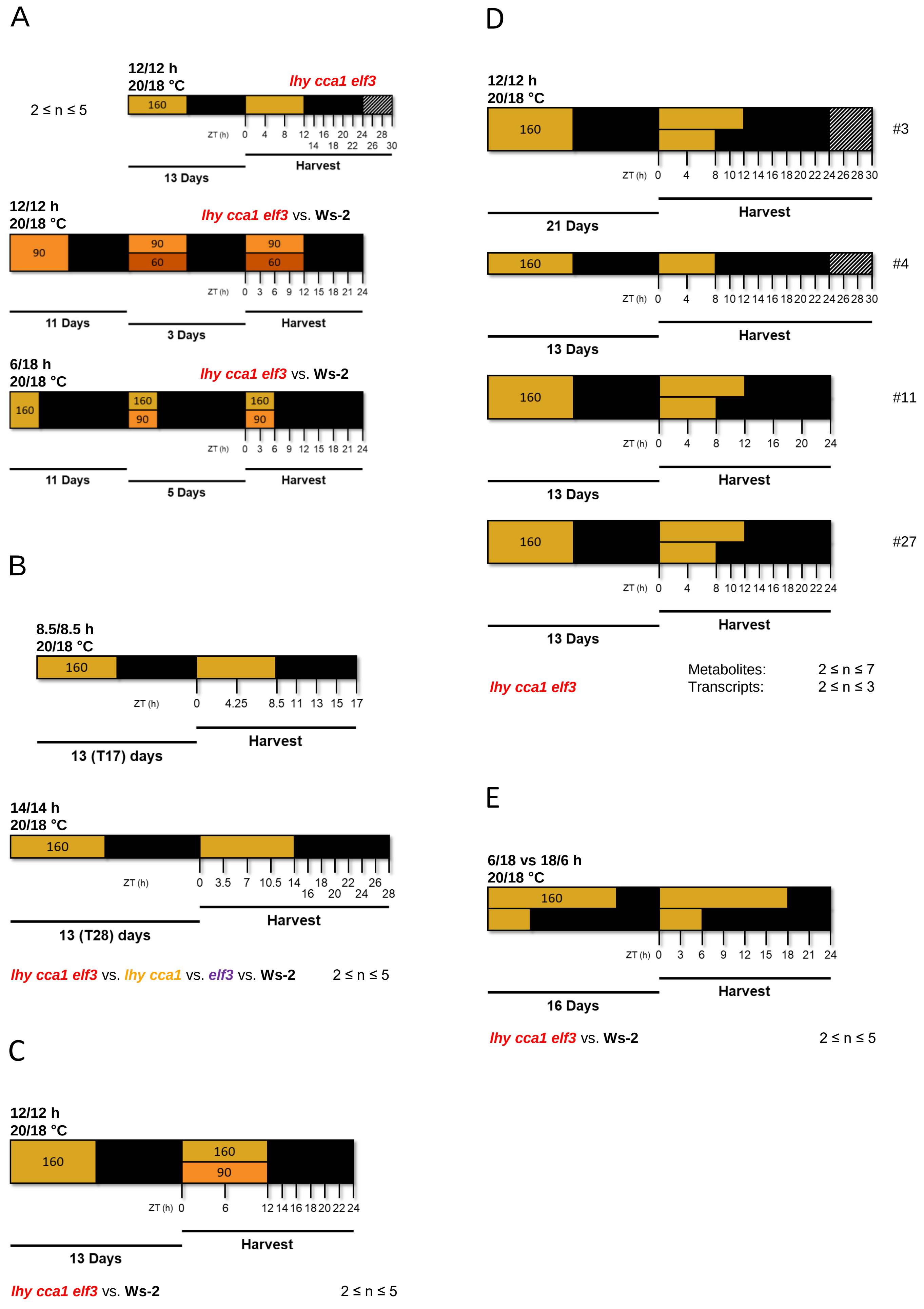

Supplemental Figure S2 – Biomass and Chlorophyll

(A) Biomass in T17, T24 and T28 diurnal cycles, determined in the experiments of Suppl. Fig. S1A and S1B using samples collected at dawn (3 to 6 biological replicates per genotype per T-cycle). Indication of fresh weight per plant of each biological replicate for a given genotype at dawn of the harvest day.

(B) Chlorophyll content in a T24 cycle with a 12/12 hour or a 6/18 hour photoperiod and different irradiance, determined using samples from all time points (3 biological replicates per genotype per time point) in the experiments of Suppl. Fig. S1A.

The plots in Panels A and B show the mean value and error bars indicate the bootstrapped 95% confidence interval. Black denotes wild-type Ws-2 and red denotes *lhy cca1 elf3*. Statistical significance (ANOVA, sum of squares type II and subsequent HSD Tukey's post-test) is indicated by letters (at 95% confidence level, p-value ≤ 0.5).

A. Biomass

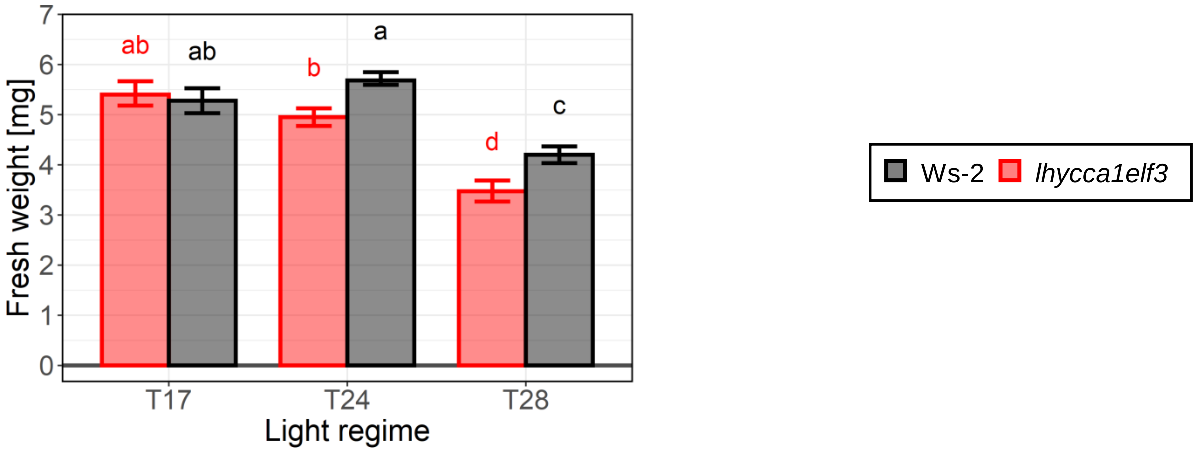

B. Chlorophyll content

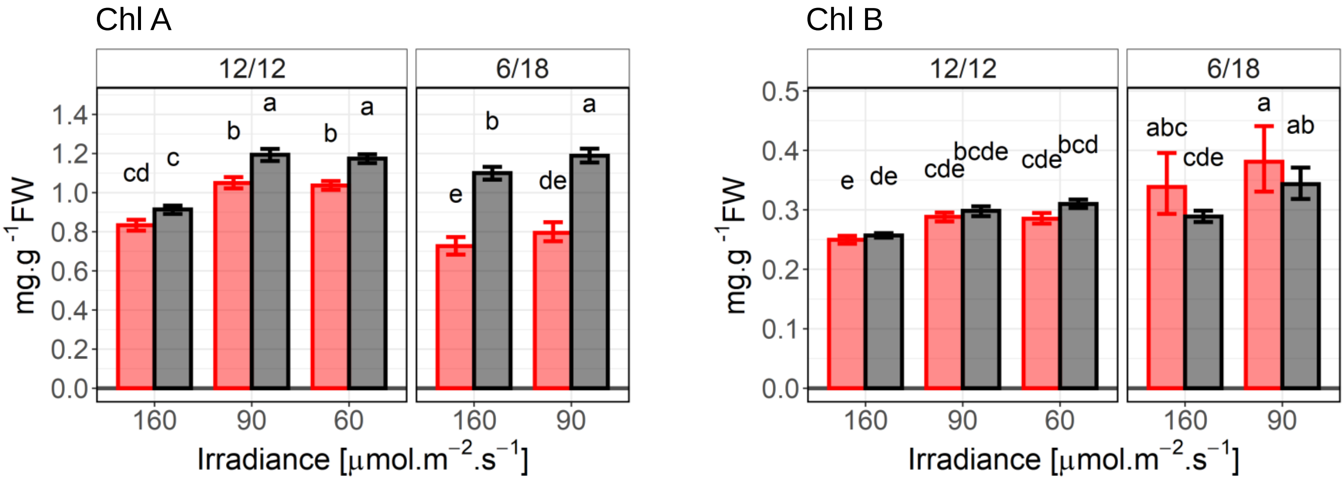

Supplemental Figure S3 – Diel changes of clock transcript abundance in a stable 12 h light / 12 h dark cycle at 160 µmol m^{-2} s^{-1} irradiance

Light period and night are indicated by white and grey shading, respectively.
The **left-hand panels show transcript abundance in wild-type Ws-2 (black) and *lhy cca1 elf3* (red)**, determined in the experiment of Suppl. Fig. S1A (same material as used for the measurement of starch in Fig. 1).

The **middle panels show transcript abundance in wild-type Ws-2 (pale black), *lhy cca1*(orange) and *elf3* (purple)**. Ws-2 is replicated from the left-hand panels. The data are from Flis et al. (2015, 2019). These responses are reproduced later in the current manuscript (Suppl. Fig. S12).

The **right-hand panels show transcript abundance in wild-type Col-0 (black) and *prr7 prr9* (blue), *toc1* (green) and *gi* (magenta)**. The data are from Flis et al. (2015, 2019).

The plots show the mean and 95% confidence interval (calculated using a non-parametric bootstrap procedure) of the non-linearity score. At each time point, 2 to 5 samples were harvested. Statistical significance (ANOVA, sum of squares type II) is indicated by asterisks (0 '***' 0.001 '**' 0.01 '*' 0.05); subsequent HSD Tukey's post-test is indicated by dashes (i.e. when not significantly different). NA denotes that a test was not applicable due to lack of replicates. "ZT", or "Zeitgeber" from the German language, indicates the time elapsed after the last dawn, in hours.

Supplemental Figure S3 – Diel changes of clock transcript abundance in a stable 12 h light / 12 h dark cycle at 160 µmol m^{-2} s^{-1} irradiance (continued)

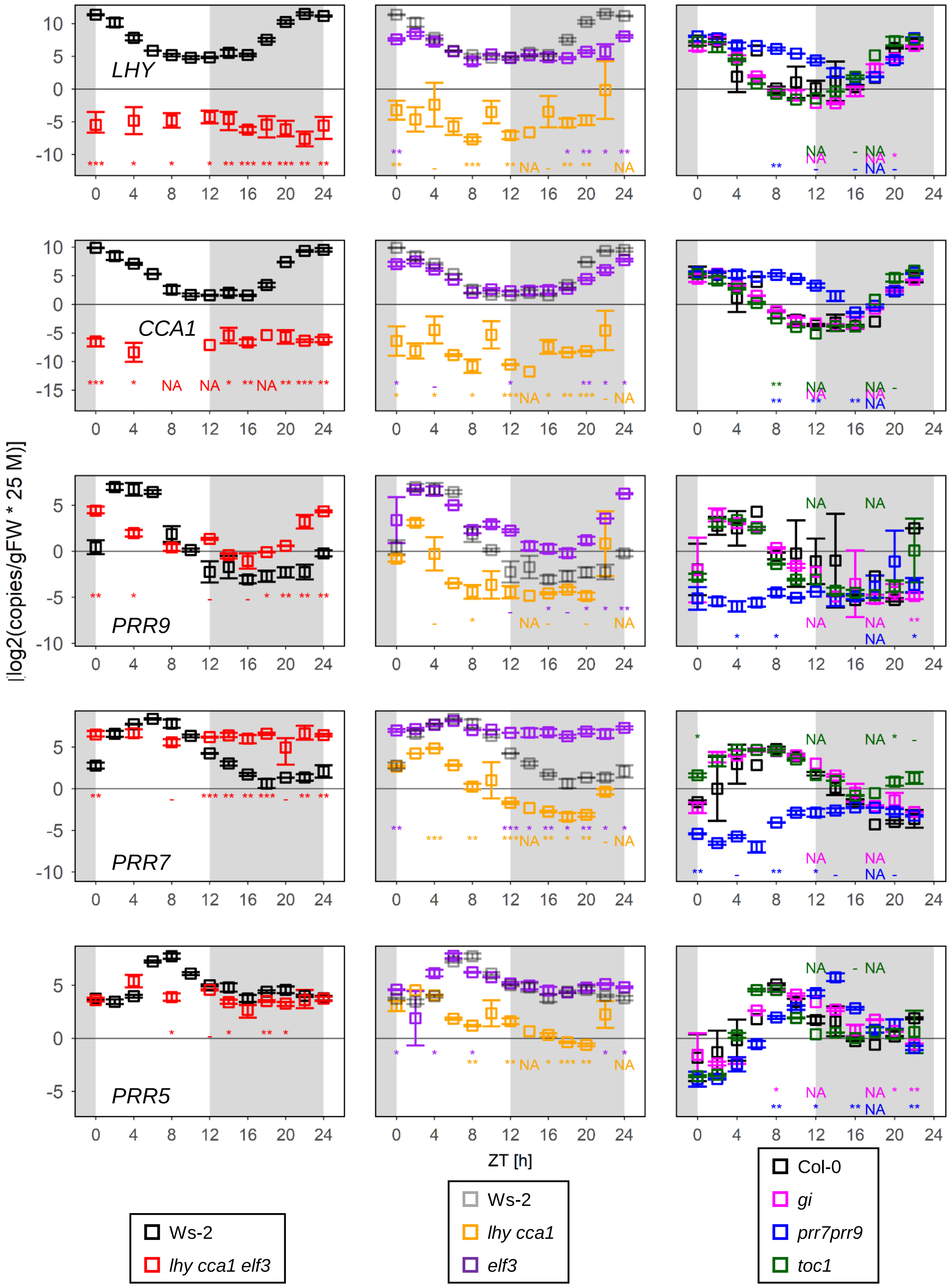

Supplemental Figure S3 – Diel changes of clock transcript abundance in a stable 12 h light / 12 h dark cycle at 160 µmol m^{-2} s^{-1} irradiance (continued)

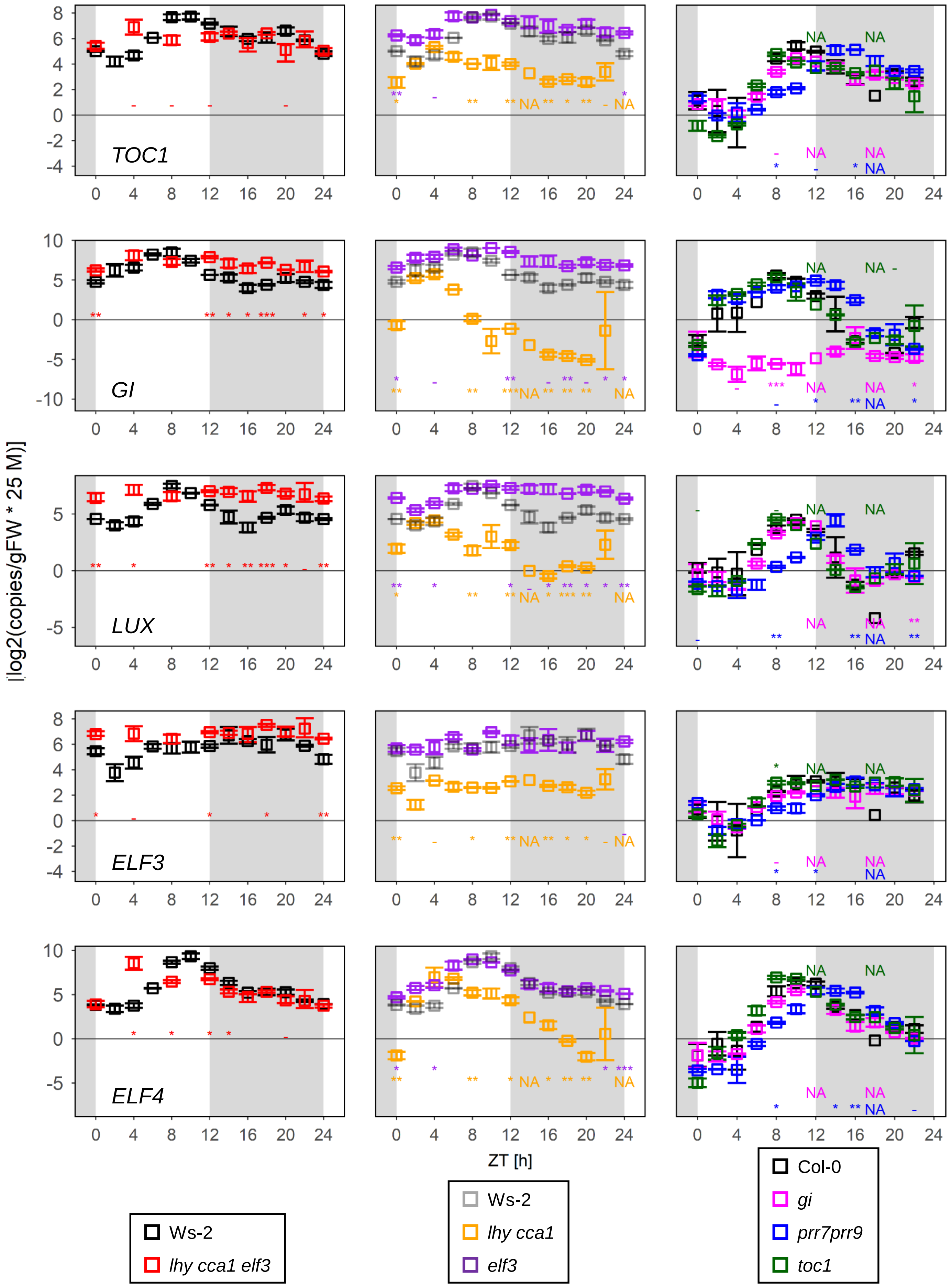

Supplemental Figure S4 – Diel starch turnover in wild-type Ws-2 and the triple *lhy cca1 elf3* mutant, replotted with starch content normalized on the dusk starch content

These plots further analyze the starch content data from Fig. 1, out of the experiments of Suppl. Fig. S1A. Normalization on dusk starch content allows comparison of starch mobilization across treatments where the dusk starch content is different – vis between different photoperiods, and especially between Ws-2 wild type and *lhy cca1 elf3* in a 6-h photoperiod. Wild-type Ws-2 and *lhy cca1 elf3* are shown as black and red symbols, respectively. At each time point, 2 to 5 samples were harvested.

(A) Diel changes in normalized starch content. The background shading identifies the light period (white) and the night (grey). The plot shows the mean value and error bars indicate the bootstrapped 95% confidence interval. Statistical significance (ANOVA, sum of squares type II) is indicated by asterisks (0 '***' 0.001 '**' 0.01 '*' 0.05); subsequent HSD Tukey's post-test is indicated by dashes (i.e. when not significantly different). Missing values in the *lhy cca1 elf3* mutant are indicated as 'NA'. "ZT", or "Zeitgeber" from the German language, indicates the time elapsed after the last dawn, in hours.

(B) Estimated time at which starch is exhausted. Symbols represent the projected time of starch exhaustion as defined by the geometric mean of multiple projections ($StEx^{app}$). The latter were performed using linear models on time spans of varying length that started at dusk and extended for increasing lengths of time into the night (at least three time points). Error bars indicate the 95% confidence interval of the multiple estimations. Solid upward and downward triangles denote 90 and 160 µmol m^{-2} s^{-1} irradiance, respectively, in the 6 h /18 h and open squares, diamonds and circles denote 60, 90 and 160 µmol m^{-2} s^{-1} irradiance, respectively, in the 12 h light / 12 h dark photoperiod. Color indicates different genotypes, as in panel A. Numeric values are provided in Suppl. Dataset S1.

Supplemental Figure S4 – Diel starch turnover in wild-type Ws-2 and the triple *lhy cca1 elf3* mutant, replotted with starch content normalized on the dusk starch content (continued)

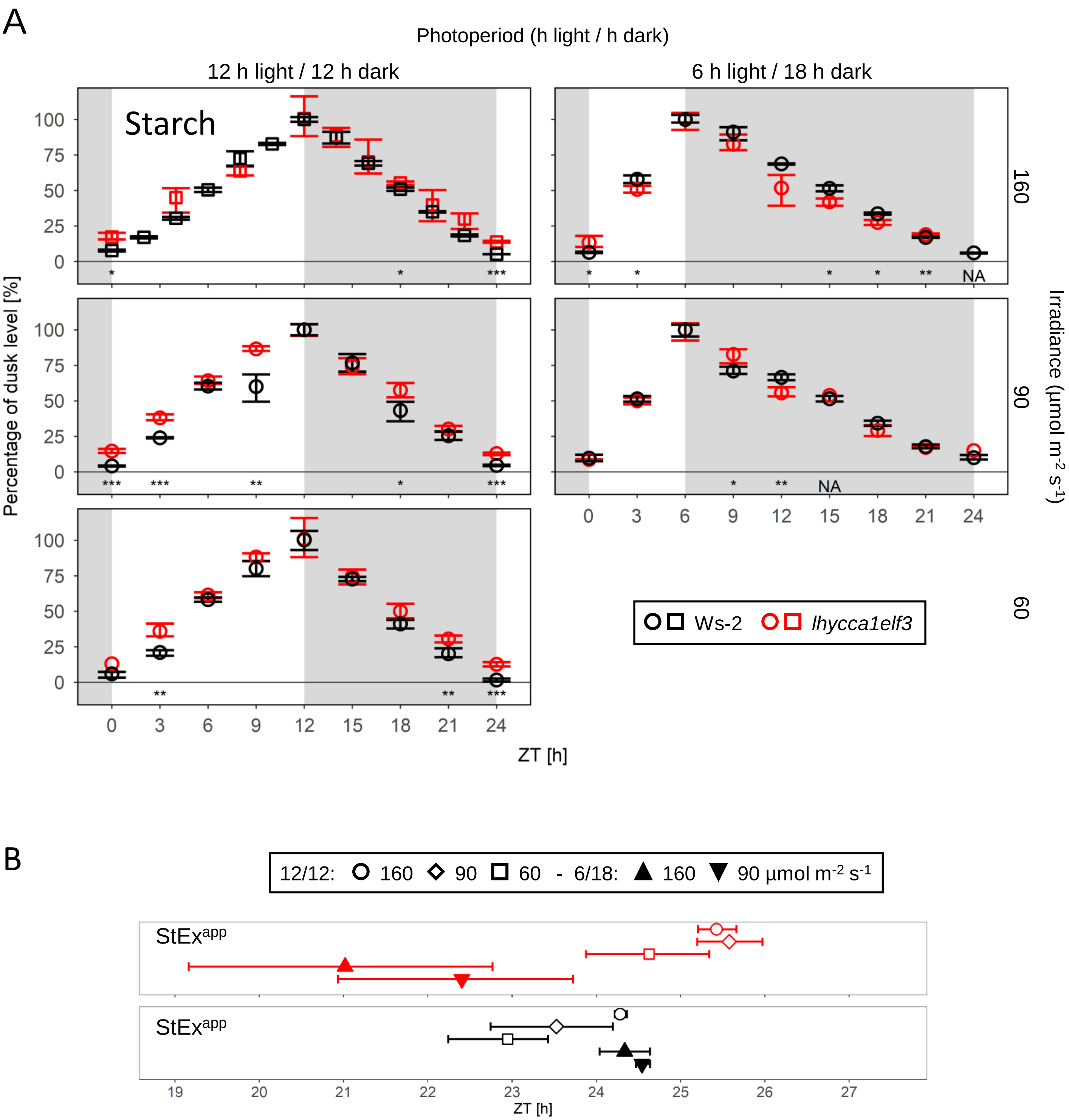

Supplemental Figure S5. Analysis of the dynamics of diel starch turnover in wild-type Ws-2, *lhy cca1 elf3* and its parents *lhy cca1* and *elf3* in different T-cycles

(A) Diel changes in normalized starch content in an 8.5 h light / 8.5 h dark (T17, left panel) and 14 h light / 14 h dark (T28, right hand panel) normalized on dusk starch content to aid comparison of the rate of starch mobilization relative to dusk starch content. The plots further analyze data from Fig. 2, out of the experiment of Suppl. Fig. S1A-B. The data for Ws-2 in a 12-h light / 12-h dark (T24) cycle is from Fig. 1A and a second separate experiment (i.e. the control for Exp. 11 in Suppl. Fig. S7). The data for *lhy cca1* and *elf3* in a T24 cycle is from Flis et al., 2019. Normalization on dusk starch content aids comparison of starch mobilization across treatments where the dusk starch content is different (here, in different T-cycles and different genotypes). At each time point, 2 to 5 samples were harvested. The background shading identifies the light period (white) and the night (grey). Wild-type Ws-2, *lhy cca1 elf3* and its parents *lhy cca1* and *elf3* are shown as black, red, orange and purple symbols, respectively. The plot shows the mean value and error bars indicate the bootstrapped 95% confidence interval. Statistical significance (ANOVA, sum of squares type II) is indicated by asterisks (0 '***' 0.001 '**' 0.01 '*' 0.05) for each mutant; subsequent HSD Tukey's post-test was significant in all cases). "ZT", or "Zeitgeber" from the German language, indicates the time elapsed after the last dawn, in hours.

(B) Analysis of the extent to which starch mobilization is non-linear in a T17, T24 or T28 cycle, all with equal duration of light period (irradiance 160 µmol m^{-2} s^{-1}) and darkness. The analysis was conducted as described in Fig. 5. Wild-type Ws-2, *lhy cca1 elf3*, *lhy cca1* and *elf3* are indicated by black, red, orange and purple symbols, respectively (see insert). All plants were harvested 13 diel cycles after sowing. The plots show the mean and 95% confidence interval (calculated using a non-parametric bootstrap procedure) of the non-linearity score. At each time point, 2 to 5 samples were harvested. Significant statistical differences (indicated by letters; at 95% confidence level, p-value ≤ 0.5) between groups of results in each panel was analyzed using ANOVA and subsequent HSD Tukey's post-test (significant in all cases). Numeric values are provided in Suppl. Dataset S1.

Supplemental Figure S5. Analysis of the dynamics of diel starch turnover in wild-type Ws-2, *lhy cca1 elf3* and its parents *lhy cca1* and *elf3* in different T-cycles (continued)

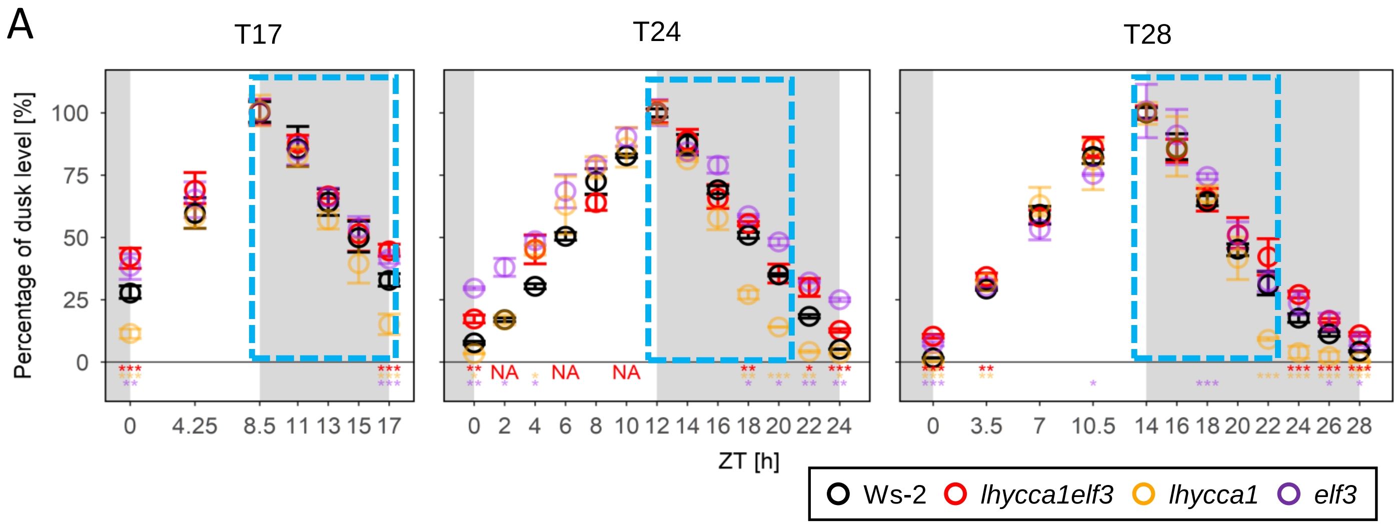

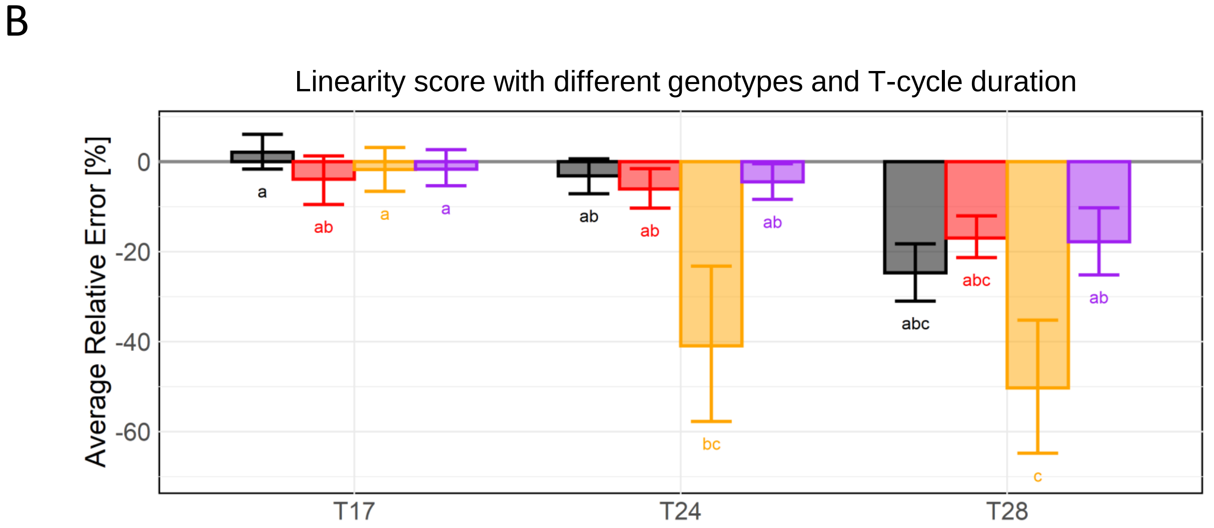

Supplemental Figure S6. Response of starch degradation after a single day of low irradiance, with starch normalized on dusk starch content

These plots further analyze data from Fig. 3 (experiment in Suppl. Fig. S1C). Normalization on dusk starch content allows comparison of starch mobilization across treatments where the dusk starch content is different, in this case because starch accumulation in the preceding light period was slowed down by low irradiance. Wild-type Ws-2 and *lhy cca1 elf3* are shown as black and red symbols, respectively. At each time point, 2 to 5 samples were harvested.

(A) Changes of normalized starch content during the night. The background shading identifies the light period (white) and the night (grey). The left-hand panel shows wild-type Ws-2 and the right-hand panel shows *lhy cca1 elf3*. The plots show the normalized starch content at different times during the night in plants that were left at growth irradiance (160 µmol m^{-2} s^{-1}, open symbols) and plant that had been transferred to lower light (90 µmol m^{-2} s^{-1}, closed symbols) in the preceding light period. The symbols show the mean value and error bars indicate the bootstrapped 95% confidence interval. Statistical significance (ANOVA, sum of squares type II) is indicated by asterisks and dots (0 '***' 0.001 '**' 0.01 '*' 0.05; subsequent HSD Tukey's post-test was significant in all cases). "ZT", or "Zeitgeber" from the German language, indicates the time elapsed after the last dawn, in hours.

(B) Estimated time at which starch is exhausted. Symbols represent the projected time of starch exhaustion as defined by the geometric mean of multiple projections ($StEx^{app}$). These were performed using linear models on time spans of varying length that started at dusk and extended for increasing lengths of time into the night (at least three time points). Error bars indicate the 95% confidence interval of the multiple estimations. Solid and open symbols indicate results from "Low light" and "Control" conditions, respectively. Color indicates different genotypes, as in panel A. Numeric values are provided in Suppl. Dataset S1.

Supplemental Figure S6. Response of starch degradation after a single day of low irradiance, with starch normalized on dusk starch content

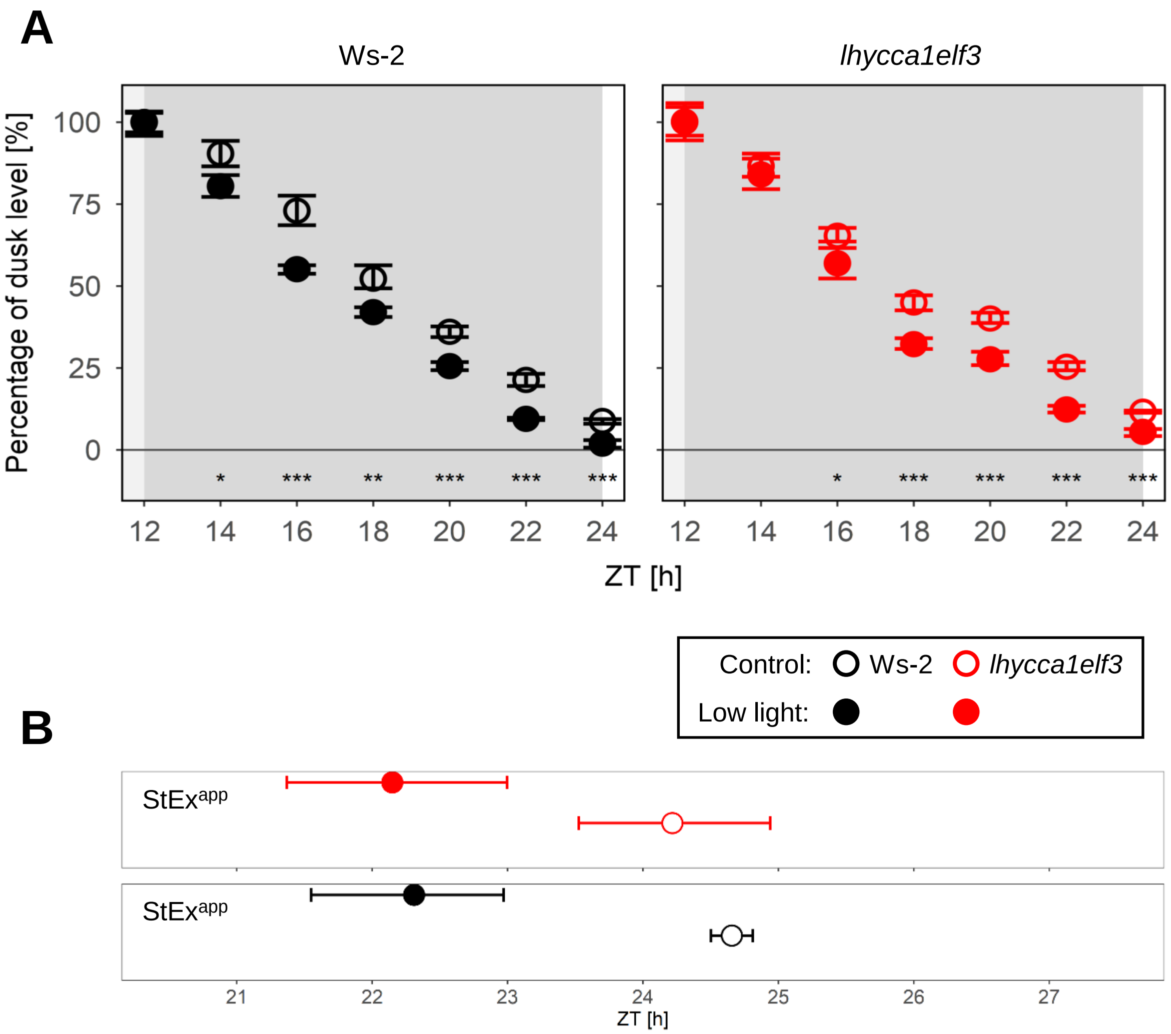

Supplemental Figure S7 – Response of starch mobilization after a sudden early dusk, three further experiments that replicate the experiment of Fig. 4

(A, B, C) Three replicates of the experiment shown in Fig. 4. Their design is given in Suppl. Fig. S1D, except that in one of the experiments (panel A) the plants were harvested during the 22nd day after sowing (DAS) rather than the 14th day after sowing (as in panels B, C, D), and that the density of harvesting differed from experiment to experiment. At each time point, 2 to 7 samples were harvested. Background shading indicates light period (white), the time during which some plants were in the light and others had been already darkened (pale grey) the time when all plants were in darkness (grey). *lhy cca1 elf3* is indicated by red symbols, open symbols denote control conditions and solid symbols denote the early dusk treatment. Symbols represent the mean values, and the error bars indicate bootstrapped 95% confidence interval. Statistical significance (ANOVA, sum of squares type II) is indicated by asterisks (0 '***' 0.001 '**' 0.01 '*' 0.05); subsequent HSD Tukey's post-test is indicated by dashes (i.e. when not significantly different).

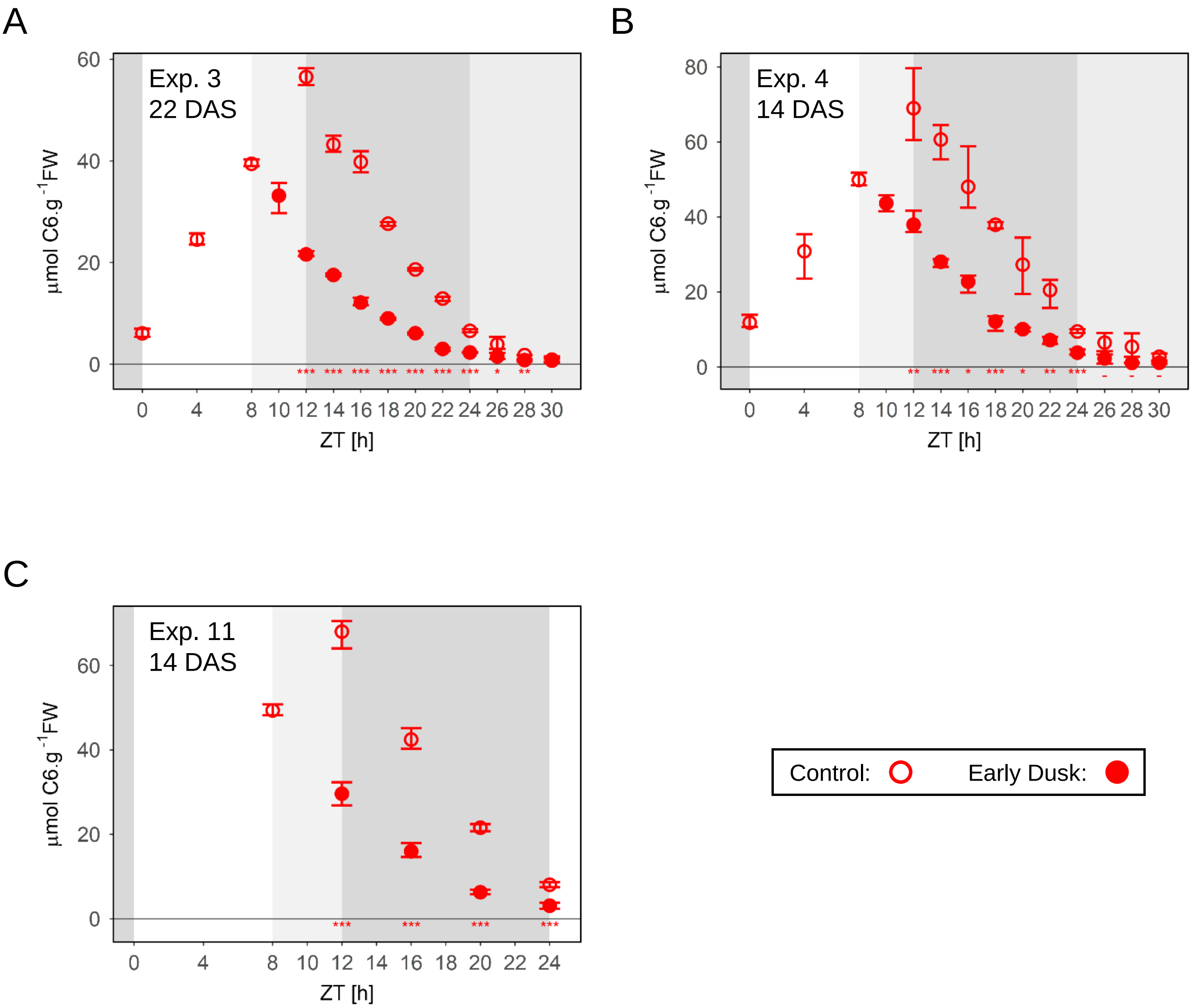

Supplemental Figure S7 – Response of starch mobilization after a sudden early dusk, three further experiments that replicate the experiment of Fig. 4 (continued)

(D) Estimated absolute rates of starch mobilization.

(E) Estimated relative rates of starch mobilization.

Ws-2 and *lhy cca1 elf3* are indicated by black and red symbols, as in panel A. Rates were defined as the slope of linear models, and error bars indicate the 95% confidence interval of the standard error of the slope. Mobilization rates were calculated using time points between ZT12 and ZT24 in the control condition and from ZT8 to ZT18 in the early dusk treatment ("ZT", or "Zeitgeber" from the German language, indicates the time elapsed after the last dawn, in hours). At each time point, 2 to 7 samples were harvested. Solid bars represent the treatment condition whilst the hollow bars indicate control conditions. Relative rates were calculated using starch levels as a proportion of the average starch levels at dusk in each condition. Statistical significance between wild-type Ws-2 and *lhy cca1 elf3* (ANCOVA, Sum of Squares type III) is indicated by asterisks (0 '***' 0.001 '**' 0.01 '*' 0.05). Numeric values are provided in Suppl. Dataset S1.

(F) Estimated time at which starch is exhausted. Symbols represent the projected time of starch exhaustion as defined by the geometric mean of multiple projections ($StEx^{app}$). These were performed using linear models on time spans of varying length that started at dusk and extended for increasing lengths of time into the night (at least three time points) excluding times beyond 8.5 h of darkness, corresponding to ZT16 and ZT20 in the "Control" and the "Early Dusk" conditions, respectively. This allowed comparison over a similar duration after dusk. Error bars indicate the 95% confidence interval of the multiple estimations. Solid and open symbols indicate results from "Early Dusk" and "Control" conditions, respectively. Color indicates different genotypes, as in panel A. Numeric values are provided in Suppl. Dataset S1.

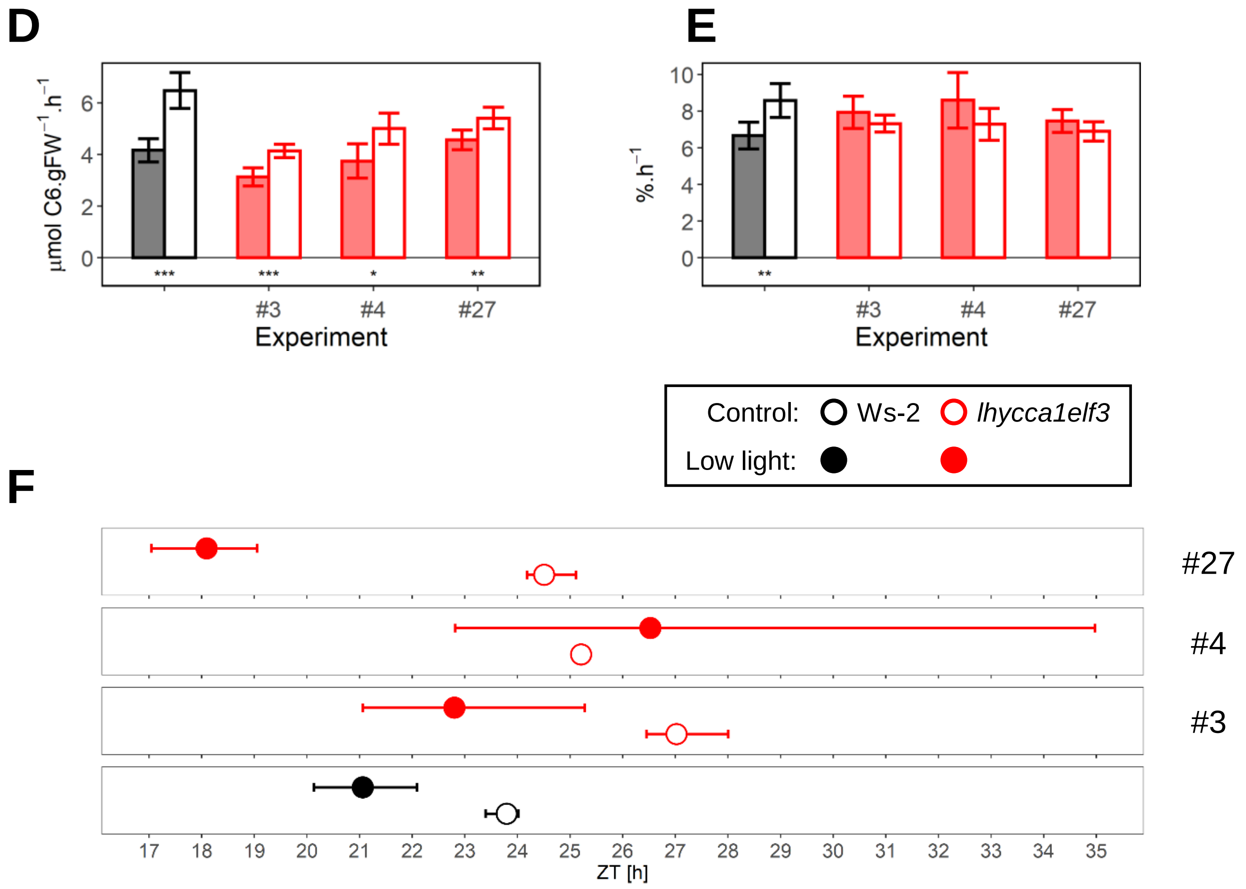

Supplemental Figure S8 – Sugar content

(A) Glucose

(B) Fructose

(C) Sucrose

Each panel shows the response in different growth photoperiod and irradiance (measured in the experiments of Suppl. Fig. S1A and Fig. 1), the response during the night after a sudden drop in irradiance from growth conditions (measured in the experiment of Suppl. Fig. 1C, Fig. 3), the response in the night after a sudden 4-h advance in dusk (right hand subpanel, closed symbols) compared to growth conditions (measured in the experiment of Suppl. Fig. 1D, Fig. 4), and the response in non T24 cycles (measured in the experiment of Suppl. Fig. 1B, Fig. 2).

Background shading indicates light period (white) and night (grey). Wild-type Ws-2 and *lhy cca1* are indicated by black and red symbols, respectively. Background shading indicate light period (white), and night (grey) and (in panel C) the time interval in which some plants had been darkened and control plants were still in the light. Wild-type Ws-2 and *lhy cca1* are indicated by black and red symbols, respectively (see insert). Symbols represent the mean value and the error bars indicate bootstrapped 95% confidence interval. At each time point, 2 to 5 samples were harvested. Statistical significance (ANOVA, sum of squares type II) is indicated by asterisks (0 '***' 0.001 '**' 0.01 '*' 0.05; subsequent HSD Tukey's post-test is indicated by dashes when not significantly different). NA denotes that a test was not applicable due to lack of replicates. "ZT", or "Zeitgeber" from the German language, indicates the time elapsed after the last dawn, in hours.

Supplemental Figure S8 – Sugar content (continued)

A Glucose

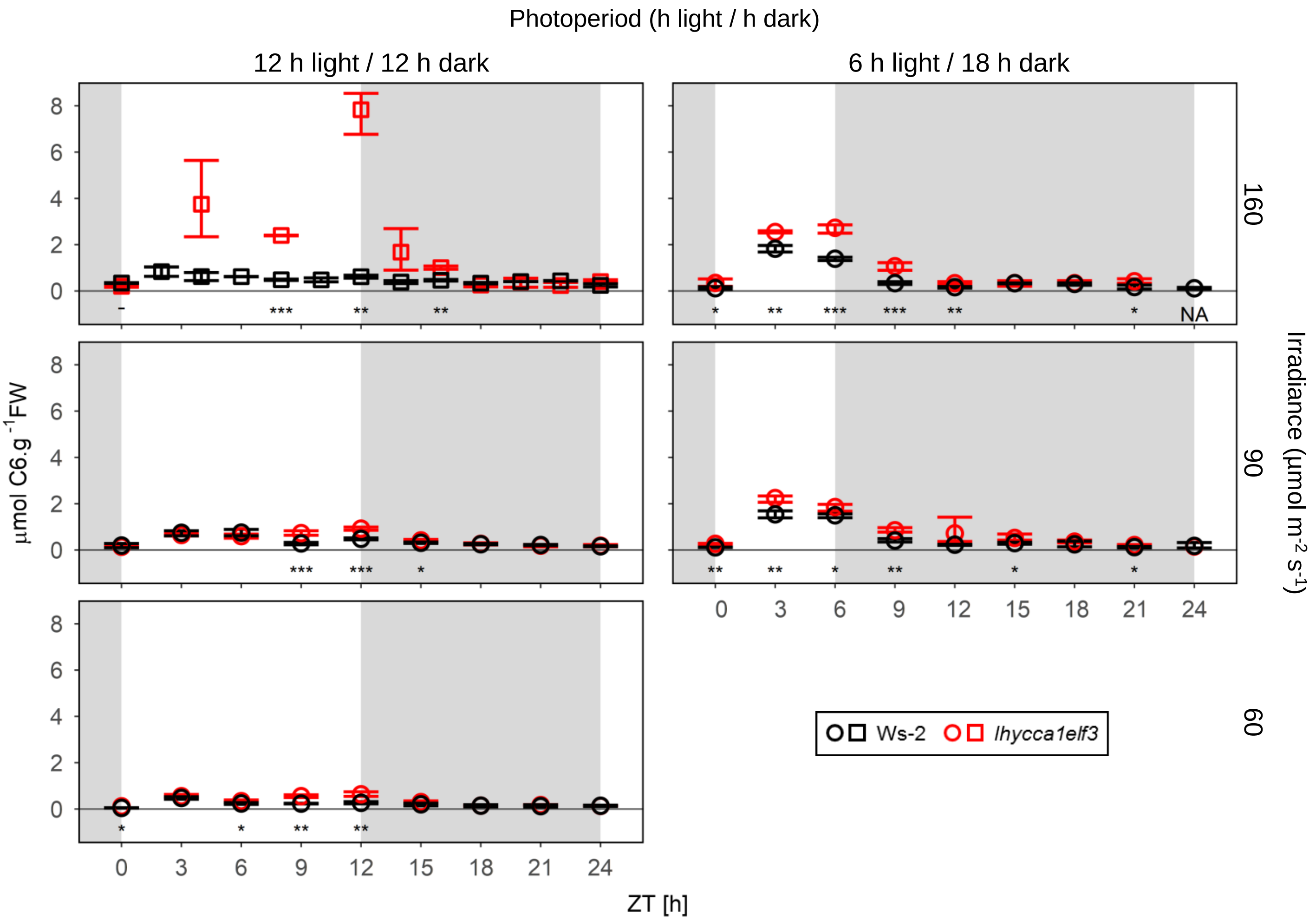

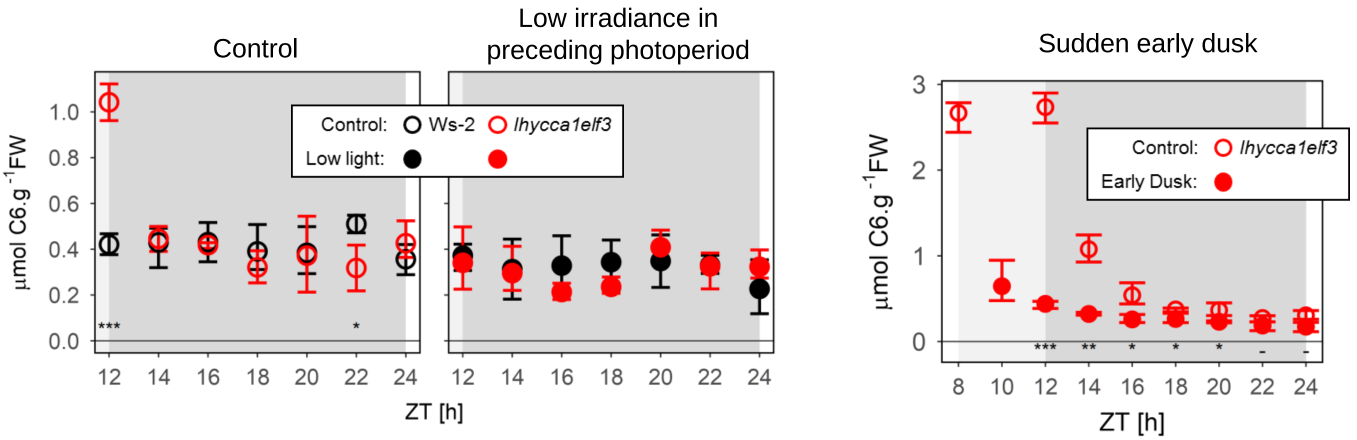

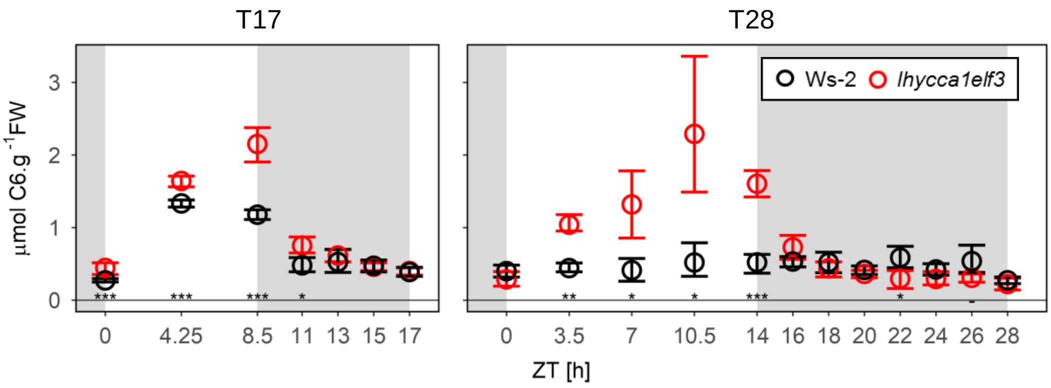

Supplemental Figure S8 – Sugar content (continued)

B Fructose

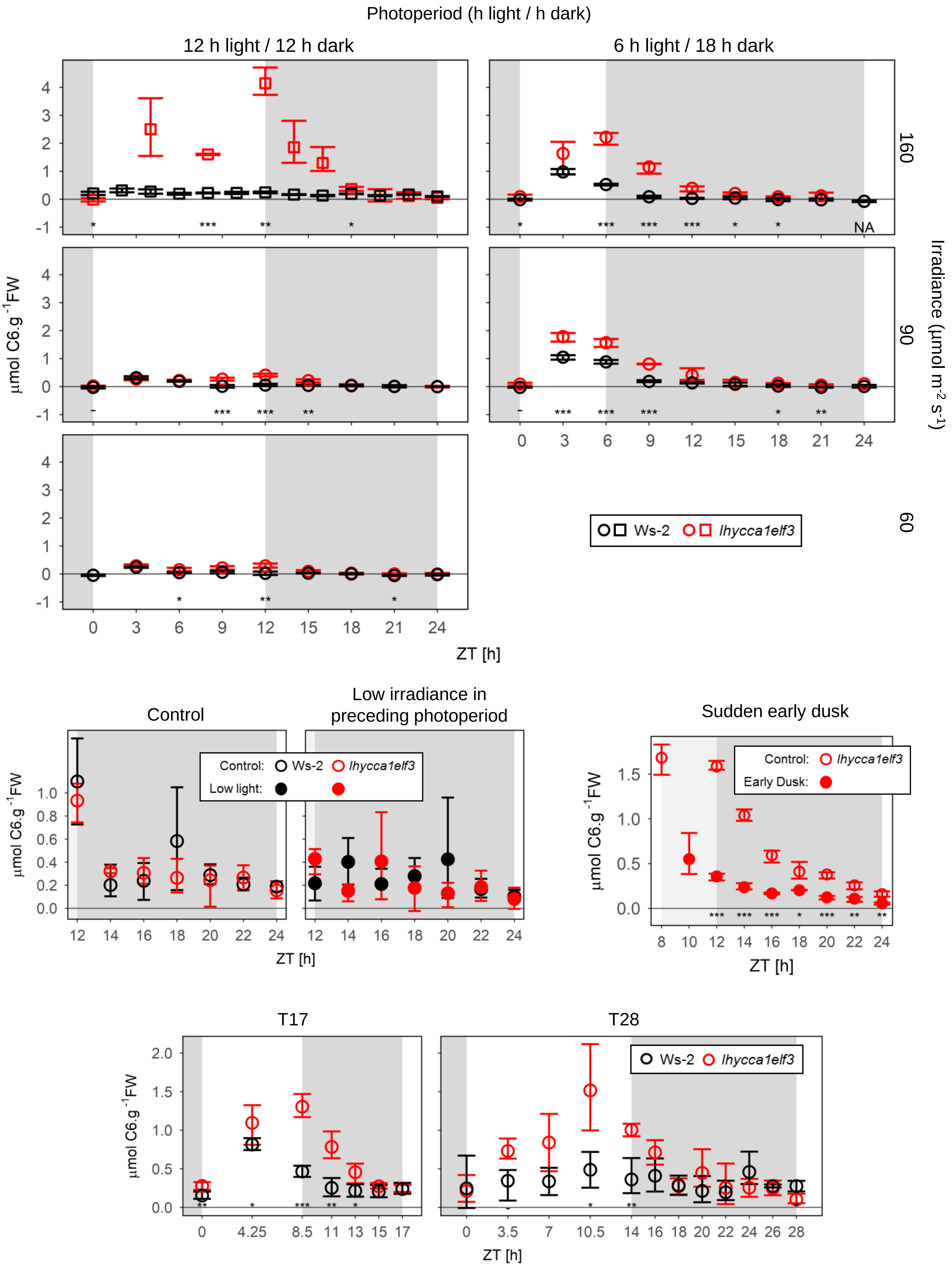

Supplemental Figure S8 – Sugar content (continued)

C Sucrose

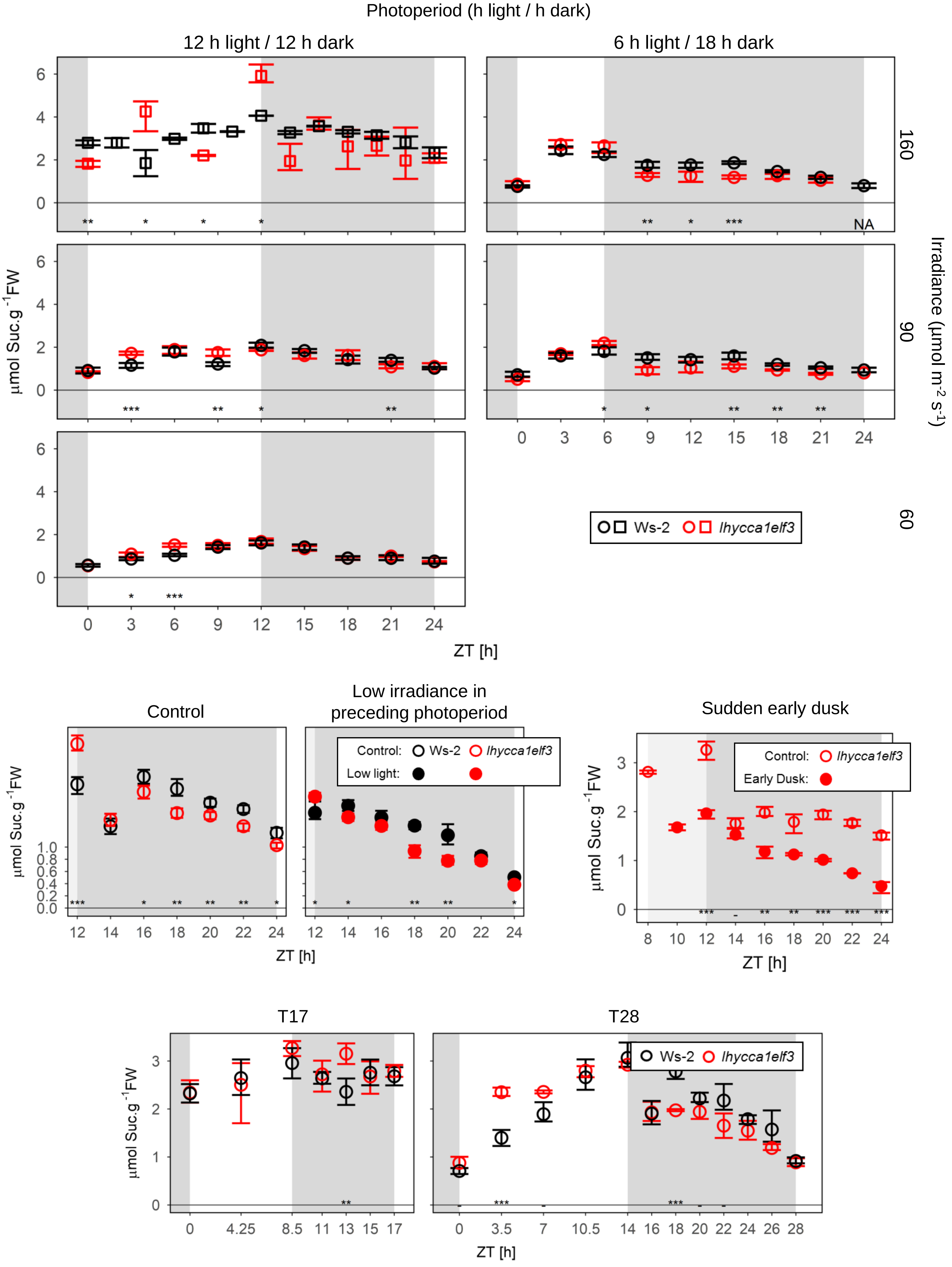

Supplemental Figure S9. Diel response of transcript abundance in different photoperiods

(A) Clock transcripts

(B) *RVE* family members

(C) *PIF* family members and *GBSS1*

Transcripts were measured in the 12-h photoperiod / 160 µmol m^{-2} s^{-1} irradiance experiment of Suppl. Fig. S1A (the experiment used to measure starch and sugars in this condition, Fig. 1, Fig, 6, Suppl. Fig. S8) and in the 6-h and 18-h photoperiod (both a 160 µmol m^{-2} s^{-1} irradiance) experiments of Suppl. Fig. S1E (there was insufficient material to measure transcripts in the 6-h photoperiod experiment from Suppl. Fig. S1A). At each time point, 2 to 5 samples were harvested. Transcript abundance was measured by RT-qPCR, adding artificial RNA standard before cDNA amplification to allow absolute quantification. Abundance is given as log_2(copies x 2.5 $x10^7$ / g FW). Selected transcripts (*PPR9*, *PPR7*, *RVE8*, *GBSS1*) are also shown in Fig. 8.

For each transcript the display block shows the diel response in a 6-h (top), 12-h (middle) and 18-h (bottom) photoperiod. The time series were aligned either to dawn (left hand displays) or to dusk (right hand displays), with the numbers giving the hours after dawn or the hours after dusk, respectively. The background shading indicates the light period (white) or night (grey). Wild-type Ws-2 and *lhy cca1 elf3* are indicated by black and red symbols, respectively (see insert). The symbols give the mean value, and error bars indicate the bootstrapped 95% confidence interval. Statistical significance (ANOVA, sum of squares type II) is indicated by asterisks (0 '***' 0.001 '**' 0.01 '*' 0.05); subsequent HSD Tukey's post-test is indicated by dashes (i.e. when not significantly different).

Supplemental Figure S9. Diel response of transcript abundance in different photoperiods

A Clock transcripts (continued on next page)

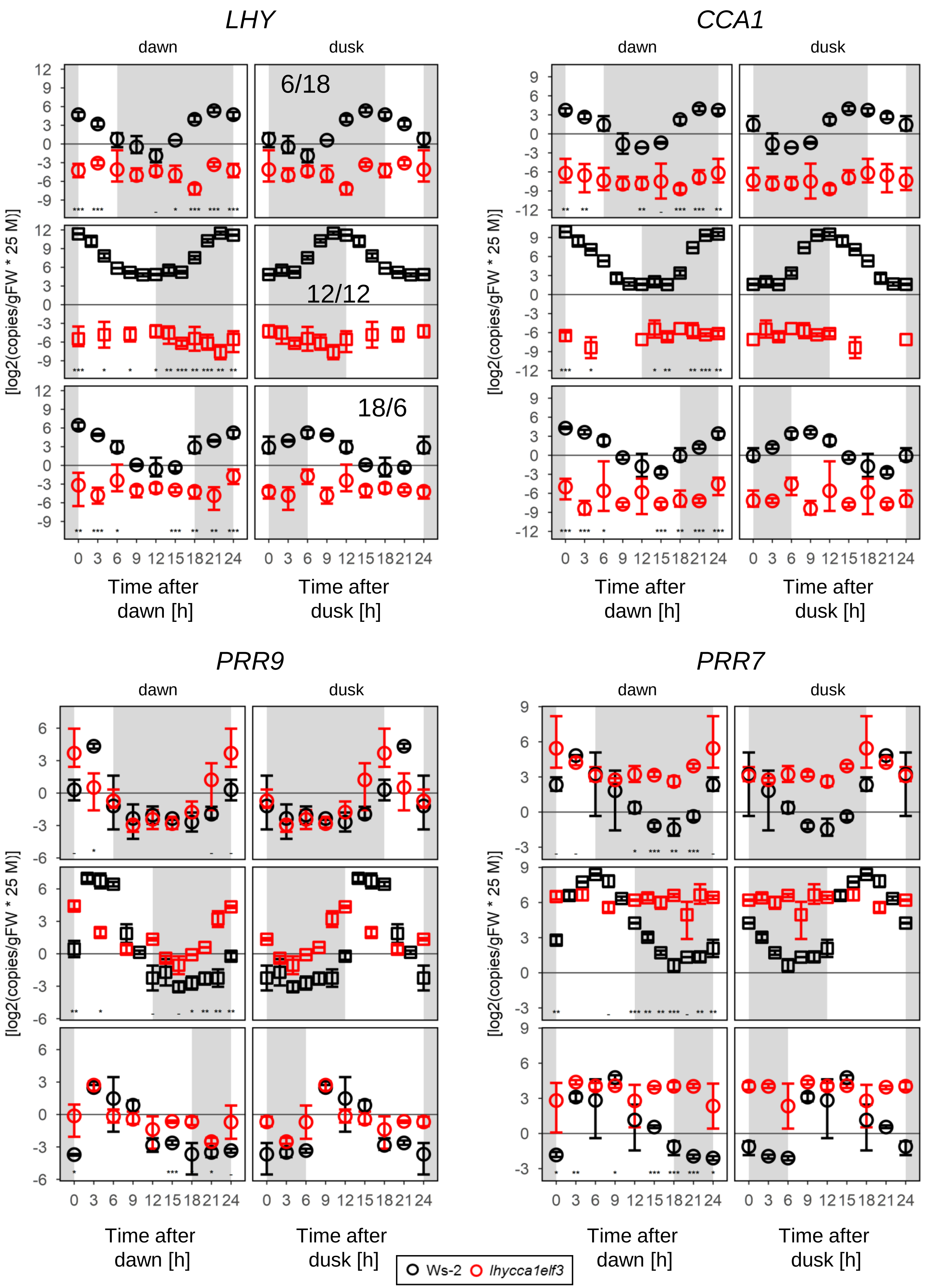

Supplemental Figure S9. Diel response of transcript abundance in different photoperiods

A Clock transcripts (continued)

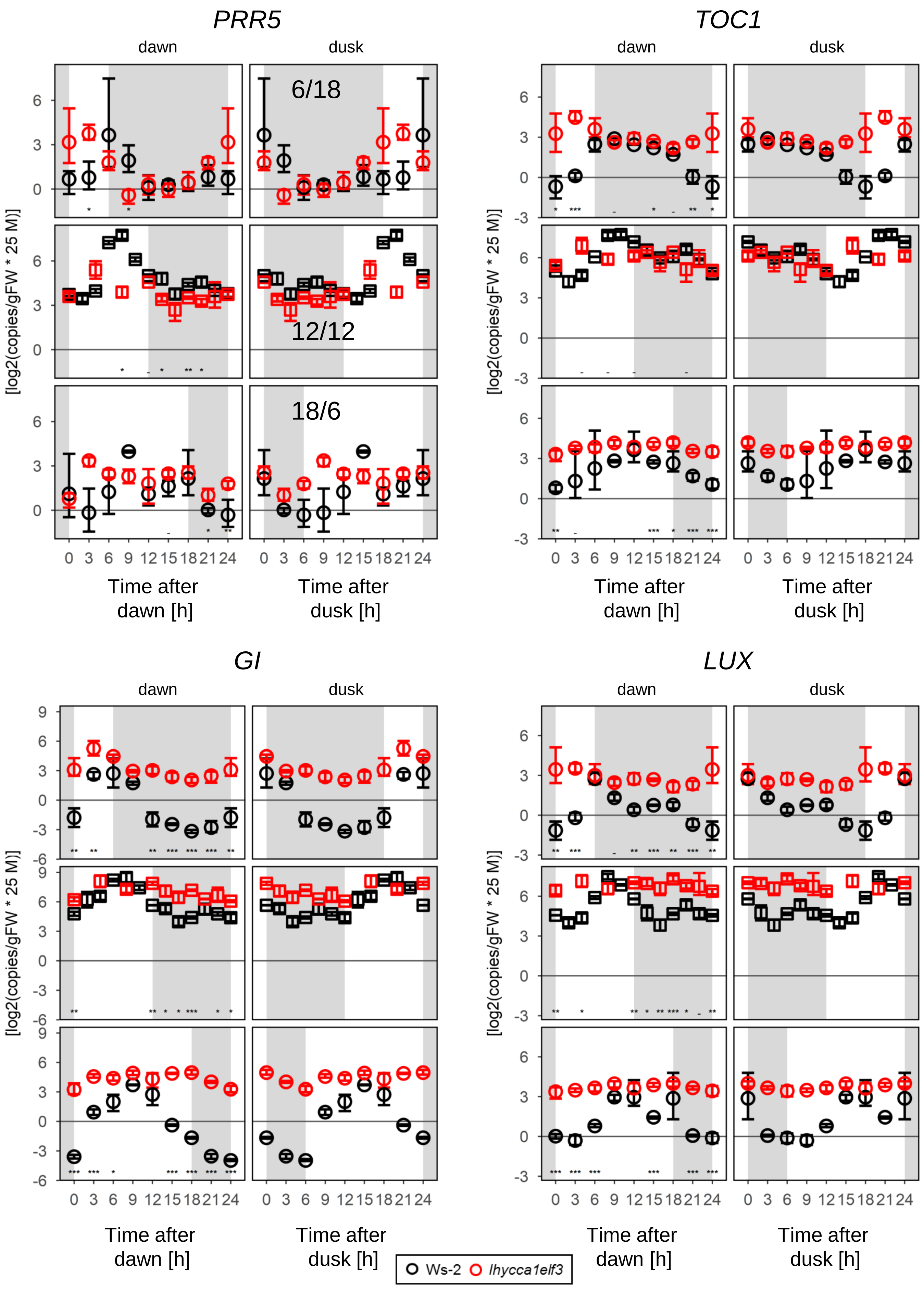

Supplemental Figure S9. Diel response of transcript abundance in different photoperiods

A Clock transcripts (continued)

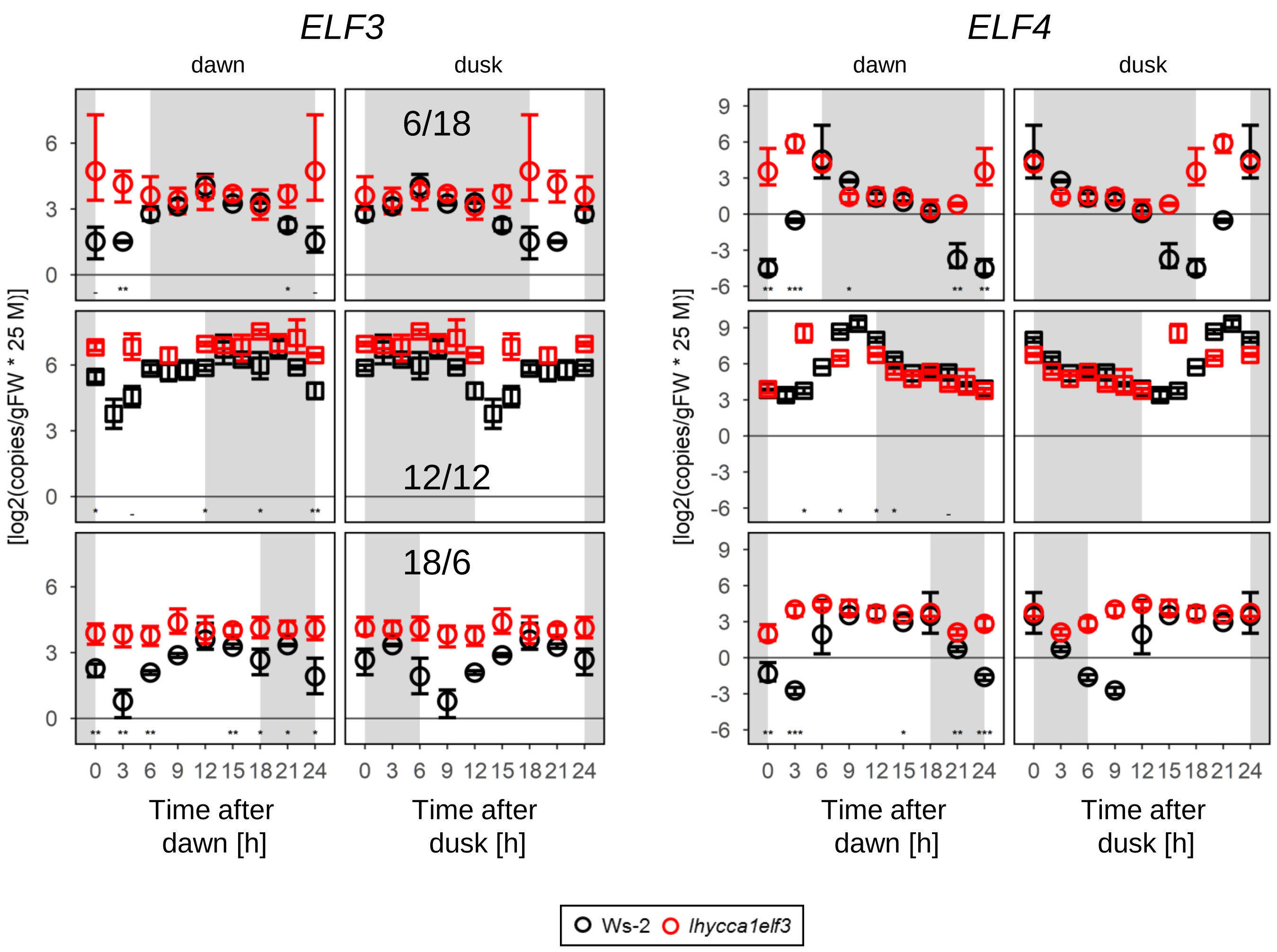

Supplemental Figure S9. Diel response of transcript abundance in different photoperiods

B *RVE* family members (continued on next page)

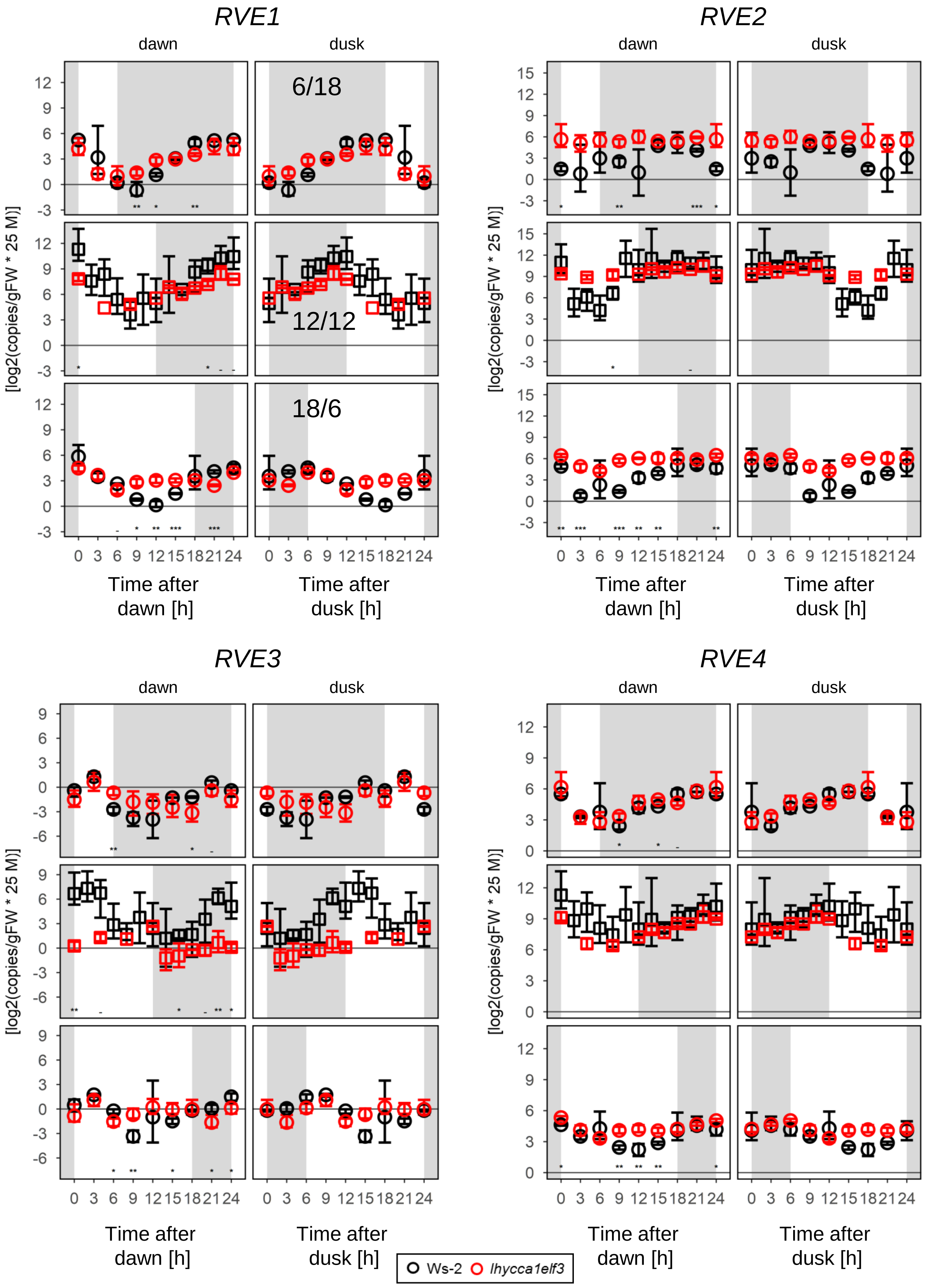

Supplemental Figure S9. Diel response of transcript abundance in different photoperiods

B *RVE* family members (continued)

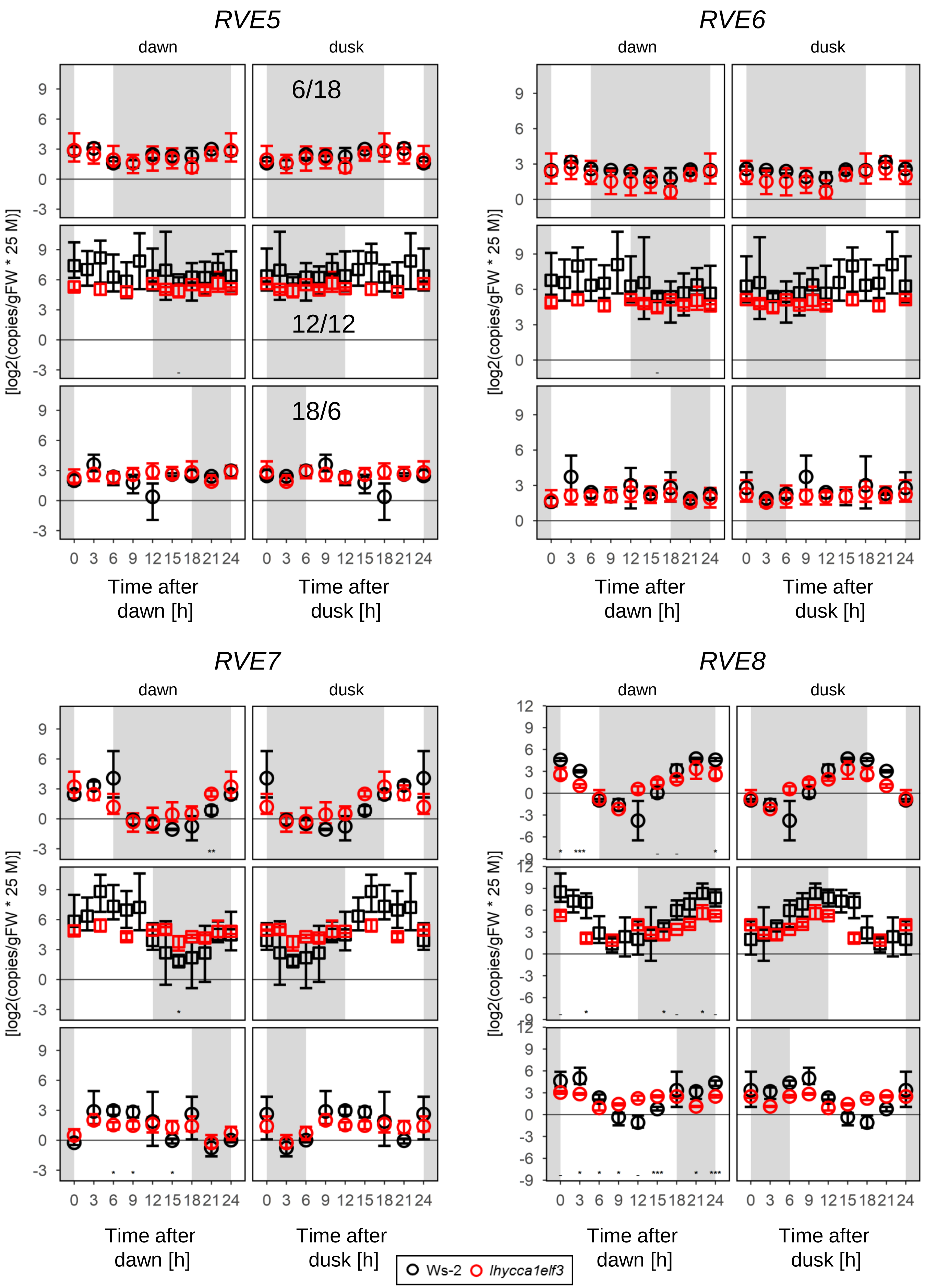

Supplemental Figure S9. Diel response of transcript abundance in different photoperiods

C *PIF* family members *and GBSS1* (continued on next page)

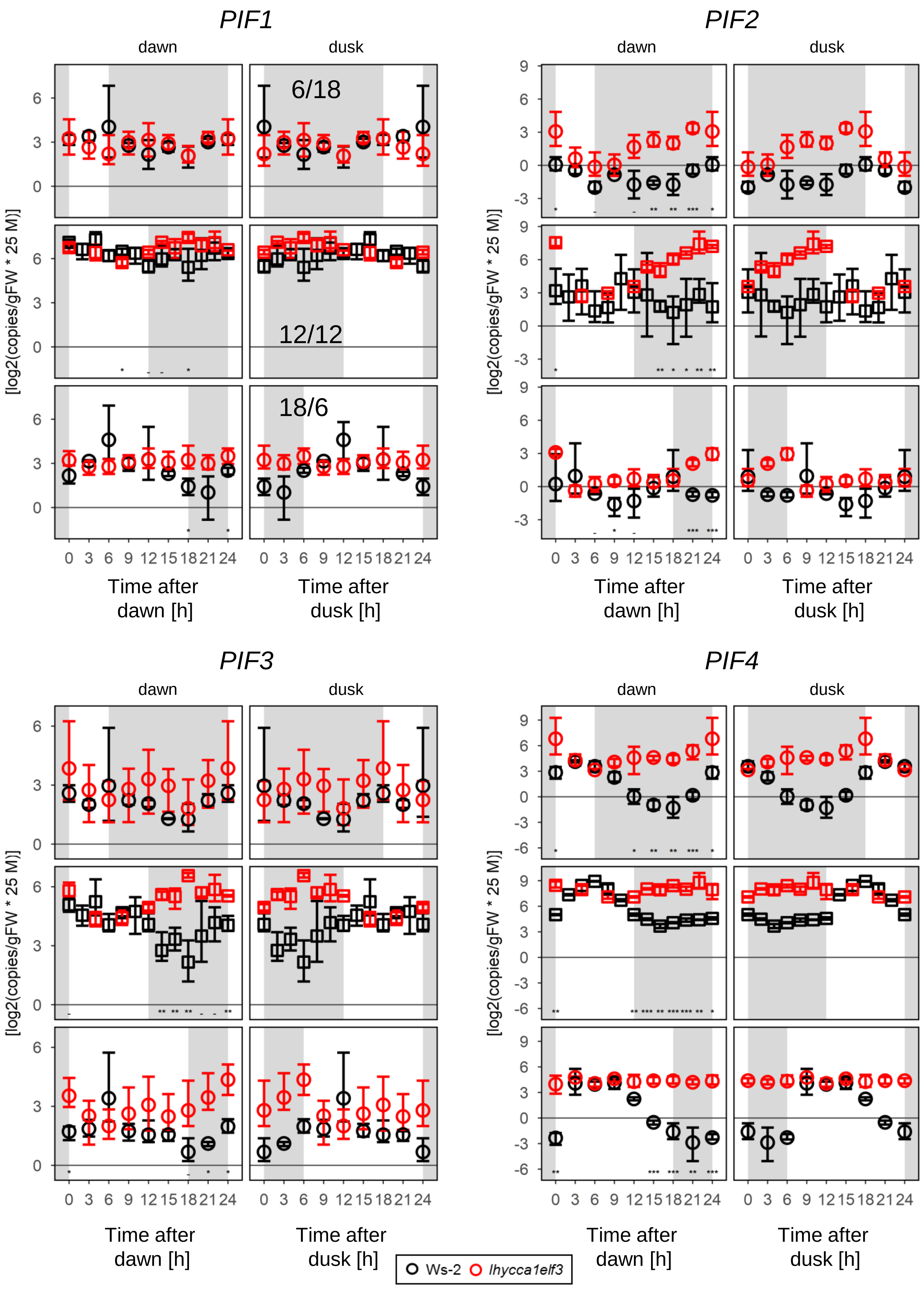

Supplemental Figure S9. Diel response of transcript abundance in different photoperiods

C *PIF* family members *and GBSS1* (continued)

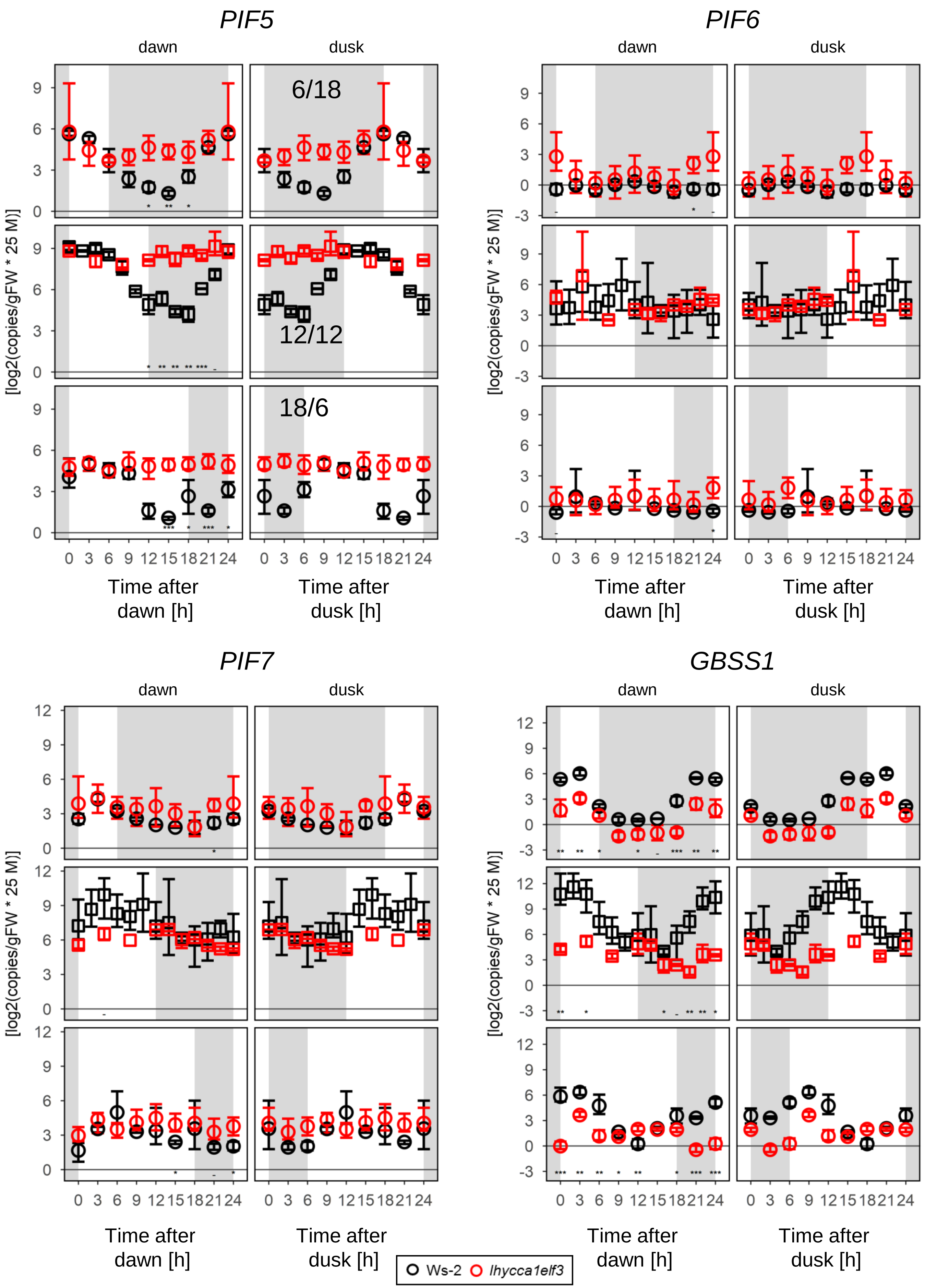

Supplemental Figure S10. Two-way ANOVA test of whether the oscillations of transcript abundance in different photoperiods and after a sudden early dusk are dawn-or dusk-aligned in Ws-2 and *lhy cca1 elf3*

Two-way ANOVA was performed to test two null hypotheses: a) that time series are significantly different (i.e. desynchronized) when they are aligned to dawn, and b) that time series are significantly different when they are aligned to dusk (for details of the analyses and numeric values see Suppl. Table S2). In contrast to the mutual information analysis in Fig. 9, two-way ANOVA cannot be performed on extrapolated values. The analysis is therefore restricted to the 6 h light / 18 h dark and18 h light / 6 h time series (Suppl. Figs. S9 and S11) for which identical sampling times at 3 h intervals were available. At each time point, 2 to 7 samples were harvested.

For a given transcript, the diel oscillations in a 6-h, 12-h and 18-h photoperiod were aligned to either dawn or dusk (for plots, see Suppl. Fig. S9; to generate the dusk alignment, each 24 h time series was centered, then duplicated and concatenated, shifted to a dusk alignment and cropped back to 24 h). Two-way ANOVA was performed to test a) if the 6-h and 18-h photoperiod time series are significantly different when they were aligned to dawn and b) if the 6-h and 18-h photoperiod time series are significantly different when they were aligned to dusk. Tests were performed and reported separately for each transcript. Horizontally, the left-hand block reports the results for *lhy cca1 elf3*, and the right-hand block reports the results for wild-type Ws-2. Within each block, the left-hand column reports the results for the dawn-aligned time series and the right-hand column reports the results for the dusk-aligned time series. Vertically, the top block reports on transcripts for core clock genes, the middle block reports on transcripts for the *RVE* gene family, and the lower block reports on transcripts for the *PIF* gene family and *GBSS1*.

The reported p-values are shown as a heat map, with black depicting strong p-values approaching 0 (i.e., the null hypothesis is accepted: the time series are desynchronized) and increasingly weak p-values as grey (i.e. the null hypothesis is not accepted but is also not strongly rejected: the time series may be somewhat desynchronized) to pale grey or white (i.e., the null hypothesis is rejected; the time series are synchronized). Thus, in panel A for Ws-2, the solid block of white for dawn-aligned core clock transcripts and the near-solid block of grey for dusk-aligned core clock transcripts reflects the known dawn-dominance of the wild-type clock. In *lhy cca1 elf3*, many core clock genes, *RVE8* and *GBSS1* are reported as being partly (or for *PRR7*, almost totally) desynchronized (i.e. not dawn dominant), whereas the hypothesis that most of the genes are not dusk dominant is rejected. This points to a weakening of dawn dominance in the triple mutant.

Numerical results are provided in Suppl. Dataset S5.

Supplemental Figure S10 – Two-way ANOVA test of whether the oscillations of transcript abundance in different photoperiods and after a sudden early dusk are dawn-or dusk-aligned in Ws-2 and *lhy cca1 elf3*

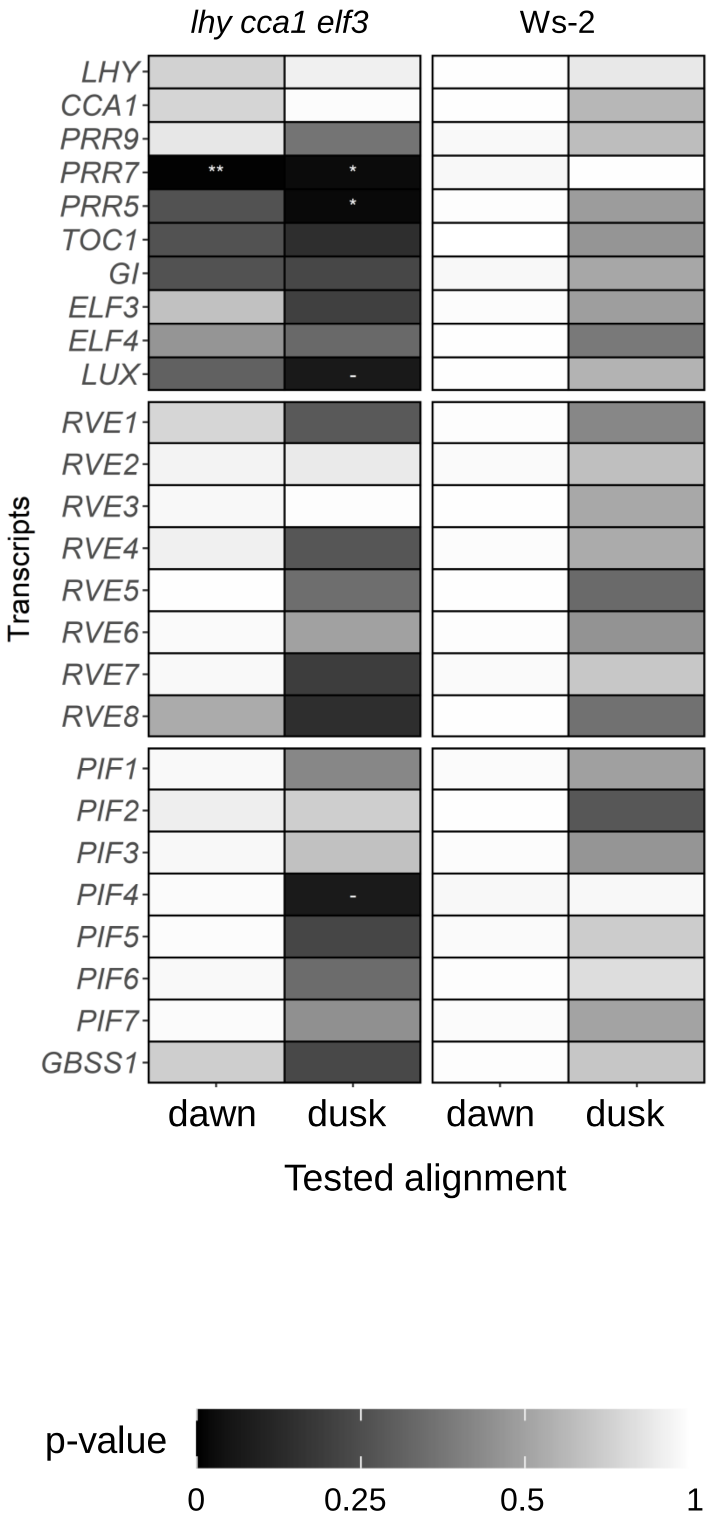

Supplemental Figure S11. Response of transcript abundance to a sudden early dusk

(A) Clock, (B) *RVE* family member and (C) *PIF* family member and *GBSS1* transcript abundance. Transcripts were measured in the experiment of Suppl. Fig. S1D in the same material that was used to measure starch for Fig. 4. Transcript abundance was measured by RT-qPCR, adding artificial RNA standard before cDNA amplification to allow absolute quantification. Abundance is given as $\log_2$(copies x 2.5 x10^7 / g FW). At each time point, 2 to 3 samples were harvested. Shading indicates light period (white), the time interval in which some plants had been darkened and control plants were still in the light (pale grey) and night (grey). Wild-type Ws-2 and *lhy cca1* are indicated by black and red symbols, respectively (see insert). Closed symbols denote the response after a sudden 4 h advance in dusk, and open symbols the response in control plants left in growth conditions. The symbols give the mean value, and error bars indicate the bootstrapped 95% confidence interval. Statistical significance (ANOVA, sum of squares type II) was performed for each genotype separately is indicated by colored asterisks (0 '***' 0.001 '**' 0.01 '*' 0.05); subsequent HSD Tukey's post-test is indicated by dashes (i.e. when not significantly different). "ZT", or "Zeitgeber" from the German language, indicates the time elapsed after the last dawn, in hours.

(D) Comparison of control and sudden early dusk using mutual information analysis. Analyses for Ws-2 are shown in the left hand and for *lhy cca1 elf3* in the right-hand block of plots. The analysis was performed in an analogous manner to that in Fig. 9. For each genotype and transcript, the transcript time series data were organized as "dawn aligned" (i.e. profiles starting at light on) and as "dusk aligned" (i.e. profiles starting at the time at which the plants were darkened; all transcript time series were advanced by 4 h for the sudden early dusk). The DIST score is defined as the Euclidean Distance between all treatments. The NORM score is defined as the Euclidean Distance divided by its propagated error (see Suppl. Dataset S4 for details). Heatmap colors indicate the average value of each score for each transcript; as explained in Suppl. Dataset S4. DIST and NORM scores were independently transformed to values between 0 and 1, with 0 indicating the best and 1 the worst alignment observed. ANOVA (Sum of Squares type II) and subsequent Tukey's HSD was used to detect which transcripts were not significantly different between "dawn" and "dusk"; these are indicated as grey. Missing values are indicated by NA. At each time point, 2 to 3 samples were harvested.

Supplemental Figure S11. Response of transcript abundance to a sudden early dusk (continued)

A Clock transcripts

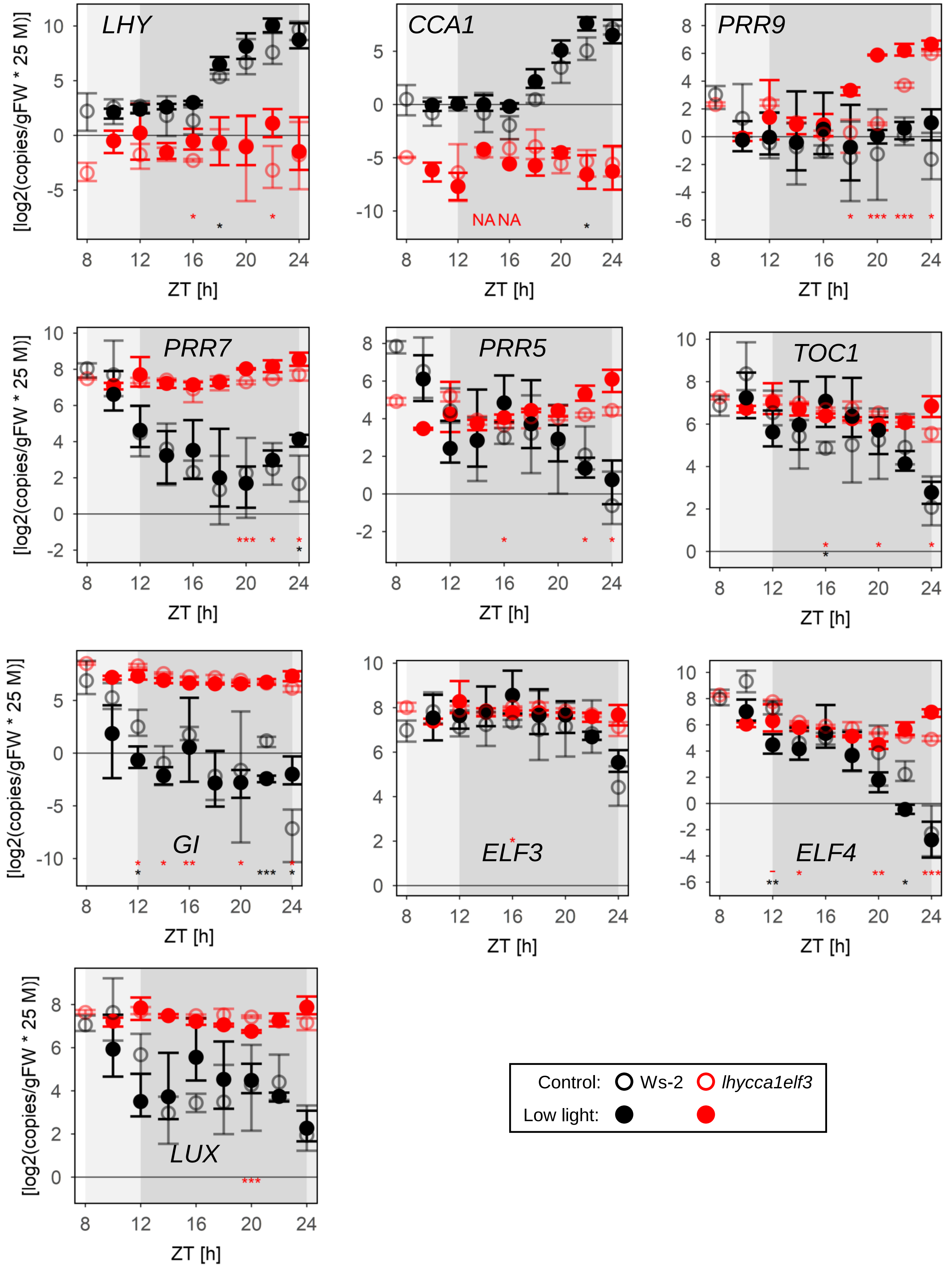

Supplemental Figure S11. Response of transcript abundance to a sudden early dusk (continued)

B ***RVE* family members**

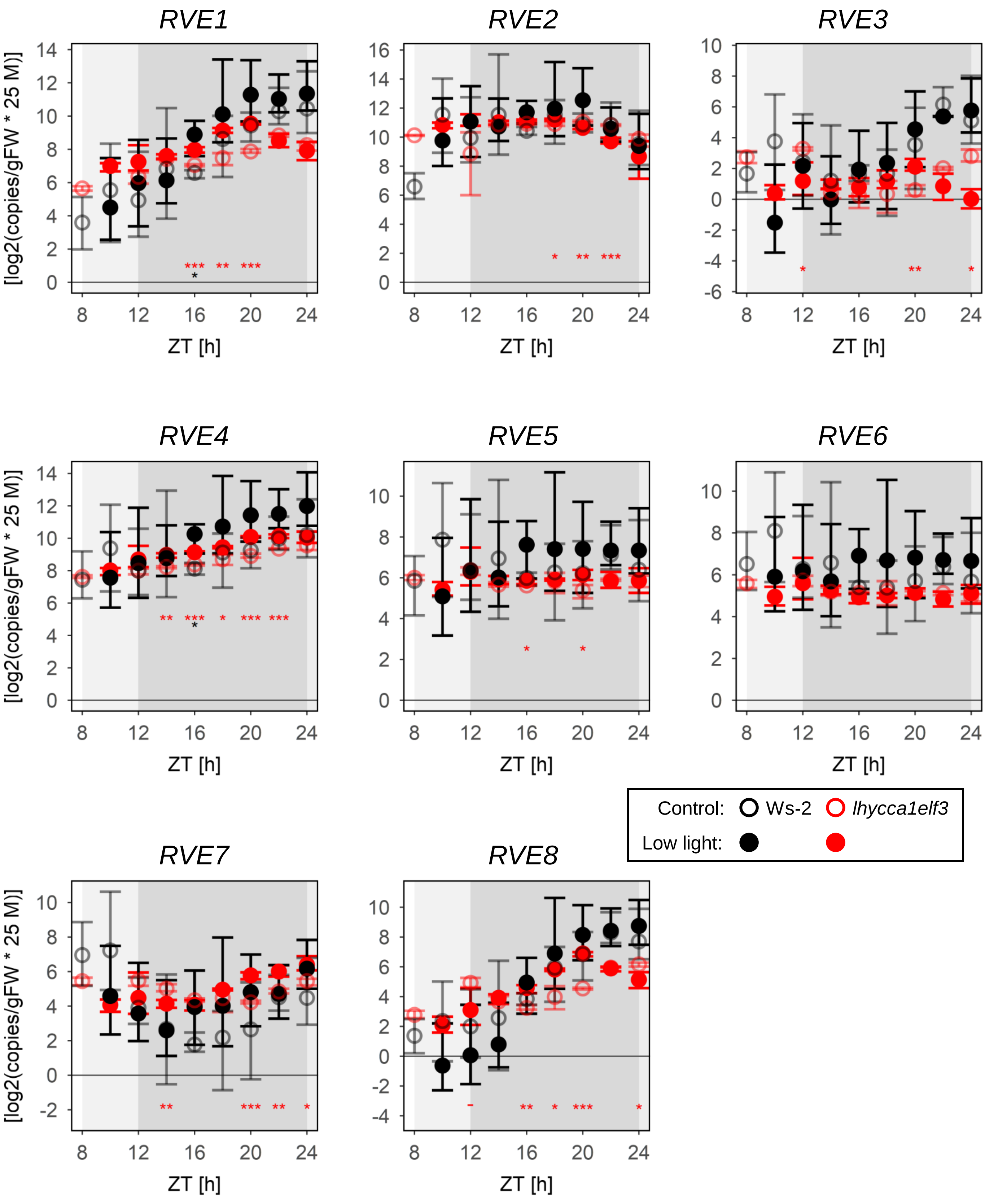

Supplemental Figure S11. Response of transcript abundance to a sudden early dusk (continued)

C ***PIF* family members *and GBSS1***

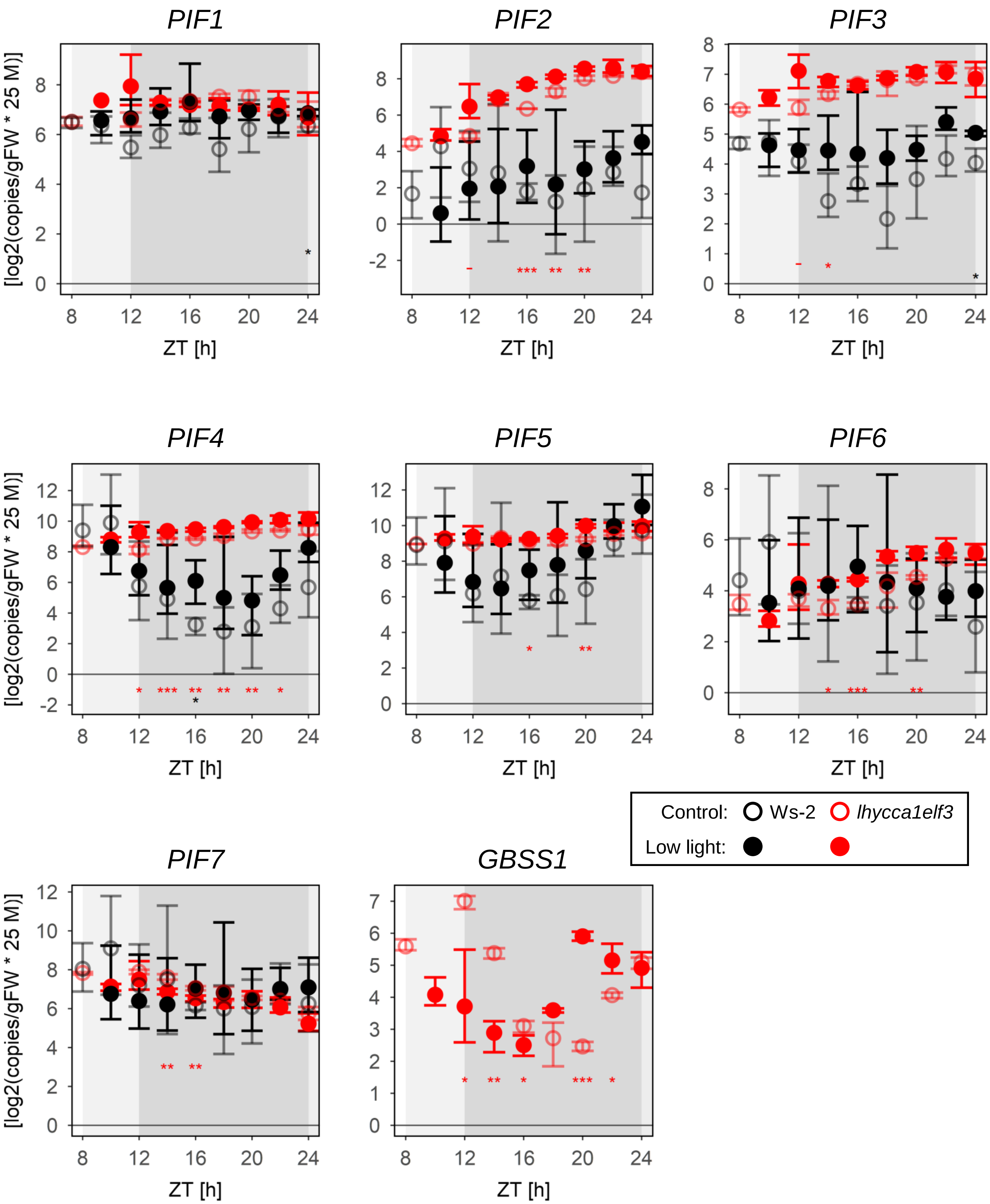

Supplemental Figure S11. Response of transcript abundance to a sudden early dusk (continued)

D **Comparison of control and sudden early dusk using mutual information analysis**

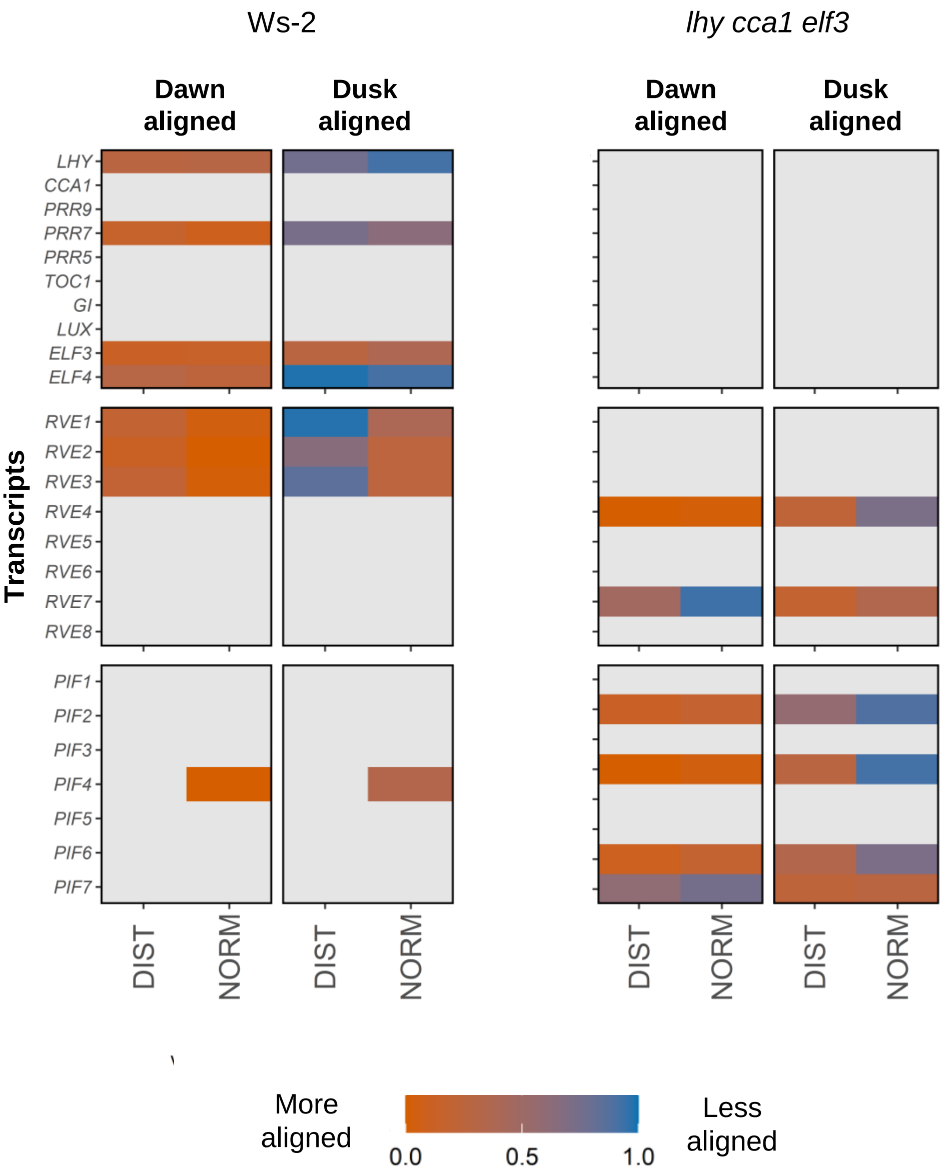

Supplemental Figure S12. Response of transcript abundance in different T cycles

Transcripts were measured for the T17 and T28 cycles in the experiment of Suppl. Fig. S1B in the same material that was used to measure starch for Fig. 2, and for the T24 cycle in the experiment of Suppl. Fig. S1A (at 160 µmol m^{-2} s^{-1} irradiance) in the same experiment as was used to measure starch for Fig. 1. At each time point, 2 to 5 samples were harvested. Transcript abundance was measured by RT-qPCR, adding artificial RNA standard before cDNA amplification to allow absolute quantification. Abundance is given as log_2(copies x 2.5 x10^7 / g FW).

(A) Clock transcripts

(B) *RVE* family members

(C) *PIF* family members and *GBSS1*

(D) Carbon starvation reporters (data for the 12 h treatment is also shown in Fig. 6).

For each transcript, the display block shows the diel response in an 8.5 h light / 8.5 h dark (T17, top panel), a 12 h light / 12 h dark (T24, middle panel) and 14 h light / 14 h dark (T28, bottom panel). The time series were aligned to dawn. The background shading indicates the light period (white) or night (grey). Wild-type Ws-2 and *lhy cca1 elf3* are shown as black and red symbols, respectively (see insert). Pale black and red symbols indicate data points that were duplicated and concatenated after the experimental period (truncated to 28 h time series). The symbols give the mean value, and error bars indicate the bootstrapped 95% confidence interval. Statistical significance (ANOVA, sum of squares type II) is indicated by asterisks (0 '***' 0.001 '**' 0.01 '*' 0.05; subsequent HSD Tukey's post-test was significant in all cases). "ZT", or "Zeitgeber" from the German language, indicates the time elapsed after the last dawn, in hours.

Supplemental Figure S12. Response of transcript abundance in different T cycles (continued)

A Clock transcripts (continued on next page)

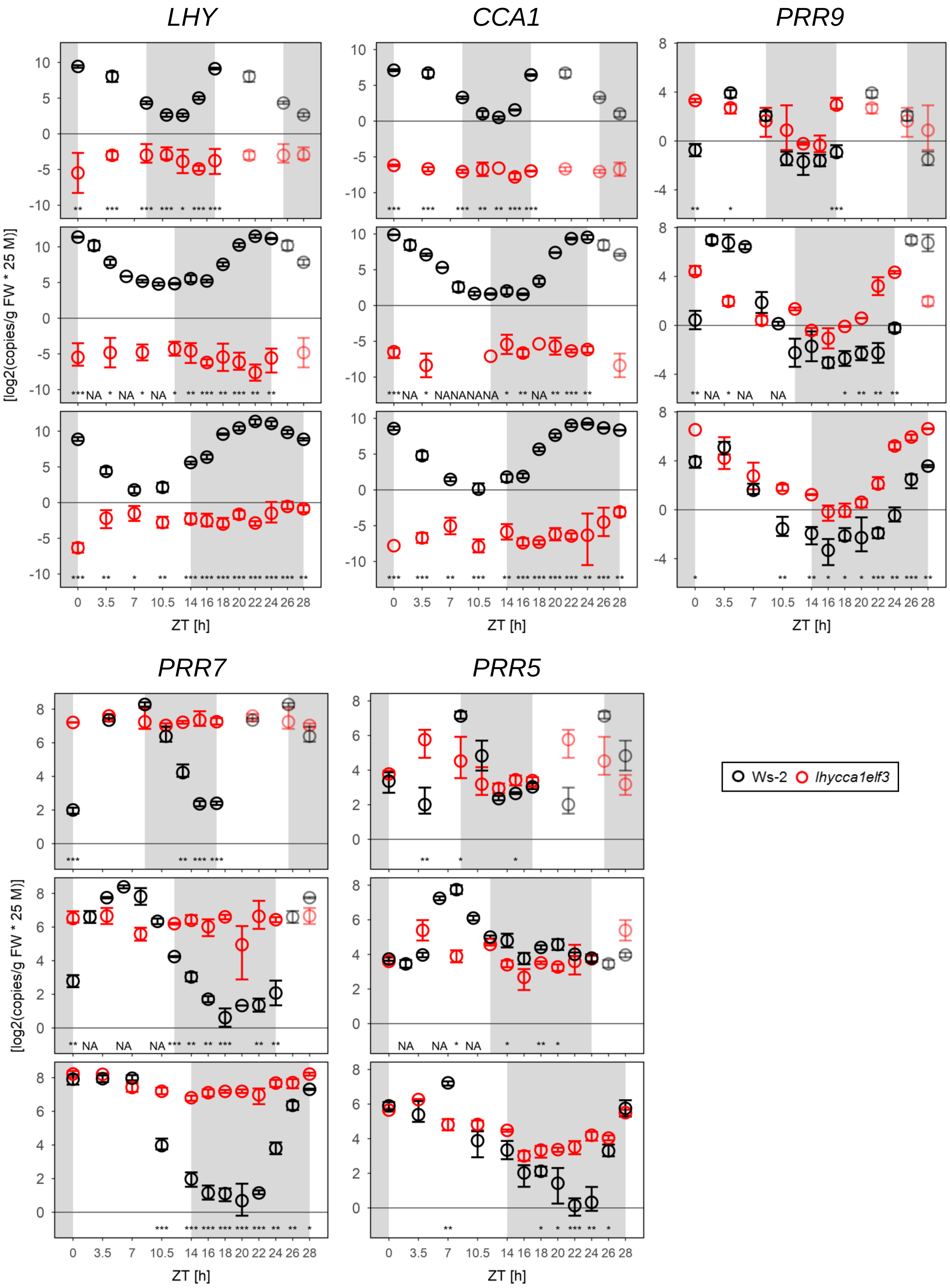

Supplemental Figure S12. Response of transcript abundance in different T cycles (continued)

A Clock transcripts (continued on next page)

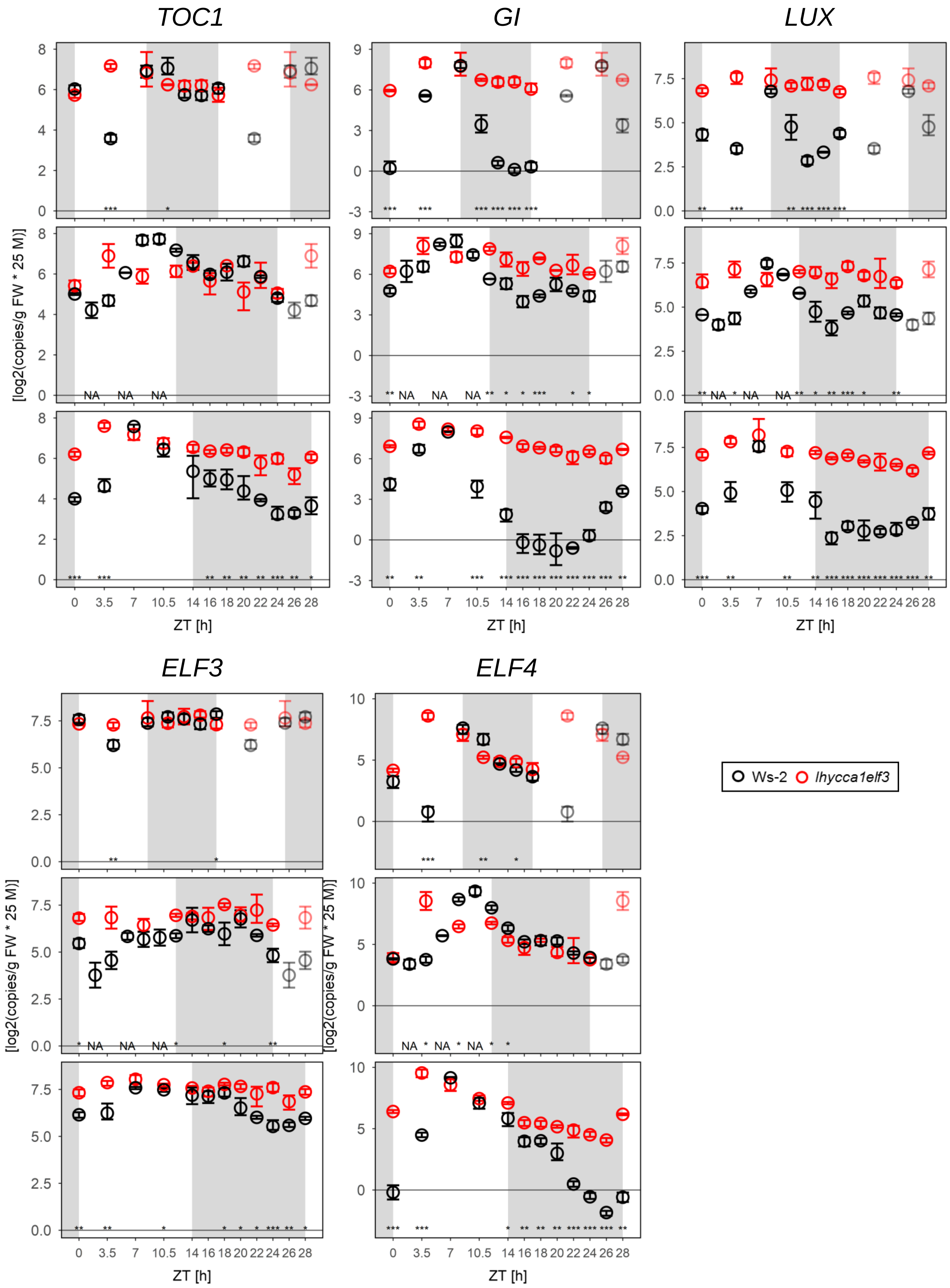

Supplemental Figure S12. Response of transcript abundance in different T cycles (continued)

B ***RVE*** **family members (continued on next page)**

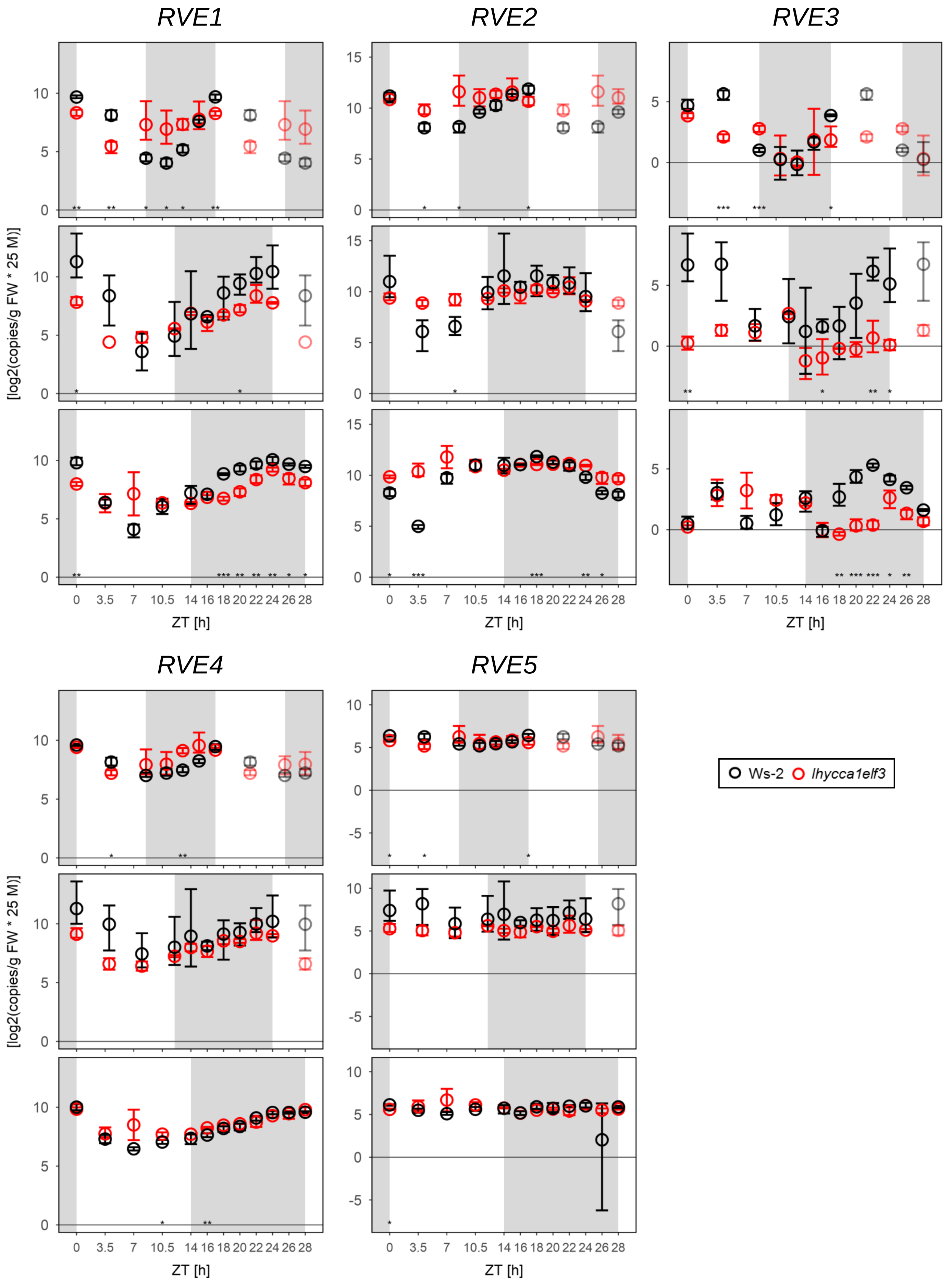

Supplemental Figure S12. Response of transcript abundance in different T cycles (continued)

B *RVE* family members (continued)

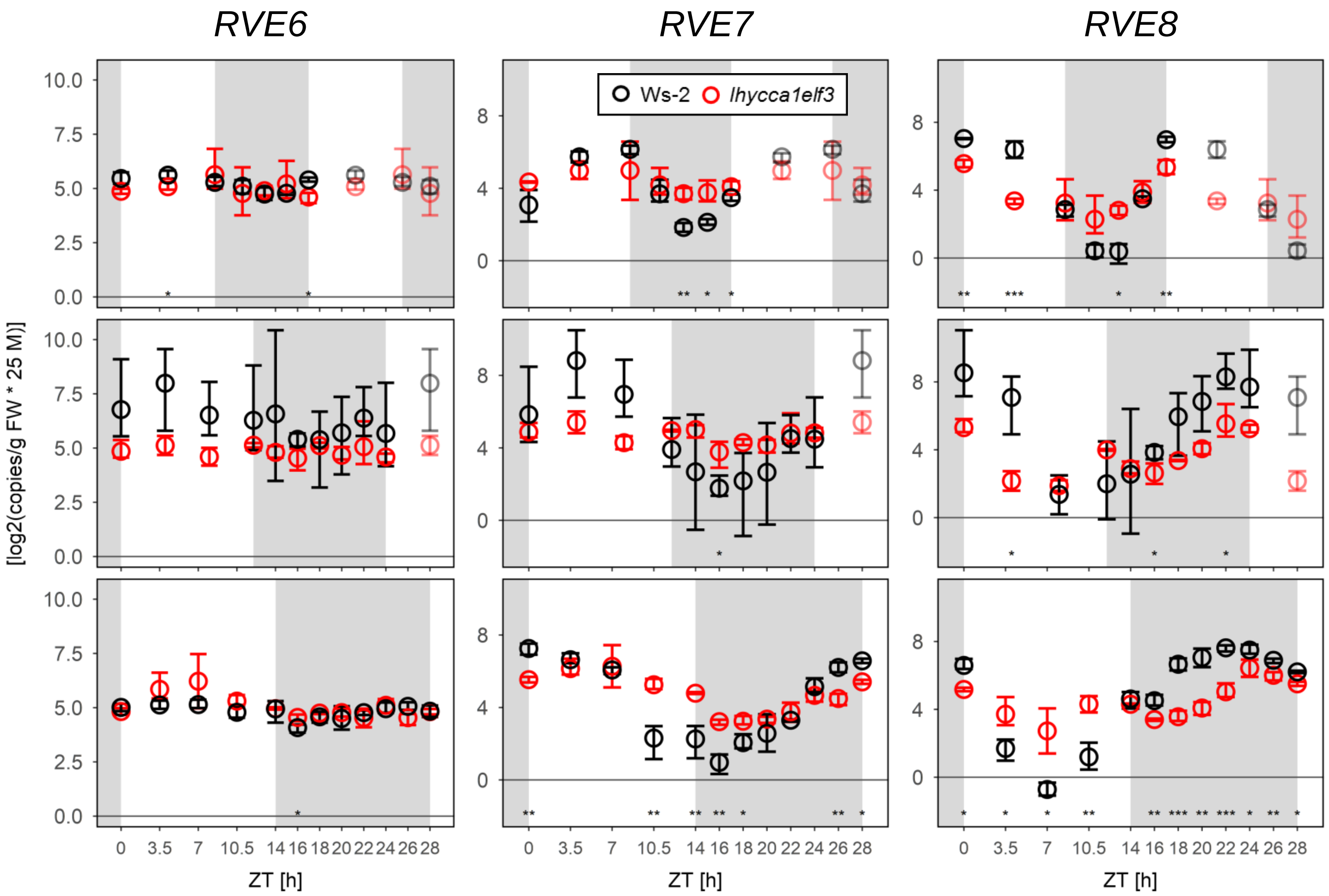

Supplemental Figure S12. Response of transcript abundance in different T cycles (continued)

C *PIF* family members and *GBSS1* (continued on next page)

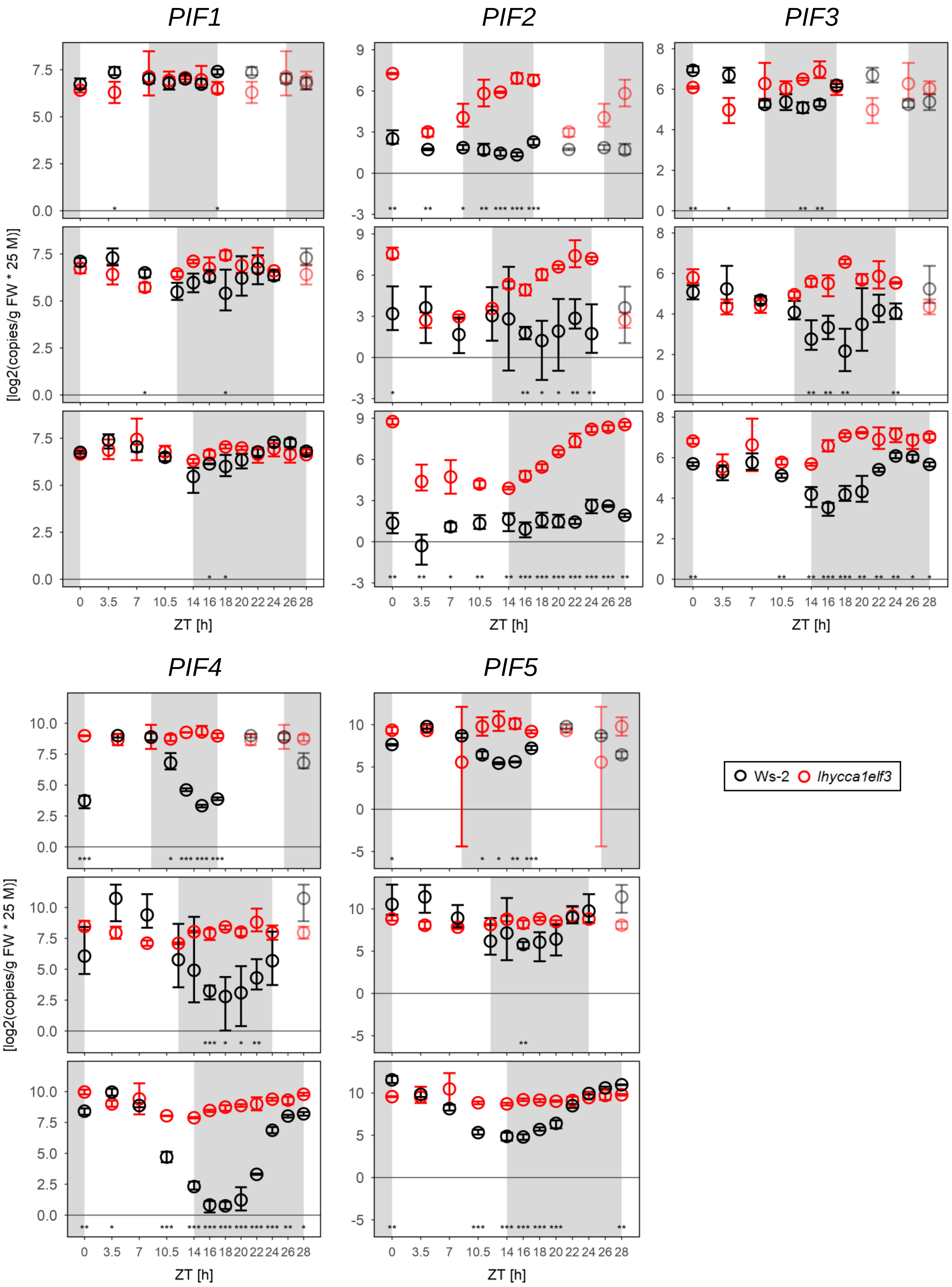

Supplemental Figure S12. Response of transcript abundance in different T cycles (continued)

C *PIF* family members and *GBSS1* (continued)

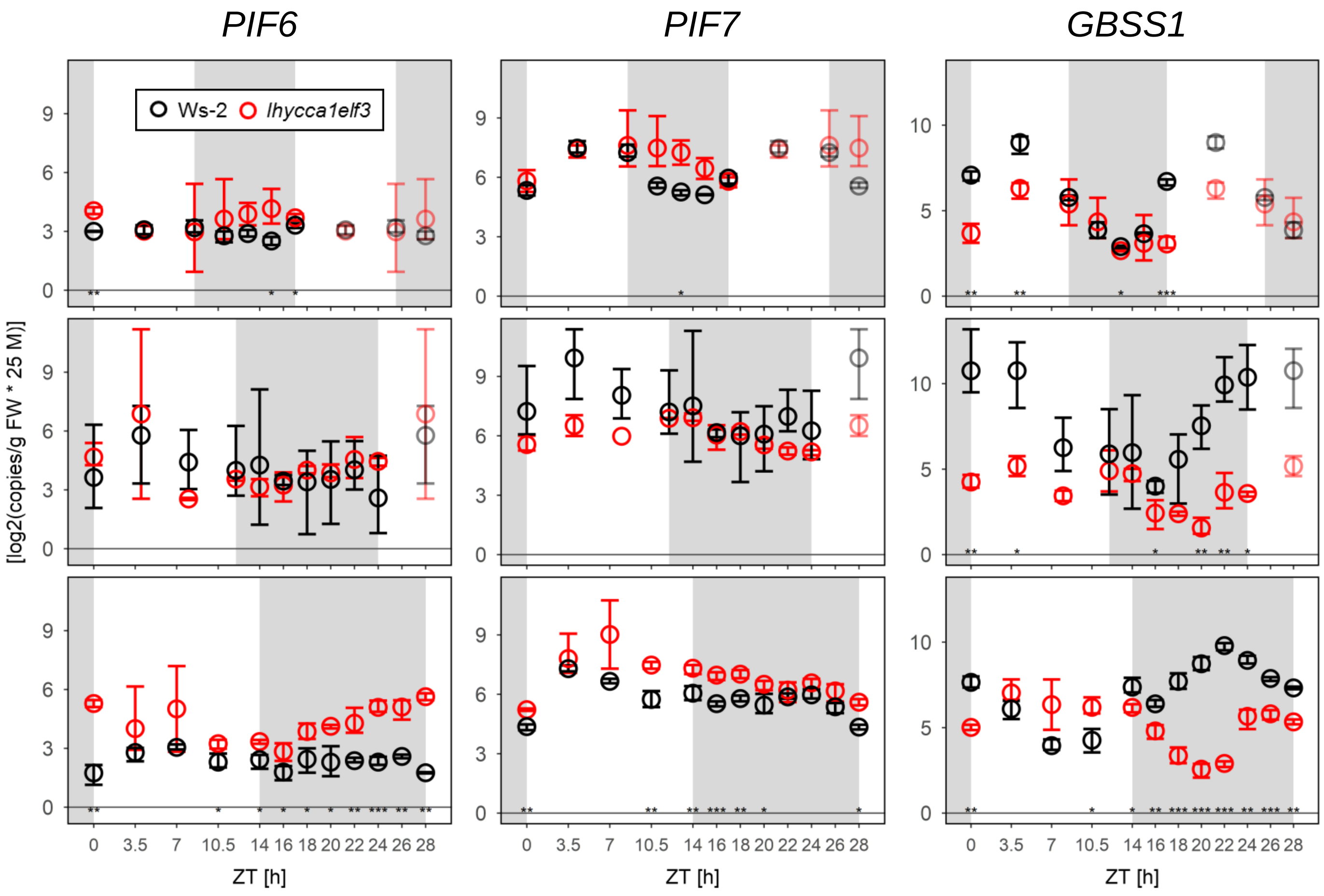

Supplemental Figure S12. Response of transcript abundance in different T cycles (continued)

D Carbon starvation reporter transcripts

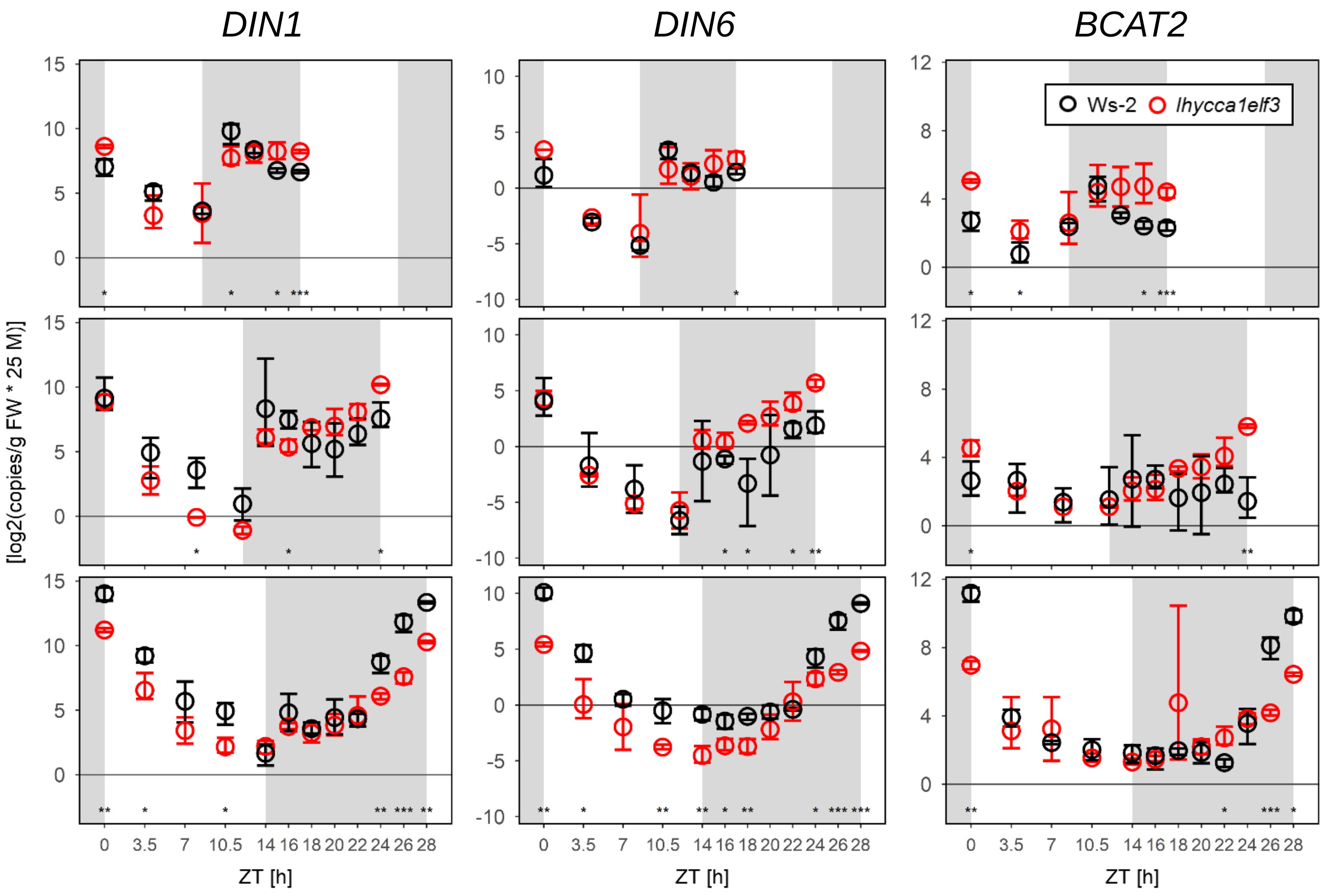

Supplemental Figure S13. Response of transcript abundance in an extended night

Transcripts were measured in the experiment of Suppl. Fig. S1A in the same material that was used to measure starch for Fig. 1 (at 160 µmol m^{-2} s^{-1} irradiance). The Ws-2 data is from Flis et al., 2019. At each time point, 2 to 5 samples were harvested. Transcript abundance was measured by RT-qPCR, adding artificial RNA standard before cDNA amplification to allow absolute quantification. Abundance is given as $\log_2$(copies x 2.5 x10^7 / g FW).

(A) Clock transcripts

(B) *RVE* family members

(C) *PIF* family members and *GBSS1*

Wild-type Ws-2 and *lhy cca1* are indicated by black and red symbols, respectively. The symbols give the mean value, and error bars indicate the bootstrapped 95% confidence interval. Shading indicates the light period (white), the night (grey), and the time interval in which plants had been exposed to the extended night treatment (pale grey). Statistical significance (ANOVA, sum of squares type II) was performed for each genotype separately is indicated by colored asterisks (0 '***' 0.001 '**' 0.01 '*' 0.05; subsequent HSD Tukey's post-test was significant in all cases). "ZT", or "Zeitgeber" from the German language, indicates the time elapsed after the last dawn, in hours.

Supplemental Figure S13. Response of transcript abundance in an extended night

A Clock transcripts (continued on next page)

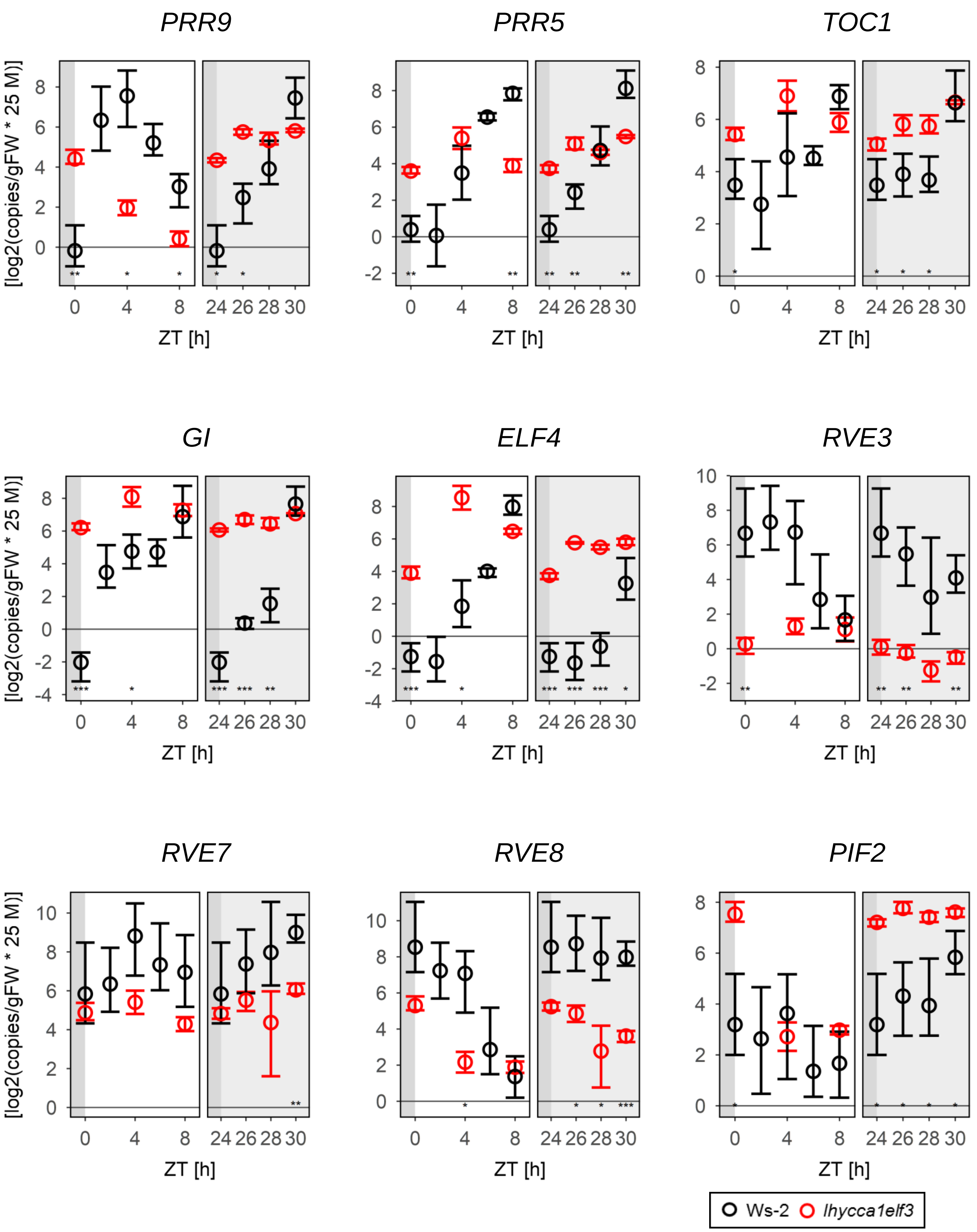

Supplemental Figure S13. Response of transcript abundance in an extended night

A Clock transcripts (continued)

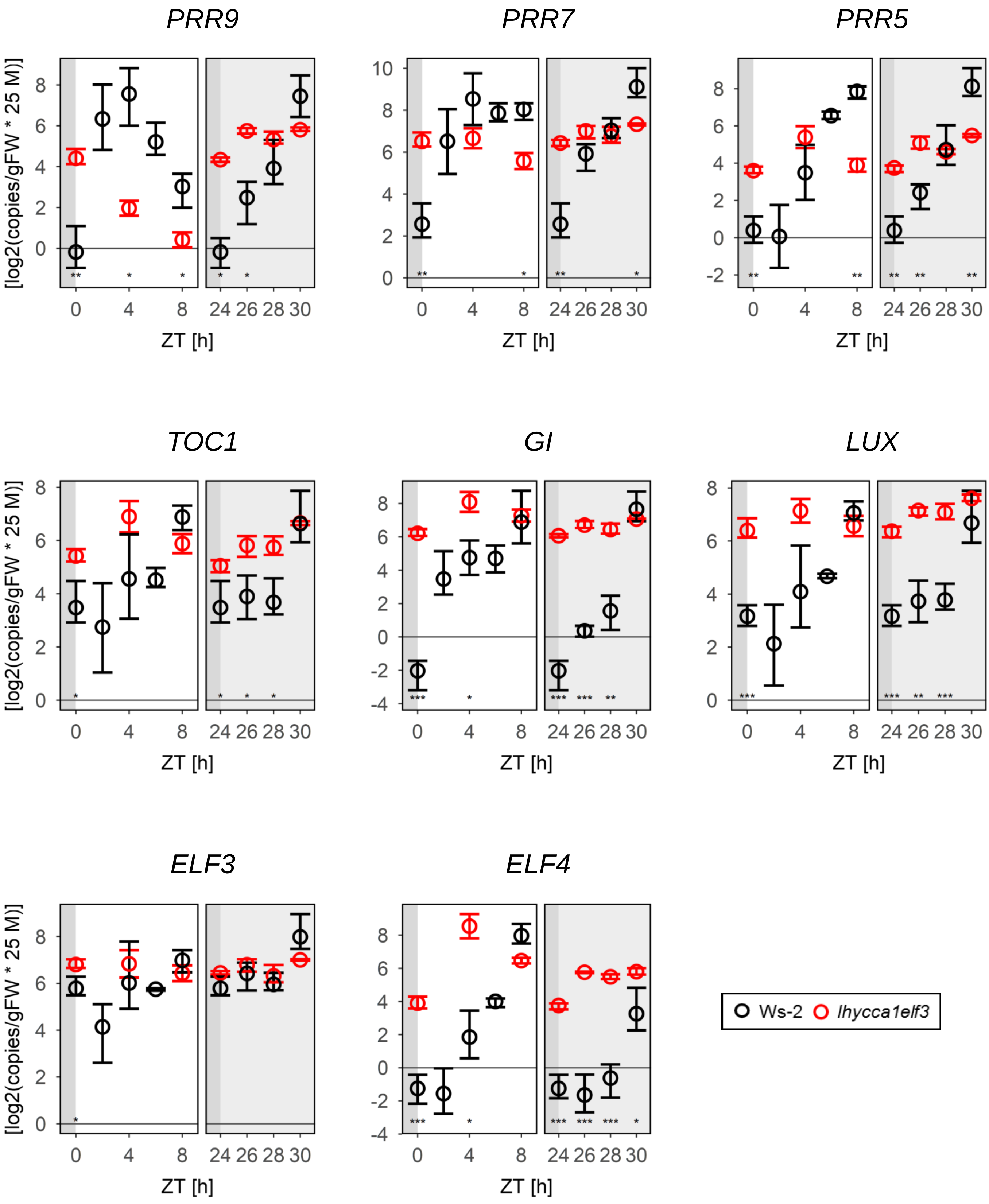

Supplemental Figure S13. Response of transcript abundance in an extended night

B *RVE* family members

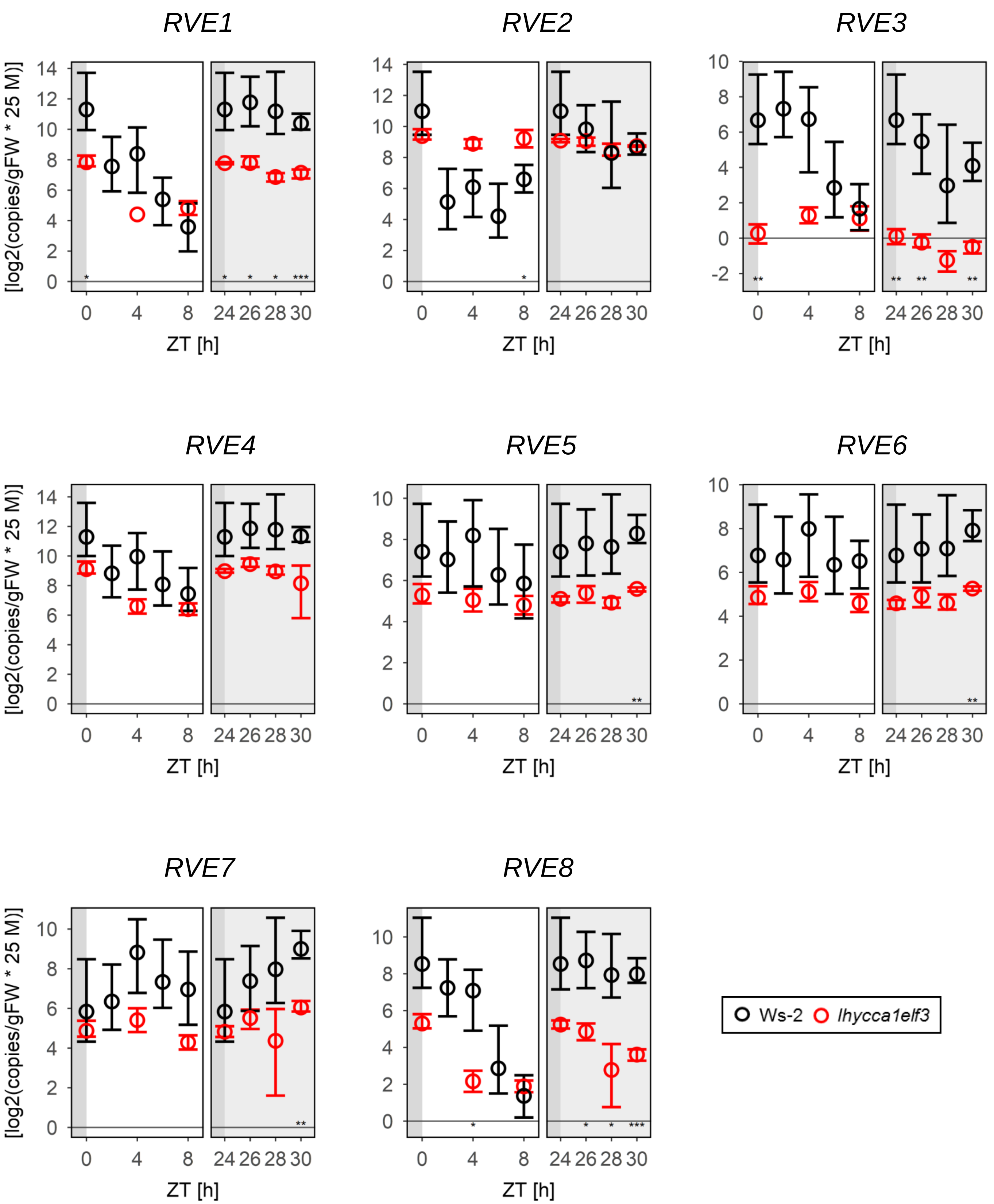

Supplemental Figure S13. Response of transcript abundance in an extended night

C *PIF* family members and *GBSS1*

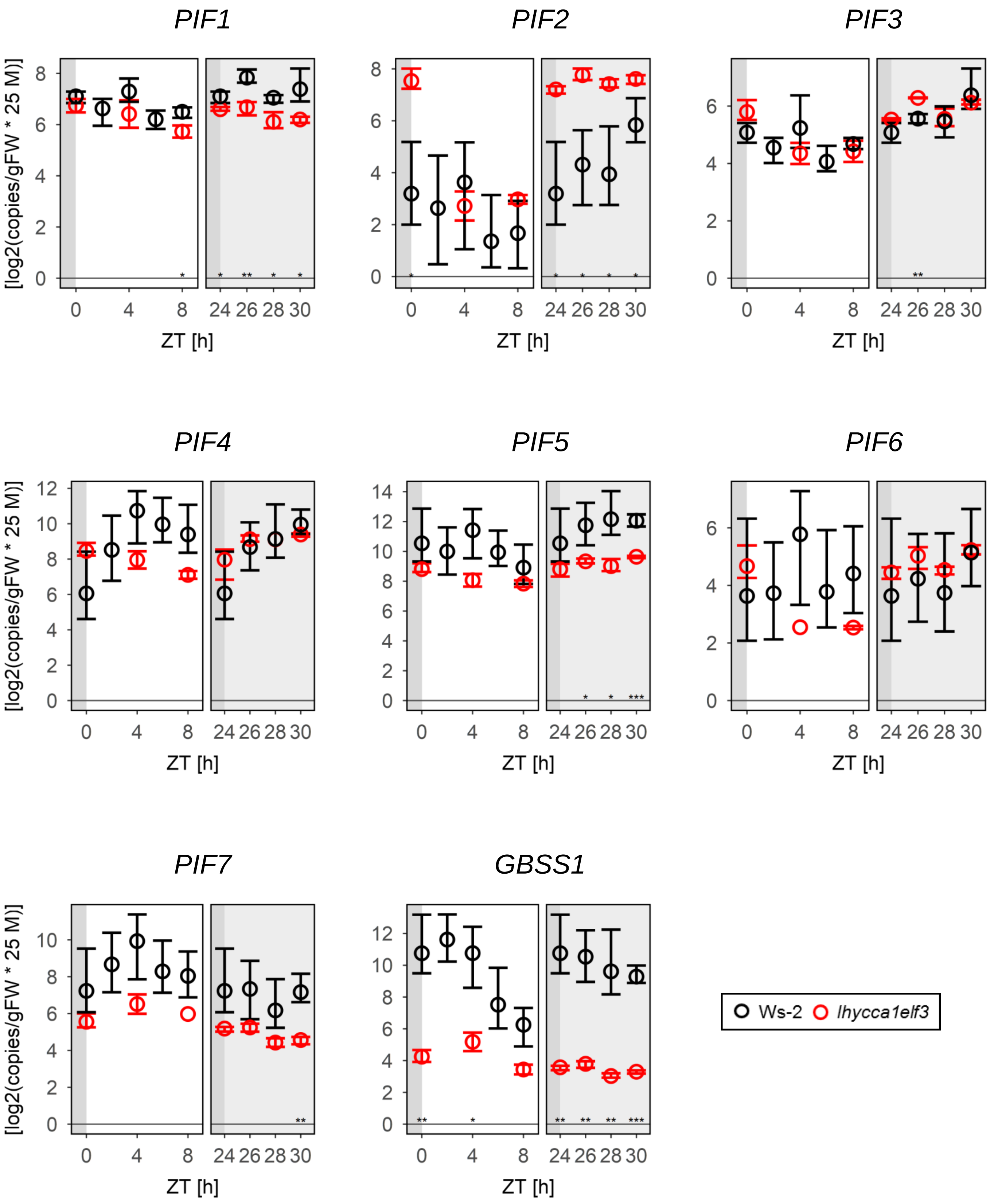

Supplemental Text S1

Dynamics of transcripts after a sudden early dusk and in non T24 cycles, and acute response of clock transcripts to light and dark

Dynamics of transcripts after a sudden early dusk

The ability of wild-type Ws-2 to pace starch mobilisation to around dawn after a sudden early dusk is a particularly striking illustration that starch mobilisation is paced by an internal 24-h rhythmicity (Fig. 4). This response was retained in a weakened form in *lhy cca1 elf3* (Fig. 4, Suppl. Fig. S7) despite this mutant having a very disturbed circadian clock. To learn which, if any, features of the clock might underly this remarkable response, we investigated the response of a large set of clock and clock-related transcripts to a sudden early dusk in wild-type *Ws-2* and *lhy cca1 elf3* (Suppl. Fig.11).

In Ws-2, advancing dusk by 4 h led to only slight changes in the rate of decay of dusk- and evening-phased transcripts (Suppl. Fig.11). Visual inspection indicated a circa 2-h advance in the rise of *LHY* and *CCA1* transcripts at the next dawn. Other transcripts also showed a slight advance of their rise at the end of the night, including *RVE1*, *RVE4*, *RVE8*, *PIF4* and *PIF5*.

In *lhy cca1 elf3* early dusk led to a clear advance in the rise of *PRR9* transcript, which started to rise at about ZT16 instead of ZT20 (Fig. 8A). Early dusk also led to a slightly earlier rise of *PRR5, TOC1, LUX, ELF4* and *ELF3* transcripts at the end of the night (Suppl. Fig. S11), a significant advance in the rise of *RVE1* and *RVE8* transcripts, and a perceptible advance or strengthening of the rise of *PIF3*, *PIF6* and *PIF7* and *GBSS1* transcript compared to the control treatment (Suppl. Fig. S11).

We quantified these responses using mutual information analysis (Fig. 11D), taking an analogous approach to that taken to score for dawn and dusk alignment in Fig. 9. To analyse the response to a sudden early dusk, we aligned the time series based on time elapsed after the previous dawn (which is the way they would usually be plotted) or on time elapsed after the plants were darkened (i.e. advancing the whole time series by 4 h in the early dusk treatment). Fewer scores were returned for the early dusk data set than the photoperiod data set, probably because there were a smaller number of time points and no complete oscillations. In Ws-2 none of the core clock gene set or *RVE* and *PIF* family members lost their dawn-alignment, and for most the response was unrelated to the timing of dusk. Interestingly, *RVE1*, *RVE3* and *PIF4* scored for to certain extent for dusk alignment after a

sudden early dusk. In *lhy cca1 elf3* none of the core clock gene set returned a score, some *RVE* and *PIF* family members scored as partly dawn-aligned and partly dusk aligned, and *GBSS1 s*cored as dusk-aligned.

The analysis indicates that a sudden early dusk does not disturb the dawn dominant response in Ws-2, with only slight advances in the rise of many transcripts during the night and around the next dawn. In *lhy cca1 elf*3 the response is more complex, including a large advance of peak time for many transcripts.

Transcript dynamics in non T24 cycles

The ability of wild-type Ws-2 to pace starch mobilisation to 24 h after dawn in a T17 cycle and to set an initial rate of starch mobilisation that would exhaust starch about 24 h after dawn in a T28 cycle provides another line of evidence that starch mobilisation is paced by an internal 24-h rhythmicity in wild-type Arabidopsis (Fig. S2, see also Graf et al., 2010). This response was largely retained in *lhy cca1 elf3* (Fig 2, Suppl. Fig. S5) To learn which residual features of the clock might underly this unexpected robustness, we investigated the diel responses of clock, *RVE* and *PIF* family members and *GBSS1* transcripts in T17 and T28 cycles (Suppl. Fig. S12A-C). The data sets were aligned on dawn. To aid visual inspection, the T17 and T24 time series were concatenated to provide plots that extended to 28 h after the initial dawn.

In wild-type Ws-2, the oscillations of a given transcript in T17, T24 and T28 cycles were largely superimposed. For example, at external dawn in a T17 cycle most transcripts were at levels resembling those at ZT17 (the middle of the night) in a T24 cycle, whilst at external dawn in a T28 cycle most transcripts were at levels resembling those after 4 h of light in a T24 cycle. This shows that clock progression is largely independent of T-cycle duration in Ws-2.

A different picture emerged in *lhy cca1 elf3*. Analysis was complicated by the attenuated oscillations, with only *PRR9* and *ELF4* showing large oscillations of transcript abundance in all three T-cycles. The level of *PRR9* transcript at external dawn in a T17 cycle differed from that ZT17 in a T24 cycle, and the level of both *PRR9* and *ELF4* transcript at external dawn in a T28 cycle differed from those after 4 h of light in a T24 cycle.

More generally, the attenuation or loss of most oscillations that was seen in *lhy cca1 elf3* in T24 cycles was also seen in T17 and T28 cycles. Of the transcripts that did oscillate in *lhy*

cca1 elf3, some showed a clear difference between T cycles, for example the peak of *PRR5* transcript at ~ZT3.5 was lost in T28. Furthermore, several transcripts showed a light-dependent rise (*TOC1, GI, ELF4, LUX, PIF2, PIF7, GBSS1*) or fall (*RVE4, RVE8*) after dawn that was independent of the time from the previous dawn.

Deeper statistical analysis of the T-cycle time series by mutual information analysis or ANOVA was hampered by the relatively low number of available time points, and the fact that the number of time points differed between T-cycles

Overall, qualitative analysis indicated that, compared to wild-type Ws-2, the residual clock in the triple mutant does not retain robust behaviour across different T-cycles.

We also analysed *DIN1*, *DIN6* and *BCAT*2 transcript abundance (Suppl. Fig. 12D). We did this to learn if incomplete exhaustion of starch in a T17 cycle and premature exhaustion of starch in a T28 cycle led to altered abundance of these C-starvation markers.

In a T24 cycle, *lhy cca1 elf2* had slightly higher abundance of these transcripts during the night than wild-type Ws-2 (as already mentioned in the main text, see Fig. 6). This trend was visible at the end of the night, when transcript abundance rose more markedly in *lhy cca1 elf3* than in Ws-2. This slightly higher transcript abundance may reflect the slightly lower sugar levels during the night in *lhy cca1 elf3* compared to Ws-2 (as already mentioned in the main text, see Suppl. Fig. S8).

In a T17 cycle, transcript abundance in *lhy cca1 elf3* was again slightly higher than in Ws-2. The rise towards the end of the night was absent, probably because starch was not exhausted in this short T-cycle (see main text and Fig. 2A).

In a T28 cycle, in wild-type Ws-2 the C-starvation marker transcript started to rise by ZT24 and rose sharply at ZT26 and ZT28, as expected because starch content is low and the rate of mobilisation slows markedly after ZT22-24 (as discussed in the main text, see Fig 2A and Suppl. Fig. S5). This rise of the C-starvation markers after ZT22-24 was less marked in *lhy cca1 elf3*, possibly because the triple mutant exhibited an even less linear pattern of starch mobilisation (see Fig 2A and Suppl. Fig. S5), resulting in starch mobilisation rates after ZT22-24 that were even slightly slower than in Ws-2.

It might also be noted that there is a consistent trend to transiently elevated of *DIN1*, *DIN6* and *BCAT*2 transcript abundance at 2 h after dusk, especially in T17 and T24 cycles. This probably reflects a short delay until rapid rates of starch mobilisation are established after a

sudden transition from light to darkness (Annunziata et al., 2017 [1]). This results in a transient trough of sucrose, Glc6P and even polysome loading (Pal et al., 2013 [2]).

Acute response of clock transcripts to light and dark

Given that the residual clock in *lhy cca1 elf3* was so strongly disturbed we wondered how many of the observed responses of transcript abundance were acute responses light or other inputs. Close inspection of Suppl. Figs. S9 and S11-S12 reveals a small but consistent decrease in *PRR9*, *PRR5*, *ELF4*, *RVE3*, *RVE7*, *RVE8*, and *PIF2* transcript abundance after darkening *lhy cca1 elf3*. These might represent acute responses to darkness, because these responses were absent or weaker in wild-type Ws-2. Furthermore, many of these transcripts also showed changes after illumination in T-cycles that were unrelated to the time elapsed since the previous dawn (see previous subsection and Suppl. Fig. S12).

These observations led to us to ask whether the response of transcripts after dawn in *lhy cca1 elf3* may also be partly an acute response to light. We grew plants in a 12 h light / 12 h dark cycle, subjected them to a sudden 6 h extension of the night and compared the responses of clock transcripts between ZT24-ZT30 (i.e. after extending the night) with those between ZT0 and ZT6 (i.e., in control plants that remained in a 12-h light / 12 h dark photoperiod (Suppl. Fig. S13). Wild-type Ws-2 showed similar responses after illumination in the control 12 h light / 12 h dark cycle and in sudden extended night treatment, as expected if the oscillations of transcripts are driven by an endogenous clock. A different picture was found for *lhy cca1 elf3*. In particular the *PRR9* transcript declined after illumination but stayed high in continued darkness, and the rise and subsequent decline of the *ELF4* transcript in the light was attenuated in the extended night treatment. On the other hand, the rise of *PRR5*, *GI* and *TOC1* transcripts between ZT0 and ZT4 was also seen at ZT24-ZT30 in an extended night, implying these may be endogenous responses. It is noteworthy however that these transcripts did exhibit apparent acute responses to light or darkness in other treatments, for example after illumination in different T-cycles (see above)

---

[1] Annunziata, M.G., Apelt, F., Carillo, P., Mengin, V., Kraus, U., Feil, R., Mengin, V., Lauxmann, M-A., Köhl, K., Nikoloski, Z., Stitt, M., and Lunn, J.E. (2017). Getting back to nature: a reality check for experiments in controlled environments. J. Exp. Bot 68, 4463-4477.

[2] Pal, S.K., Liput, M., Piques, M., Ishihara, H., Martins, M.C.M., Sulpice, R., van Dongen, J., Yadav, U.P., Lunn, J.E., Usadel, B., Schulze, W.X., Stitt, M. (2013) Diurnal changes of polysome loading track sucrose content in the rosette of wildtype Arabidopsis and the starchless *pgm* mutant. Plant Physiol 162, 1246-1265.

Taken together, Suppl. Fig. S13 shows that the diel oscillations in *lhy cca1 elf3* are driven at least in part by acute responses to illumination or darkening. These might include light signalling, but a contribution from indirect effect like changes in sugar levels might in principle also contribute.

Supplemental Text S2

TAIR, UniPROT, and EMBL accession number/identificAtion

Sequence dAta from genes and proteins cited in this article can be found in the TAIR, the UniPROT and the EMBL dAta libraries under accession listed in Table 1 below.

Table 1 - Accession identifiers per gene

Gene	TAIR id	UNIPROT id	EMBL id
LHY	At1g01060	A0A178W761	OAP13996
CCA1	At2g46830	P92973	ANM61754
PRR9	At2g46790	Q8L500	AEC10754
PRR7	At5g02810	Q93WK5	AED90520
PRR5	At5g24470	Q6LA42	AED93314
TOC1	At5g61380	Q9LKL2	BAB08493
GI	At1g22770	Q9SQI2	AEE30286
ELF3	At2g25930	O82804	AEC07774
ELF4	At2g40080	O04211	AEC09773
LUX	At3g46640	F4J959	AEE78188
ZTL	At5g57360	F4KAN2	AED96892
PIF1	At2g20180	Q8GZM7	ANM61700
PIF2	At2g46970	Q8L5W8	AEC10779
PIF3	At1g09530	O80536	ABP96435
PIF4	At2g43010	Q8W2F3	ABP96467
PIF5	At3g59060	Q84LH8	AEE79868
PIF6	At3g62090	A0A178VFI3	CAD5326512
PIF7	At5g61270	Q570R7	ANM70162
RVE1	At5g17300	F4KGY6	AED92410
RVE2	At5g37260	F4K5X6	AED94158
RVE3	At1g67500	A0A384LJW3	OAP13222
RVE4	At5g02840	A0A178U8Z5	CAD5330636
RVE5	At4g01280	C0SVG5	AEE82003
RVE6	At5g52660	Q8H0W3	AED96246
RVE7	At1g18330	B3H5A8	AEE29702
RVE8	At3g09600	Q8RWU3	ANM65370
bZIP63	At5g28770	B9DGI8	AED93832
GBSS1	At1g32900	Q9MAQ0	AAF31273
DIN1	At4g35770	A0A178UYD5	CAD5330060
DIN6	At3g47340	A0A178VBT4	OAP02412
